# Household determinants of healthcare utilisation in three informal settlements in Freetown, Sierra Leone: a cross-sectional survey

**DOI:** 10.1136/bmjopen-2025-108022

**Published:** 2026-03-03

**Authors:** Samira Sesay, Ibrahim Juldeh Sesay, Sia Morenike Tengbe, Haja Wurie, Sullaiman Fullah, Dora Vangahun, Ibrahim Gandi, Noemia Teixeira de Siqueira Filha, Rajith W D Lakshman, Abu Conteh, Samuel Saidu, Braima Koroma, Bintu Mansaray, Helen Elsey, Lana Whittaker, Laura Dean, Neele Wiltgen Georgi, Motto Nganda, Francis Refell, Joseph MacCarthy, Alastair H Leyland, Rachel Tolhurst, Eliud Kibuchi

**Affiliations:** 1 College of Medicine and Allied Health Sciences, University of Sierra Leone, Freetown, Sierra Leone; 2 Sierra Leone Urban Research Centre (SLURC), Freetown, Sierra Leone; 3 Sierra Leone Ministry of Health and Sanitation, Freetown, Sierra Leone; 4 Centre of Dialogue on Human Settlement and Poverty Alleviation, Freetown, Sierra Leone; 5 Department of Health Sciences, University of York, York, UK; 6 Institute of Development Studies, Brighton, UK; 7 Department of International Public Health, Liverpool School of Tropical Medicine, Liverpool, UK; 8 Pediatrics, King Harman Road Maternal and Child Health Hospital, Freetown, Sierra Leone; 9 School of Health and Wellbeing, University of Glasgow, Glasgow, UK

**Keywords:** Health Services Accessibility, PUBLIC HEALTH, Health Equity

## Abstract

**Abstract:**

**Objective:**

Healthcare utilisation (HU) is key to improving the health of residents in urban informal settlements. This study aimed to explore household-level factors influencing HU among informal settlement households in Freetown, Sierra Leone.

**Design:**

Cross-sectional survey.

**Setting:**

Three informal settlements (Cockle Bay, Dwarzark and Moyiba) in Freetown, Sierra Leone.

**Participants:**

Primary data from 4871 households were collected during the Health and Wellbeing survey conducted between April and May 2023, targeting households with adults aged 18 years and older.

**Primary outcome measures:**

The primary outcomes were households HU both within and outside informal settlements. Household-level predisposing and enabling explanatory variables were derived from Andersen’s Behavioural Model of HU.

**Results:**

Disability in households increases HU within settlements (especially in Dwarzark, 13% and Moyiba, 10%) but is less likely outside. Households engaged in income-generating activities are more likely to seek healthcare within settlements, but 12% less likely outside in Cockle Bay and Dwarzark. Food insecurity decreases HU within Dwarzark (9%) and increases HU outside by 174% in Moyiba. Longer water fetching times and water shortages were associated with higher HU (between 6% and 16%) within settlements, especially in Cockle Bay and Dwarzark. Clean water sources (eg, piped dwelling, bowser, surface, bottled) were consistently associated with higher HU both within and outside settlements. Shared sanitation facilities (such as shared toilets) were positively associated with HU both within and outside settlements, particularly in Dwarzark and Moyiba. Households with income from fishing, informal salaried work and bike riding showed higher HU both within and outside settlements, especially in Dwarzark and Moyiba.

**Conclusions:**

We identified strong settlement-specific patterns of household-level factors that influence HU both within and outside Freetown’s informal settlements. These findings provide a foundation for developing targeted policies such as strengthening local services, addressing affordability and accessibility barriers and supporting vulnerable occupation groups.

**STRENGTHS AND LIMITATIONS OF THIS STUDY**

- Our findings provide valuable insights into the household-level determinants of healthcare utilisation (HU) across households in informal settlements in Freetown, Sierra Leone, drawing on the primary data from the first large cross-sectional survey conducted in these settings.
- The study identified settlement-specific associations between predisposing and enabling household-level factors and HU both within and outside Freetown’s informal settlements.
- The analysis was limited to household-level factors that are associated with HU and did not include individual-level factors due to the nature of the data.
- As the study focused on three purposively selected informal settlements, the findings are not generalisable to all of Freetown’s more than 60 informal settlements.

## Introduction

 The need to provide timely and adequate access to healthcare for people living in urban informal settlements in low-income and middle-income countries (LMICs) is of paramount importance, as they tend to have poorer health outcomes compared with their counterparts in rural and other urban areas.^1^,^3^ These poorer health outcomes are largely due to exposure to inadequate living conditions including limited basic facilities, low-quality housing, overcrowding, unsafe residential conditions and pollution.^1 3^ Despite being at high risk of adverse health outcomes, which in turn necessitates a greater need for healthcare utilisation (HU), no studies have examined the association of household-level factors and HU in these settings, even though they strongly shape access and affordability through shared vulnerability, constraints and decision making beyond what individual factors can capture.^2 4^

HU is defined as accessing health service(s) when needed^5^ and is influenced by individual predisposing, enabling, lifestyle and need factors, as described in Andersen’s Behavioural Model framework.^6^ Predisposing factors consist of existing conditions that influence an individual’s decision to use or not use a healthcare service, even though they may not be directly responsible for the decision (eg, age and sex).^6 7^ Enabling factors impede or facilitate the use of health services (eg, income level, employment status).^6 7^ Lifestyle factors are practices such as smoking and alcohol consumption, which affect an individual’s health status and their future healthcare needs. Finally, need factors represent the actual need for healthcare services (eg, existing health conditions).^6 7^

Studies have used Andersen’s Behavioural Model to guide the selection of factors associated with HU in informal settlements at the individual level.^8^,^10^ A systematic review by Park *et al*^2^ confirmed its applicability in this context, highlighting the influence of individual-level factors such as personal and biological characteristics, socioeconomic, cultural, religious, health systems, legal and cognitive factors on HU.^2^ From this review, it is clear that many studies from LMICs have examined how predisposing, enabling and need factors at the individual level influence HU in the informal settlements to a significant extent.^2^ However, a critical research gap remains regarding the role of household-level factors in HU within these settings.^2^ Household-level factors can be incorporated into Andersen’s Behavioural framework, particularly in the context of informal settlements, because shared living conditions within small and crowded spaces lacking basic services often influence resource pooling and healthcare-seeking decisions.^2^

For example, household characteristics such as water sources, type of toilet used, family size and the head of household gender can be classified as predisposing factors, as they may affect the likelihood of seeking healthcare.^11^ Enabling factors, such as household engagement in income-generating activities and the types of income sources, may determine whether household members can afford healthcare when needed. Finally, existing conditions such as chronic illnesses or disabilities within the household can increase the likelihood of HU but also strain household resources, potentially limiting access for other members. In summary, understanding how household-level factors are associated with HU in informal settlements provides a more complete picture of utilisation patterns and can facilitate the design of effective, targeted interventions.

Moreover, understanding whether these predisposing and enabling household-level factors differ when healthcare is sought within an informal settlement (ie, inside the settlement boundaries) or outside (ie, beyond the boundaries) would support the design of targeted interventions to improve HU. This distinction is crucial, as it enables examination of how household-level factors influence healthcare-seeking decisions based on ease of access, affordability, service availability and quality of care.^12^ This issue is particularly important in Freetown, Sierra Leone, a low-income West African city, where no previous research has examined the association between household-level factors and HU in informal settlements.

Freetown, the most urbanised city in Sierra Leone, with a population of over one million based on the 2015 census,^13^ has experienced rapid expansion of informal settlements, which now cover 36% of the city’s land area. This growth has been largely driven by rural-to-urban migration, as individuals move in search of better economic opportunities.^14^ Within these settlements, households navigate a fragmented healthcare landscape, relying on a mix of public and private providers, informal drug sellers and private nurses to meet their healthcare needs.^15^

Guided by Andersen’s Behavioural Model, this study examines how household-level predisposing and enabling factors influence inequalities in HU. It explores associations between household-level factors and HU both within and outside three informal settlements (Cockle Bay, Moyiba and Dwarzark) in Freetown, Sierra Leone. HU is assessed for all members of each household, and the findings provide context-specific insights to inform policies aimed at improving healthcare access and reducing inequalities in Freetown.

## Methods

### Study setting

**Figure 1:**
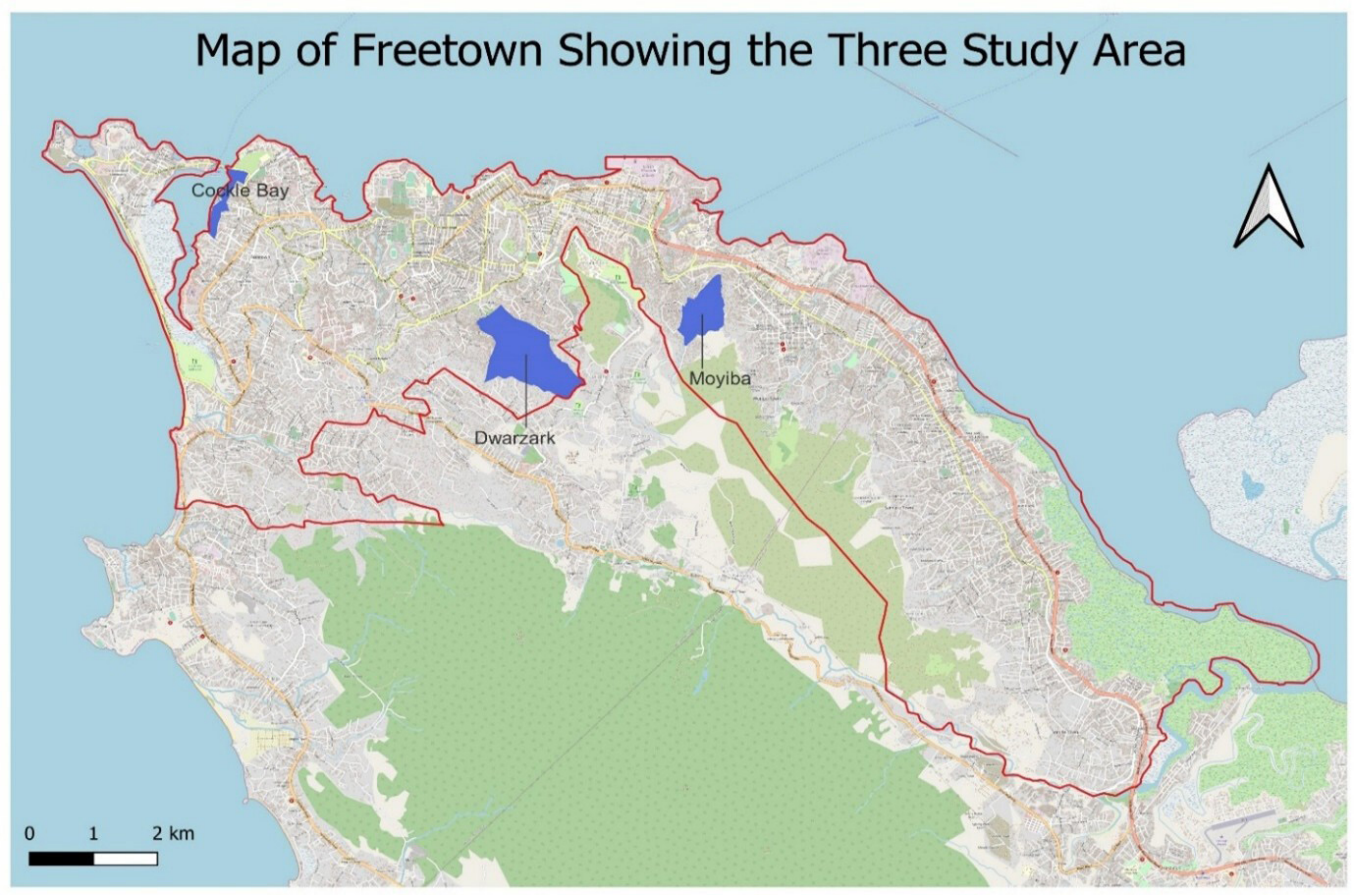
A map of three study areas (in blue) Sesay *et al*.^15^

This study was conducted in three informal settlements in Freetown, Sierra Leone: Cockle Bay, Moyiba and Dwarzark (figure 1). Sierra Leone, located in West Africa, has one of the lowest Human Development Index scores, reflecting significant challenges in health, education and living standards.^16 17^ These settlements were purposively selected for primary data collection because of their diverse spatial, social and economic characteristics.

Moyiba and Dwazark are hillside settlements in the eastern and central parts of Freetown, respectively, both facing perennial challenges of healthcare access.^17^ Dwazark, with an estimated 16 500 residents in 2018, has nearly 65% of its population under 30 years old.^17^ Land ownership disputes in Dwarzark have contributed to poor housing, inadequate roads and sanitation issues due to limited infrastructure development.^17^ Most residents are engaged in informal employment, such as petty trading and stone mining. The majority of Dwarzark residents reported using public (88%) and private (77%) healthcare facilities, followed by drug sellers (54%) and private nurses (52%).^15^

Moyiba, home to around 37 000 residents, is characterised by low income levels, poor environmental conditions, housing challenges and high unemployment, with many engaged in stone quarrying and petty trading.^17^ The majority of Moyiba residents reported seeking healthcare from public health facilities (82%), drug sellers (62%), private nurses (45%) and traditional healers (37%).^15^

Cockle Bay, located along the Aberdeen Creek on the western coast, is a low-lying settlement with approximately 16 000 residents.^17 18^ The land is owned by the local municipality, and the settlement suffers from poor drainage and inadequate infrastructure, making it highly vulnerable to flooding, waterborne diseases (especially cholera) and fires. Most residents rely on fishing and cockle production for their livelihoods.^17^ Cockle Bay residents reported relying on drug sellers (56%) and private facilities (35%) to access healthcare, while 55% did not seek care due to the lack of available health facilities.^15^

### Data

Primary data collection for the 2023 Freetown Informal Settlements Health and Wellbeing Survey was conducted in Cockle Bay, Dwarzark and Moyiba between April and May 2023, targeting households with adults aged 18 years and older.^15^ The survey questions were codesigned by researchers and community-based fieldworkers, informed by findings from a prior participatory and qualitative exploratory study conducted in 2020 and 2021 as part of the Global Challenges Research Fund Accountability for Informal Urban Equity Hub.^15 19^ Residents identified HU as a key societal issue.^20^

The sample size was determined based on the estimated proportion of households facing barriers to HU, which was 0.47 from a pilot survey. We used a margin of error of 0.03 and a design effect of 10 to account for the hybrid sampling technique (combining probability and non-probability sampling due to lack of an effective sampling frame), a critical value of 0.05 (95% CI), and a non-response rate of 10%. The final sample size was 4883 households, and further details about the sample size computation are provided in Sesay *et al*.^15^

The selection of households for interviews was conducted in multiple stages. First, households were proportionally allocated based on the 2018 estimated population: Cockle Bay (n=1251), Dwarzark (n=1321) and Moyiba (n=2312). Each settlement’s households were then equally distributed across different zones: Cockle Bay (313 households per zone × 4 zones), Dwarzark (111 households per zone × 12 zones) and Moyiba (232 households per zone × 10 zones). In each zone, a random household along a chosen outward direction from an identified landmark was selected as the starting point for interviews. Landmarks included mosques, cinemas, community centres and water points, with settlement boundaries identified during a GIS mapping study conducted earlier.^20^

Next, the closest household to the landmark was selected, and interviews were conducted with every $k^{th}$ household until the zone boundary was reached. The $k^{th}$ value was calculated by dividing the number of households by the number of landmarks in that zone. One consenting adult (18 years or older), either the head of the household or the most senior household member, was interviewed in each household.

The interviews were conducted face-to-face by 35 co-researchers and 18 community mobilisers, all recruited from the study areas for their familiarity with the communities. The survey team underwent a 4-day training programme covering survey procedures, questionnaire content, the REDCap tool, ethical research considerations, safeguarding and sampling techniques. A total of 5121 households were initially interviewed. After data cleaning with co-researchers, which removed 250 (4.9%) duplicate records, the final sample comprised 4871 complete records.

### Outcome variables

We considered two primary outcomes: HU within informal settlements and HU outside informal settlements. The first outcome assessed whether the respondent or any household member sought healthcare services (both formal and informal) within their informal settlement in the past month. This measure helps identify household-level factors that influence HU within boundaries of informal settlements. The second outcome assessed whether the respondent or any household member sought healthcare services (both formal and informal) outside their informal settlements in the past month. This outcome provides insights into household-level factors that influence HU beyond the boundaries of informal settlements. Moreover, we conducted a sensitivity analysis by combining HU within and outside settlements to provide a more comprehensive view of household-level factors associated with overall HU.

### Explanatory variables

Our choice of household-level explanatory variables was based on Andersen’s Behavioural Framework, with guidance from Park’s study on factors that influence HU in informal settlements,^2 6^ categorising them into predisposing and enabling factors.

Predisposing factors (household characteristics influencing HU) included head of household gender, family type, presence of disability in the household (including whether the head of household is disabled), food security status (measured by whether a household could eat the kinds of food they preferred), length of residence in the settlement and environmental conditions (eg, exposure to disasters). We also accounted for water access (eg, piped water in dwellings, wells, public taps, rainwater or sachet water) and sanitation facilities (eg, flush toilets, pit latrines, bucket toilets, open defecation). Waste disposal methods (eg, community waste collection, dumping sites, disposal of waste in drainages, or disposal into the sea for those living in coastal shacks) and whether households pay for waste collection were also considered.

**Figure 2:**
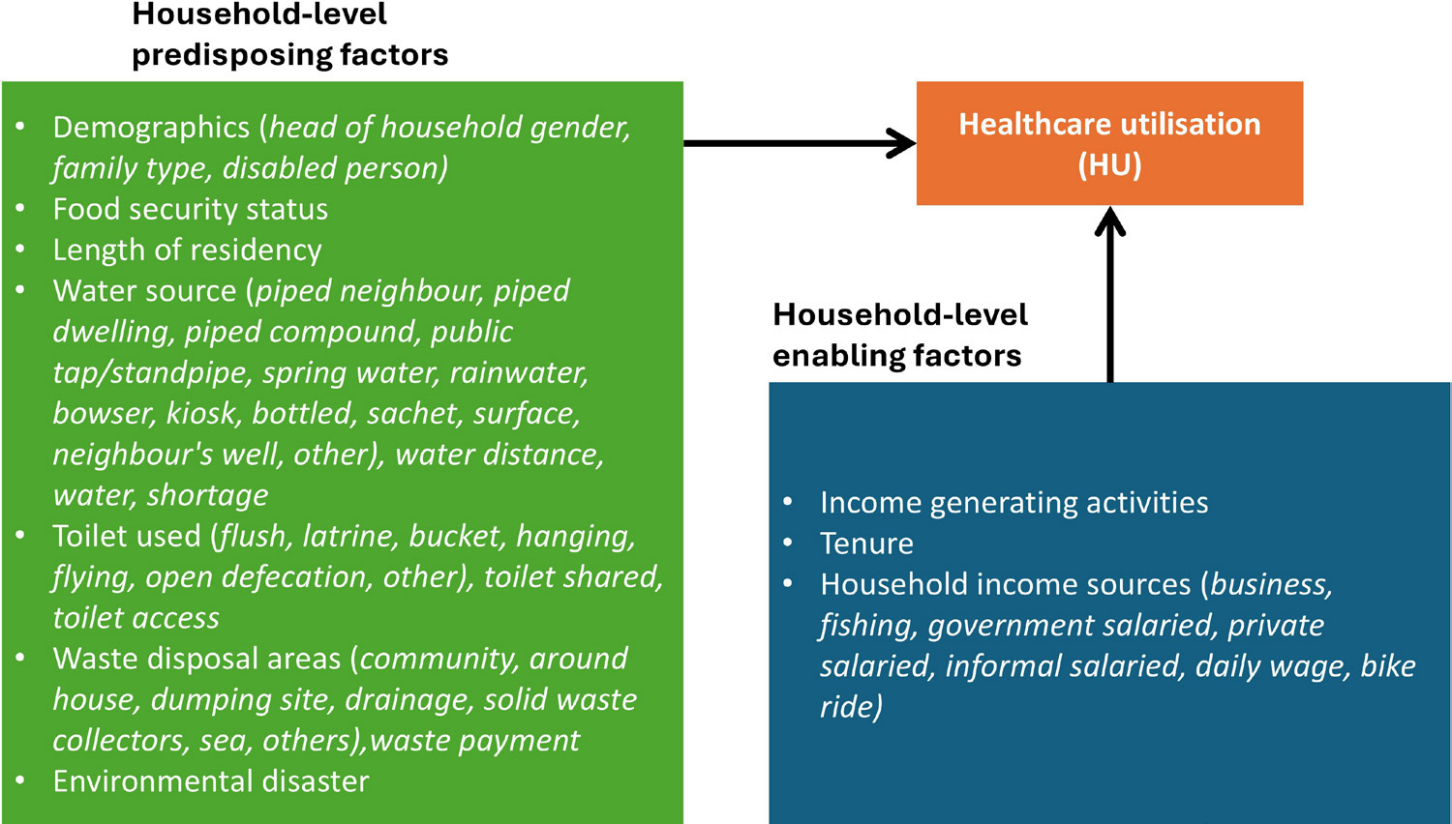
Application of Andersen’s Behavioural Model to HU in informal settlements, showing household-level predisposing and enabling factors. The arrow indicates an associative, not causal, relationship.

Enabling factors (socioeconomic conditions that facilitate or hinder HU) included household tenure (ownership or rental status), income-generating activities and sources of household income. Specifically, we distinguished between private salaried work (formal employment with fixed wages, such as company jobs), business (self-employment or entrepreneurship) and informal salaried work (casual or irregular wage labour). Other sources that were considered include fishing, daily wages, stone mining and bike riding (commercial motorcycle transport, commonly known as ‘okada’). Figure 2 shows the household-level predisposing and enabling factors considered within Andersen’s Behavioural Model.

### Data analysis

Descriptive statistics for categorical variables were analysed using χ² tests and p values to assess whether selected household-level characteristics were significantly associated with HU across the three study areas. We assessed collinearity among explanatory variables using Cramér’s V to avoid multicollinearity in the fitted models.^21^ All household-level explanatory variables were initially included in the final model, which was considered the global model.^22^ For variables that were correlated, we retained only one and excluded the rest. Moreover, variables with small cell sizes were excluded to ensure stable estimation of coefficients.

We used a robust Poisson regression model to examine the association between household-level explanatory variables and HU.^23^ The robust Poisson regression is a semiparametric estimator of log-binomial model and makes no assumptions about the distribution of the outcome (ie, HU), except for the functional form of the outcome probability relative to the explanatory variables.^23^ Moreover, the robust Poisson model is preferred because it estimates adjusted relative risk (RR) as an aggregated effect measure (ie, for everyone in a household), rather than individual risk, as in logistic regression.^24^ We included household size as an offset since it is associated with HU at the household level.^25^ Given the three informal settlements included in this study have distinct geographical characteristics and differ in the availability of health centres, we fitted separate regression models for each study area. This approach provides a clearer understanding of association patterns at the settlement level, which is crucial, since interventions can be more effectively tailored to each settlement.

To ensure consistency and analytical clarity across analyses, we reclassified ‘not applicable’ responses as ‘no’, treating the absence of the household factor as a valid negative response. Additionally, any response marked as ‘don’t know’ was treated as missing values. Given the relatively small proportion of missing data (255 respondents; 5.2% of the sample), we proceeded with a complete case analysis. We also computed absolute risks (AR), which are predicted probabilities of HU for each categorical level of a variable, and presented them in the online supplemental material file 1. All analyses were conducted using R V.4.0.12,^26^ and the code is available at the following link https://github.com/Kibuchi-eliud/Household-level-determinants-of-healthcare-utilisation-in-informal-settlements-in-Freetown.git in GitHub.

### Patient and public involvement

This study employed community-based participatory research where community members collaboratively identified HU as a key issue affecting them. Survey questions were codesigned by researchers and community fieldworkers, drawing on prior qualitative findings from the community.

## Results

Table 1 presents χ^2^ test statistics and p values for predisposing and enabling household-level factors associated with HU within and outside informal settlements, and highlights characteristics with statistically significant associations. Head of household gender was significantly associated with HU within settlements in Moyiba. The presence of a disabled person in the household showed significant association with HU outside the informal settlement in both Cockle Bay and Moyiba. Engagement in income-generating activities was significantly associated with HU within settlements in Cockle Bay and Moyiba, and with HU outside settlements in Cockle Bay alone. Food security showed a significant association with HU outside settlements across all three informal settlements. The association between water sources and HU varied across study settlements and depending on whether healthcare was sought within or outside the settlement. For HU within Cockle Bay, significant associations were observed with the use of public taps, compound wells, spring water, rainwater and sachet water. In Moyiba, piped compound water, kiosk water and sachet water were significantly associated with HU within settlements. For HU outside settlements, significant water sources included piped neighbour, spring water and rainwater in Cockle Bay; sachet water and other sources were in Dwarzark; and kiosk, bottled and surface water in Moyiba. Water distance was significantly associated with HU in Cockle Bay and Moyiba, for both within settlement and outside settlement contexts. Water shortage was significantly associated with HU in Cockle Bay and Dwarzark, across both within settlement and outside settlement contexts.

**Table 1:** χ^2^ statistics and p values for household-level predisposing and enabling factors by healthcare utilisation, both within and outside informal settlements across Cockle Bay, Dwazark and Moyiba

Variable name		Health utilisation within informal settlement			Health utilisation outside informal settlement		
		χ^2^ (p value)			χ^2^ (p value)		
		Cockle Bay	Dwarzark	Moyiba	Cockle Bay	Dwarzark	Moyiba
Predisposing factors							
Head of household gender		3.42 (0.06)	0.05 (0.83)	5.33 (0.02)*	0.32 (0.57)	2.06 (0.15)	2.91 (0.09)
Disability in household		0.75 (0.39)	3.10 (0.08)	1.11 (0.29)	5.79 (0.02)*	13.56 (0.01)*	8.07 (0.01)*
Family type		7.98 (0.02)*	1.71 (0.42)	3.53 (0.17)	31.38 (0.01)*	7.40 (0.02)*	3.21 (0.20)
Food security		10.18 (0.01)*	7.20 (0.01)*	0.03 (0.87)	4.78 (0.03)*	5.03 (0.02)*	210.78 (0.01)*
Length of residence		4.24 (0.24)	0.37 (0.95)	27.36 (0.01)*	6.64 (0.08)	0.45 (0.93)	1.64 (0.65)
Water source	Piped neighbour	66.22 (0.01)*	0.02 (0.89)	8.04 (0.01)*	7.11 (0.01)*	0.17 (0.68)	0.33 (0.57)
	Piped dwelling	2.32 (0.13)	1.58 (0.21)	0.02 (0.89)	0.11 (0.74)	4.30 (0.04)*	1.37 (0.24)
	Piped compound	8.42 (0.01)*	1.84 (0.17)	4.41 (0.04)*	0.69 (0.40)	0.26 (0.61)	0.01 (1.00)
	Public tap/standpipe	28.68 (0.01)*	1.32 (0.25)	0.03 (0.86)	0.56 (0.45)	0.22 (0.64)	0.26 (0.61)
	Compound well	26.55 (0.01)*	0.64 (0.42)	0.55 (0.46)	0.01 (0.98)	2.23 (0.14)	1.53 (0.22)
	Spring water	73.54 (0.01)*	0.41 (0.52)	0.08 (0.78)	11.40 (0.01)*	0.28 (0.60)	4.01 (0.04)*
	Rainwater	31.88 (0.01)*	6.70 (0.01)*	0.19 (0.66)	26.21 (0.01)*	3.17 (0.07)	34.66 (0.01)*
	Bowser water	0.01 (0.99)	7.23 (0.01)*	1.56 (0.21)	0.01 (1.00)	2.82 (0.09)	0.06 (0.80)
	Kiosk water	1.16 (0.28)	0.04 (0.84)	9.64 (0.01)*	0.04 (0.84)	3.42 (0.06)	27.26 (0.01)*
	Bottled water	0.00 (1.00)	7.95 (0.01)*	0.82 (0.36)	0.53 (0.47)	0.71 (0.40)	4.00 (0.04)*
	Sachet water	9.53 (0.02)*	24.37 (0.01)	18.28 (0.01)*	54.75 (0.01)*	12.55 (0.01)*	15.95 (0.01)*
	Surface water	0.00 (1.00)	2.24 (0.13)	14.93 (0.01)*	0.01 (0.94)	1.99 (0.16)	17.74 (0.01)*
	Neighbour’s well	2.35 (0.13)	1.85 (0.17)	0.14 (0.71)	32.20 (0.01)*	1.05 (0.30)	2.54 (0.11)
	Other water sources	1.27 (0.26)	0.01 (1.00)	0.01 (1.00)	7.89 (0.01)*	9.68 (0.01)*	0.01 (1.00)
Water distance		29.03 (0.01)*	2.11 (0.55)	49.35 (0.01)*	18.56 (0.01)*	5.64 (0.13)	24.56 (0.01)*
Water shortage		6.55 (0.01)*	5.57 (0.02)*	3.79 (0.05)	3.49 (0.06)	4.02 (0.04)*	0.03 (0.86)
Toilet used	Flush	24.32 (0.01)*	4.13 (0.04)*	0.13 (0.72)	7.10 (0.01)*	1.35 (0.24)	13.58 (0.01)*
	Latrine	8.87 (0.01)*	2.13 (0.14)	0.04 (0.84)	1.12 (0.29)	2.63 (0.11)	0.38 (0.54)
	Bucket	17.88 (0.01)*	2.24 (0.13)	29.92 (0.01)*	0.53 (0.47)	2.62 (0.11)	8.54 (0.01)*
	Hanging	2.17 (0.14)	0.10 (0.75)	0.14 (0.71)	2.56 (0.11)	0.07 (0.79)	0.02 (0.88)
	Flying	1.58 (0.21)	1.21 (0.27)	0.06 (0.80)	0.01 (0.95)	1.46 (0.23)	0.01 (1.00)*
	Open defecation	5.33 (0.02)*	0.99 (0.32)	0.04 (0.85)	15.86 (0.01)*	0.01 (1.00)	0.37 (0.54)
	Other	0.22 (0.63)	0.01 (0.93)	0.01 (0.95)	2.58 (0.11)	0.01 (1.00)	0.01 (1.00)
Shared toilet		6.62 (0.01)*	7.52 (0.01)*	6.33 (0.01)*	0.41 (0.52)	3.26 (0.07)	0.64 (0.42)
Toilet access		1.00 (0.32)	3.23 (0.07)	12.62 (0.01)*	7.21 (0.01)*	9.12 (0.01)*	8.14 (0.01)
Waste disposal areas	Community	0.01 (1.00)	1.13 (0.29)	1.50 (0.22)	1.98 (0.16)	2.10 (0.14)	9.74 (0.01)*
	Around house	0.01 (1.00)	2.26 (0.13)	0.02 (0.89)	2.90 (0.09)	0.02 (0.89)	0.02 (0.88)
	Dumping site	0.05 (0.83)	0.20 (0.66)	0.07 (0.80)	0.01 (0.91)	3.71 (0.05)	8.63 (0.01)*
	Drainage	8.73 (0.01)*	6.26 (0.01)*	8.35 (0.01)*	1.26 (0.26)	0.02 (0.89)	0.25 (0.62)
	Solid waste collectors	0.01 (1.00)	7.50 (0.01)*	6.81 (0.01)*	0.01 (1.00)	0.20 (0.65)	0.01 (0.93)
	Waste sea	0.01 (1.00)	0.49 (0.49)	8.99 (0.01)*	0.70 (0.40)	0.72 (0.39)	0.06 (0.81)
	Waste others	1.58 (0.21)	5.20 (0.02)*	2.32 (0.12)	0.43 (0.51)	0.44 (0.51)	17.77 (0.01)*
Waste payment		1.53 (0.22)	4.44 (0.04)*	6.56 (0.01)*	0.86 (0.35)	0.01 (0.96)	2.51 (0.11)
Environmental disaster		0.32 (0.57)	2.57 (0.11)	24.91 (0.01)*	1.81 (0.18)	0.08 (0.78)	15.60 (0.01)*
Enabling factors							
Income generating activity		14.28 (0.01)*	0.01 (0.99)	4.58 (0.03)*	23.44 (0.01)*	1.98 (0.16)	0.13 (0.71)
Household tenure		3.25 (0.35)	3.36 (0.34)	3.30 (0.35)	1.82 (0.61)	5.81 (0.12)	0.99 (0.80)
Sources of household income	Business	2.69 (0.10)	0.02 (0.90)	0.06 (0.81)	7.27 (0.01)*	0.01 (0.95)	2.05 (0.15)
	Fishing	0.01 (1.00)	0.11 (0.74)	0.01 (1.00)	1.85 (0.17)	0.01 (1.00)	0.44 (0.51)
	Government salaried	1.68 (0.20)	1.50 (0.22)	0.29 (0.59)	0.49 (0.48)	0.01 (1.00)	2.15 (0.14)
	Private salaried	0.56 (0.45)	0.11 (0.74)	9.47 (0.01)*	0.03 (0.86)	3.02 (0.08)	0.02 (0.89)
	Informal salaried	0.01 (1.00)	1.16 (0.28)	3.18 (0.07)	0.01 (1.00)	0.43 (0.51)	12.84 (0.01)*
	Daily wage	16.03 (0.01)*	0.41 (0.52)	5.04 (0.02)*	0.01 (1.00)	2.64 (0.10)	0.01 (0.94)
	Bike ride	0.80 (0.37)	†	1.25 (0.26)	1.00 (0.33)	0.18 (0.67)	0.36 (0.55)
	Stone mine	0.01 (1.00)	†	0.28 (0.60)	0.01 (1.00)	†	4.52 (0.03)*
	Unemployed	0.01 (1.00)	1.77 (0.18)	1.30 (0.25)	0.02 (0.88)	0.26 (0.61)	0.09 (0.77)
	Others	0.08 (0.78)	4.74 (0.03)*	1.63 (0.20)	10.43 (0.01)*	0.75 (0.38)	1.89 (0.17)

* Statistically significant at 95%.

† Excluded due to small size in a category.

Regarding toilet type, significant associations were observed for HU within Cockle Bay with flush, latrine, bucket toilets and open defecation. Bucket toilet use was also significantly associated with HU within the settlement in Moyiba. For HU outside settlement, flush toilets and open defecation were significant in Cockle Bay, while flush and bucket toilets were significant in Moyiba. None of the toilet types were significantly associated with HU in Dwarzark, for either within settlement or outside settlement contexts.

Toilet access was significantly associated with HU within settlements only in Moyiba, but for HU outside settlement, it was significant across all settlements. Waste disposal via drainage systems was significantly associated with HU within settlements across all settlements. Solid waste disposal was significant in Dwarzark and Moyiba. For HU outside settlement, waste disposal in community spaces, dumping sites and other locations was significantly associated with HU only in Moyiba. No significant associations were found in Cockle Bay and Dwarzark.

Environmental disasters were significantly associated with HU both within and outside settlements only in Moyiba. Daily wages were significantly associated with HU within settlements in Cockle Bay and Moyiba. For HU outside settlements, informal salaried work and stone mining were significantly associated in Moyiba, while other income sources were significant in Cockle Bay.

In summary, HU in Freetown’s informal settlements is influenced by household-level predisposing and enabling factors, but the associations vary by settlement and by whether care is sought within or outside the settlement. Additionally, descriptive statistics, including frequencies and percentages of HU both within and outside informal settlements, are in online supplemental material tables 1,2, respectively.

Table 2 presents RR and 95% CIs, examining the association between predisposing and enabling household-level explanatory factors and HU within informal settlements across the three settlements. Male-headed households in Dwarzark were 7% more likely to use healthcare within the settlement compared with female-headed households. In contrast, in Moyiba, male-headed households were 4% less likely to use HU within the settlement, while no meaningful difference was observed in Cockle Bay. In Dwarzark and Moyiba, households with a disabled person were 13% and 10% more likely, respectively, to seek HU within settlements. Food-insecure households in Dwarzark were 9% less likely to seek healthcare than those without food insecurity, whereas in Moyiba, they were 2% more likely. Households in Cockle Bay and Moyiba that took more than 30 min to access water were between 6% and 16% more likely to use HU within settlements, whereas water access time was not a significant factor in Dwarzark. In Cockle Bay, households using public taps, rainwater, bowser water and piped dwelling water were more likely to use HU within the settlement. In contrast, those using water from a piped neighbour and a neighbour’s well were less likely. In Dwarzark, using rainwater, surface water and other water sources increased the likelihood of HU within the settlement, while using a piped neighbour’s supply, rainwater and a neighbour’s well was associated with reduced HU within settlement. In Moyiba, households using bottled water, surface water and other sources were more likely to use HU within the settlement, whereas those relying on rainwater, a neighbour’s well or piped dwelling water were less likely to do so.

**Table 2:** Estimates of relative risk (RR) and 95% CIs for healthcare utilisation within informal settlement in Cockle Bay, Dwarzark and Moyiba

Variable		Categories (reference)	Cockle Bay	Dwarzark	Moyiba
			RR (95% CI)	RR (95% CI)	RR (95% CI)
Predisposing factors					
Intercept			0.64 (0.57 to 0.71)	0.52 (0.45 to 0.61)	0.57 (0.53 to 0.62)
Head of household gender		Female (ref)	1.00	1.00	1.00
		Male	0.98 (0.95 to 1.02)	1.07 (1.02 to 1.12)	0.96 (0.94 to 0.99)
Disability in household		No (ref)	1.00	1.00	1.00
		Yes	1.01 (0.94 to 1.08)	1.13 (1.04 to 1.24)	1.10 (1.06 to 1.14)
Family type		Single (ref)	1.00	1.00	1.00
		Married/cohabit/engaged	1.00 (0.96 to 1.03)	1.01 (0.97 to 1.05)	0.97 (0.95 to 1.00)
		Divorced/separated/widowed	1.07 (1.02 to 1.12)	0.85 (0.79 to 0.91)	1.02 (0.98 to 1.06)
Food security		Food secure (ref)	1.00	1.00	1.00
		Food insecure	0.97 (0.94 to 1.01)	0.91 (0.87 to 0.94)	1.02 (1.00 to 1.05)
Length of residence		0–1 years (ref)	1.00	1.00	1.00
		1–5 years	1.00 (0.96 to 1.03)	0.99 (0.93 to 1.06)	1.01 (0.98 to 1.06)
		6–10 years	0.99 (0.95 to 1.03)	0.99 (0.91 to 1.07)	1.01 (0.97 to 1.05)
		>10 years	0.98 (0.94 to 1.03)	0.98 (0.92 to 1.05)	0.93 (0.89 to 0.97)
Water sources	Piped dwelling	No (ref)	1.00	1.00	1.00
		Yes	1.07 (1.02 to 1.12)	1.16 (1.06 to 1.26)	1.01 (0.98 to 1.05)
	Piped neighbour	No (ref)	1.00	1.00	1.00
		Yes	0.77 (0.72 to 0.82)	0.92 (0.76 to 1.12)	0.86 (0.80 to 0.93)
	Public tap/standpipe	No (ref)	1.00	1.00	1.00
		Yes	1.21 (1.17 to 1.25)	0.95 (0.90 to 1.00)	1.01 (0.99 to 1.04)
	Rainwater	No (ref)	1.00	1.00	1.00
		Yes	1.08 (1.05 to 1.12)	1.21 (1.16 to 1.26)	0.94 (0.92 to 0.96)
	Bowser water	No (ref)	1.00	1.00	1.00
		Yes	1.63 (1.28 to 2.09)	1.14 (1.07 to 1.21)	0.95 (0.84 to 1.09)
	Kiosk water	No (ref)	1.00	1.00	1.00
		Yes	0.92 (0.78 to 1.09)	0.95 (0.84 to 1.07)	1.05 (1.02 to 1.08)
	Bottled water	No (ref)	1.00	1.00	1.00
		Yes	1.13 (0.99 to 1.3)	1.11 (1.04 to 1.19)	1.10 (1.04 to 1.17)
	Sachet water	No (ref)	1.00	1.00	1.00
		Yes	1.00 (0.96 to 1.03)	1.21 (1.16 to 1.27)	1.04 (1.02 to 1.06)
	Water surface	No (ref)	1.00	1.00	1.00
		Yes	1.01 (0.85 to 1.20)	1.22 (1.17 to 1.28)	1.12 (1.09 to 1.15)
	Neighbours’ well	No (ref)	1.00	1.00	1.00
		Yes	0.93 (0.89 to 0.98)	0.90 (0.86 to 0.95)	0.93 (0.89 to 0.96)
	Other water sources	No (ref)	1.00	1.00	1.00
		Yes	1.02 (0.95 to 1.10)	1.41 (1.24 to 1.62)	1.47 (1.28 to 1.68)
Water distance		Less than 30 min (ref)	1.00	1.00	1.00
		30 min −1 hour	1.16 (1.12 to 1.20)	0.98 (0.94 to 1.03)	1.06 (1.02 to 1.09)
		1–2 hours	1.16 (1.05 to 1.28)	0.89 (0.83 to 0.95)	1.15 (1.11 to 1.20)
		Over 2 hours	1.16 (1.04 to 1.29)	1.01 (0.96 to 1.07)	1.11 (1.07 to 1.15)
Water shortage		No (ref)	1.00	1.00	1.00
		Yes	1.06 (1.02 to 1.10)	1.15 (1.11 to 1.20)	0.97 (0.95 to 0.99)
Toilet types	Flush	No (ref)	1.00	1.00	1.00
		Yes	0.95 (0.9 to 1.00)	0.92 (0.85 to 1.00)	1.04 (1.00 to 1.09)
	Latrine	No (ref)	1.00	1.00	1.00
		Yes	1.06 (1.01 to 1.11)	1.00 (0.93 to 1.08)	1.00 (0.96 to 1.05)
	Bucket	No (ref)	1.00	1.00	1.00
		Yes	1.07 (1.02 to 1.12)	0.91 (0.85 to 0.97)	1.07 (1.05 to 1.10)
	Open defecation	No (ref)	1.00	1.00	1.00
		Yes	0.80 (0.73 to 0.88)	0.67 (0.40 to 1.14)	0.93 (0.85 to 1.01)
Shared toilet		No (ref)	1.00	1.00	1.00
		Yes	1.05 (1.01 to 1.08)	1.14 (1.09 to 1.19)	1.04 (1.02 to 1.06)
Waste disposal	Around house	No (ref)	1.00	1.00	1.00
		Yes	1.01 (0.85 to 1.22)	0.78 (0.75 to 0.83)	1.04 (1.01 to 1.07)
	Dumping site	No (ref)	1.00	1.00	1.00
		Yes	0.98 (0.86 to 1.12)	0.77 (0.70 to 0.84)	1.03 (0.99 to 1.06)
	Drainage	No (ref)	1.00	1.00	1.00
		Yes	0.80 (0.74 to 0.85)	0.75 (0.7 to 0.81)	1.05 (1.03 to 1.08)
	Solid waste collectors	No (ref)	1.00	1.00	1.00
		Yes	1.30 (1.19 to 1.43)	0.89 (0.80 to 1.00)	1.05 (0.99 to 1.12)
	Waste others	No (ref)	1.00	1.00	1.00
		Yes	1.41 (1.31 to 1.52)	0.71 (0.65 to 0.77)	0.80 (0.66 to 0.95)
Waste payment		No (ref)	1.00	1.00	1.00
		Yes	0.81 (0.72 to 0.90)	1.00 (0.90 to 1.10)	1.03 (0.97 to 1.09)
Environmental disaster		No (ref)	1.00	1.00	1.00
		Yes	0.99 (0.84 to 1.16)	0.85 (0.79 to 0.92)	0.81 (0.76 to 0.86)
Enabling factors					
Income activity engagement		No (ref)	1.00	1.00	1.00
		Yes	1.06 (1.03 to 1.09)	1.06 (1.03 to 1.10)	1.03 (1.01 to 1.05)
Household tenure		Tenant (ref)	1.00	1.00	1.00
		Landlord	0.99 (0.96 to 1.03)	1.07 (1.03 to 1.11)	1.03 (1.01 to 1.06)
		Free living	0.93 (0.86 to 1.00)	1.00 (0.96 to 1.04)	1.00 (0.97 to 1.03)
		Caretaker/lease/temporary stay/others	0.98 (0.91 to 1.06)	0.93 (0.79 to 1.09)	1.00 (0.93 to 1.07)
Sources of income	Fishing	No (ref)	1.00	1.00	1.00
		Yes	1.06 (0.96 to 1.17)	1.50 (1.39 to 1.63)	1.01 (0.96 to 1.07)
	Private salaried	No (ref)	1.00	1.00	1.00
		Yes	0.99 (0.95 to 1.04)	0.98 (0.94 to 1.02)	0.87 (0.83 to 0.91)
	Informal salaried	No (ref)	1.00	1.00	1.00
		Yes	1.11 (0.81 to 1.5)	1.45 (1.33 to 1.58)	1.00 (0.96 to 1.05)
	Daily wage	No (ref)	1.00	1.00	1.00
		Yes	0.58 (0.48 to 0.71)	0.97 (0.91 to 1.03)	1.07 (1.05 to 1.10)
	Bike ride	No (ref)	1.00	1.00	1.00
		Yes	0.97 (0.87 to 1.08)	0.99 (0.91 to 1.08)	0.95 (0.92 to 0.98)
	Others	No (ref)	1.00	1.00	1.00
		Yes	1.03 (0.97 to 1.09)	0.86 (0.78 to 0.95)	0.99 (0.95 to 1.03)

min, minutes; RR, relative risk.

Households in Cockle Bay and Dwarzark that experienced water shortages were 6% and 15% more likely, respectively, to use healthcare within the settlement compared to those without shortages, while in Moyiba, there were 3% less likely to do so. Regarding sanitation, households in Cockle Bay and Moyiba that used bucket toilets were 7% likely to seek healthcare within settlement, whereas in Dwarzark, bucket toilet use was associated with a 9% lower likelihood. Households practising open defecation in Cockle Bay were 20% less likely to use healthcare services within the settlement, and toilet users in Dwarzark were similarly 9% less likely to do so. In contrast, the use of shared toilets was consistently associated with a higher likelihood of HU within all settlements, compared to households with private toilet access.

In Dwarzark, households that disposed of waste around their houses, at dumping sites, or through drainage were less likely to utilise healthcare within settlements. In contrast, in Moyiba, households that disposed of waste around their houses and through drainage were 4% and 5% more likely, respectively, to use healthcare within the settlement. In Cockle Bay, a significant increase in HU within the settlements was observed among households that used solid waste collectors and other unspecified disposal methods, with increases of 30% and 41%, respectively. However, households in Cockle Bay that disposed of waste via drainage were 20% less likely to seek healthcare, and those who paid for waste collection were 19% less likely to do so. No significant differences were observed in Dwarzark and Moyiba regarding paid waste collection. Households that experienced an environmental disaster were more likely to use healthcare within settlements in Dwarzark and Moyiba, with increases of 15% and 19%, respectively, while no meaningful change was observed in Cockle Bay.

Across all study areas, households engaged in income-generating activities were more likely to seek healthcare within settlements than those without. In Dwarzark and Moyiba, landlords were 7% and 3% more likely, respectively, to utilise HU within the settlement compared to tenants, with no significant differences observed in Cockle Bay. Regarding sources of income, households relying on daily wages in Cockle Bay were 42% less likely to utilise healthcare within the settlement. In Dwarzark, households engaged in informal salaried work and fishing were 45% and 50% more likely to seek healthcare within the settlement, while those involved in other types of activities were 14% less likely to do so. In Moyiba, households engaged in bike riding and private salaried employment were 5% and 13% less likely, respectively, to use healthcare within the settlement, while those relying on daily wages were 7% more likely to do so.

In summary, HU within informal settlements is influenced by household-level predisposing and enabling factors, but the strength and direction of these associations vary across settlements. Predicted probabilities of HU within informal settlements for each household-level factor (ie, AR) included in the model are provided in online supplemental table 4.

Table 3 presents the RR and 95% CI, examining associations between predisposing and enabling factors and household’s HU outside informal settlements across three settlements. In Cockle Bay, male-headed households were 10% more likely to use healthcare outside the settlement compared to female-headed households. In contrast, male-headed households in Moyiba were 9% less likely to utilise healthcare outside the settlements. The presence of a disabled household member was significantly associated with lower HU outside the settlements across all settlements: Cockle Bay (15%), Dwarzark (33%) and Moyiba (23%). Household marital status also influenced HU patterns outside settlements, with married or cohabiting individuals in Cockle Bay 14% more likely to use healthcare compared to singles, while divorced/separated/widowed households were 23% more likely. Conversely, married or cohabiting individuals were 10% less likely, and divorced/separated/widowed individuals were 40% less likely to use healthcare outside. In Moyiba, divorced/separated/widowed individuals were 17% more likely, whereas married/cohabiting/engaged households were 13% less likely.

**Table 3:** Estimates of relative risk (RR) and 95% CIs for healthcare utilisation outside informal settlement in Cockle Bay, Dwarzark and Moyiba

Variable		Categories (reference)	Cockle Bay	Dwarzark	Moyiba
			RR (95% CI)	RR (95% CI)	RR (95% CI)
Predisposing factors					
Intercept			0.46 (0.39 to 0.55)	0.51 (0.37 to 0.71)	0.24 (0.20 to 0.30)
Head of household gender		Female (ref)	1.00	1.00	1.00
		Male	1.10 (1.04 to 1.16)	0.94 (0.85 to 1.04)	0.91 (0.84 to 0.99)
Disability in household		No (ref)	1.00	1.00	1.00
		Yes	0.85 (0.81 to 0.90)	0.67 (0.61 to 0.74)	0.77 (0.72 to 0.83)
Family type		Single (ref)	1.00	1.00	1.00
		Married/cohabit/engaged	1.14 (1.08 to 1.20)	0.90 (0.82 to 0.99)	0.87 (0.81 to 0.94)
		Divorced/separated/widowed	1.23 (1.14 to 1.32)	0.60 (0.52 to 0.70)	1.17 (1.06 to 1.29)
Food security		Food secure (ref)	1.00	1.00	1.00
		Food insecure	0.93 (0.89 to 0.98)	1.00 (0.92 to 1.09)	2.74 (2.54 to 2.96)
Length of residence		0–1 years (ref)	1.00	1.00	1.00
		1–5 years	1.24 (1.11 to 1.38)	0.73 (0.61 to 0.86)	1.03 (0.90 to 1.19)
		6–10 years	1.22 (1.09 to 1.36)	0.82 (0.70 to 0.96)	1.13 (0.98 to 1.30)
		More than 10 years	1.28 (1.15 to 1.43)	0.86 (0.75 to 0.99)	0.99 (0.87 to 1.13)
Water sources	Piped neighbour	No (ref)	1.00	1.00	1.00
		Yes	1.03 (0.98 to 1.08)	1.27 (0.97 to 1.67)	0.86 (0.74 to 1.02)
	Public tap/standpipe	No (ref)	1.00	1.00	1.00
		Yes	1.03 (0.98 to 1.09)	1.15 (1.02 to 1.29)	0.91 (0.85 to 0.97)
	Compound well	No (ref)	1.00	1.00	1.00
		Yes	1.04 (0.95 to 1.14)	1.07 (0.97 to 1.17)	1.50 (1.37 to 1.64)
	Rainwater	No (ref)	1.00	1.00	1.00
		Yes	0.88 (0.82 to 0.93)	0.82 (0.76 to 0.9)	0.79 (0.72 to 0.86)
	Bowser water	No (ref)	1.00	1.00	1.00
		Yes	1.00 (0.81 to 1.23)	0.74 (0.59 to 0.93)	0.76 (0.52 to 1.12)
	Kiosk water	No (ref)	1.00	1.00	1.00
		Yes	0.99 (0.88 to 1.11)	0.44 (0.32 to 0.60)	1.39 (1.29 to 1.49)
	Bottled water	No (ref)	1.00	1.00	1.00
		Yes	1.34 (1.25 to 1.45)	1.79 (1.44 to 2.22)	1.51 (1.26 to 1.81)
	Sachet water	No (ref)	1.00	1.00	1.00
		Yes	1.22 (1.15 to 1.30)	0.74 (0.68 to 0.80)	1.18 (1.11 to 1.26)
	Water surface	No (ref)	1.00	1.00	1.00
		Yes	0.99 (0.72 to 1.38)	0.75 (0.65 to 0.87)	1.11 (1.01 to 1.23)
	Neighbours’ well	No (ref)	1.00	1.00	1.00
		Yes	1.19 (1.13 to 1.25)	0.83 (0.75 to 0.93)	1.00 (0.91 to 1.09)
	Other sources	No (ref)	1.00	1.00	
		Yes	0.82 (0.71 to 0.94)	1.84 (1.62 to 2.09)	
Water distance		Less 30 min (ref)	1.00	1.00	1.00
		30 min–1 hours	0.82 (0.78 to 0.87)	1.22 (1.09 to 1.36)	0.90 (0.82 to 0.99)
		1–2 hours	1.02 (0.95 to 1.11)	1.16 (1.02 to 1.31)	1.11 (1.01 to 1.21)
		Over 2 hours	1.07 (0.99 to 1.17)	1.17 (1.05 to 1.30)	0.94 (0.86 to 1.03)
Water shortage		No (ref)	1.00	1.00	1.00
		Yes	0.89 (0.85 to 0.93)	0.70 (0.63 to 0.78)	0.95 (0.89 to 1.02)
Toilet types	Flush	No (ref)	1.00	1.00	1.00
		Yes	1.13 (1.07 to 1.20)	1.09 (0.98 to 1.20)	1.30 (1.02 to 1.40)
	Hanging	No (ref)	1.00	1.00	1.00
		Yes	1.02 (0.96 to 1.07)	0.68 (0.38 to 1.20)	0.91 (0.76 to 1.10)
	Open defecation	No (ref)	1.00	1.00	1.00
		Yes	1.10 (1.03 to 1.17)	0.90 (0.71 to 1.14)	1.09 (0.79 to 1.49)
Shared toilet		No (ref)	1.00	1.00	1.00
		Yes	0.99 (0.94 to 1.03)	1.15 (1.05 to 1.26)	1.10 (1.04 to 1.17)
Toilet access		No (ref)	1.00	1.00	1.00
		Yes	1.08 (1.02 to 1.14)	0.73 (0.64 to 0.84)	1.04 (0.96 to 1.12)
Waste disposal areas	Community	No (ref)	1.00	1.00	1.00
		Yes	1.01 (0.93 to 1.10)	1.22 (0.98 to 1.52)	0.59 (0.52 to 0.66)
	Dumping site	No (ref)	1.00	1.00	1.00
		Yes	1.12 (0.96 to 1.30)	0.89 (0.75 to 1.06)	1.25 (1.16 to 1.35)
	Waste sea	No (ref)	1.00	1.00	1.00
		Yes	0.87 (0.81 to 0.93)	0.59 (0.35 to 1.00)	1.18 (0.91 to 1.54)
	Waste others	No (ref)	1.00	1.00	1.00
		Yes	1.14 (1.03 to 1.27)	1.27 (1.14 to 1.42)	1.73 (1.50 to 1.99)
Environmental disaster		No (ref)	1.00	1.00	1.00
		Yes	1.34 (1.23 to 1.47)	0.85 (0.75 to 0.96)	1.00 (0.90 to 1.12)
Enabling factors					
Income activity engagement		No (ref)	1.00	1.00	1.00
		Yes	0.88 (0.84 to 0.93)	0.88 (0.81 to 0.96)	1.04 (0.97 to 1.11)
Household tenure		Tenant (ref)	1.00	1.00	1.00
		Landlord	1.09 (1.05 to 1.14)	1.07 (0.98 to 1.17)	1.08 (1.00 to 1.15)
		Free living	1.15 (1.06 to 1.25)	0.94 (0.81 to 1.08)	1.06 (0.97 to 1.16)
		Caretaker/lease/temporary stay/others	1.07 (0.97 to 1.17)	0.92 (0.72 to 1.17)	1.43 (1.28 to 1.60)
Sources of household income	Business	No (ref)	1.00	1.00	1.00
		Yes	1.10 (1.05 to 1.15)	1.06 (0.98 to 1.14)	1.04 (0.98 to 1.10)
	Fishing	No (ref)	1.00	1.00	1.00
		Yes	1.18 (1.09 to 1.28)	1.29 (0.67 to 2.50)	2.25 (1.86 to 2.73)
	Government salaried	No (ref)	1.00	1.00	1.00
		Yes	1.04 (0.92 to 1.17)	0.94 (0.84 to 1.07)	1.01 (0.90 to 1.12)
	Informal salaried	No (ref)	1.00	1.00	1.00
		Yes	0.56 (0.17 to 1.88)	0.31 (0.11 to 0.93)	1.58 (1.46 to 1.71)
	Daily wage	No (ref)	*	1.00	1.00
		Yes	*	0.75 (0.63 to 0.90)	0.95 (0.87 to 1.04)
	Bike ride	No (ref)	1.00	1.00	1.00
		Yes	1.14 (1.05 to 1.25)	1.43 (1.17 to 1.75)	1.06 (0.98 to 1.14)
	Stone mine	No (ref)	1.00	*	1.00
		Yes	1.33 (0.99 to 1.79)	*	0.59 (0.52 to 0.68)
	Others	No (ref)	1.00	1.00	1.00
		Yes	1.18 (1.12 to 1.24)	1.19 (1.08 to 1.32)	0.98 (0.86 to 1.11)

* Excluded due to small sample size in a category.

min, minutes; RR, relative risk.

The length of residence was positively associated with HU outside Cockle Bay, with households residing for more than 1 year being at least 20% more likely to seek healthcare outside the settlement. In contrast, longer-term residence in Dwarzark was associated with lower healthcare outside the settlement, ranging from 3% to 18% less compared to recent arrivals. No meaningful associations were observed in Moyiba.

Water sources showed a strong association with HU outside settlements. In Dwarzark, households using public taps were 15% more likely to seek healthcare outside settlements, whereas in Moyiba, these households were 9% less likely. Compound wells were associated with a 50% increase in HU outside Moyiba. The use of rainwater was consistently associated with lower HU outside settlements, ranging from 12% in Cockle Bay to 21% in Moyiba. In contrast, the use of bottled water was positively associated with HU outside settlements across all study areas, with the strongest association in Dwarzark (79% more likely). Households using sachet water were 22% and 18% more likely to utilise healthcare outside the settlements in Cockle Bay and Moyiba, respectively, but were 26% less likely in Dwarzark. In Moyiba, households using other water sources were 84% more likely to use healthcare outside the settlements, whereas in Cockle Bay they were 18% less likely.

Regarding water access distance, in Dwarzark, households that travelled between 30 min and over 2 hours to fetch water were 16%–20% more likely to use healthcare outside the settlements. However, in Cockle Bay and Moyiba, the associations were less consistent. Water shortage was negatively associated with HU outside the settlement in Cockle Bay (11% less likely) and Dwarzark (30% less likely), while no significant difference was observed in Moyiba.

Household-level sanitation factors also influenced HU outside informal settlements. Households using flush toilets were associated with greater HU outside the settlements in Cockle Bay (13%) and Moyiba (30%), while no significant associations were observed for hanging toilets or open defecation across all settlements. Shared toilet use was associated with a higher likelihood of HU outside Dwarzark (15%) and Moyiba (10%) but showed no meaningful difference in Cockle Bay. Access to toilets was positively associated with HU outside the settlements in Cockle Bay (8%) but negatively associated in Dwarzark (27% less likely).

For waste disposal, households in Moyiba that disposed of waste in the community were 41% less likely to use healthcare outside the settlements, while those using dumping sites were 25% more likely. Waste disposal through other means was associated with higher HU outside the settlements across all study areas. Environmental disaster exposure was associated with a 34% increase in HU outside Cockle Bay, but a 15% decrease in Dwarzark.

In terms of household tenure, landlords in Cockle Bay and Moyiba were 9% and 8% more likely, respectively, to utilise healthcare outside the settlement compared to tenants. Additionally, rent-free households in Cockle Bay were 15% more likely, and households in Moyiba living under lease/temporary/caretakers were 43% more likely to use healthcare outside the settlement. No significant differences were observed in Dwarzark with respect to household tenure. Participation in income-generating activities was associated with a 12% lower likelihood of HU outside the settlements in both Cockle Bay and Dwarzark, with no significant association observed in Moyiba. Food insecurity was associated with a 7% reduction in HU outside Cockle Bay, but in Moyiba, it was strongly associated with a 174% increase, indicating substantial differences in vulnerability across study areas.

Finally, sources of household income significantly influenced patterns of HU outside the settlements. In Cockle Bay, households engaged in business, fishing, bike riding and other income-generating activities were more likely to seek healthcare outside informal settlements. In Dwarzark, those engaged in bike riding and other income sources were similarly more likely to use healthcare outside the settlement, whereas informally salaried households were 71% less likely. In Moyiba, households engaged in fishing and informal salaried work were significantly more likely to use healthcare outside the settlement, while those involved in stone mining were 41% less likely.

In summary, findings in table 3 indicate that household-level determinants associated with HU outside informal settlements are highly context-specific. Some factors, such as disability and water sources, show consistent associations across settlements, while others, such as sources of income, vary considerably by settlement. Predicted probabilities of HU outside informal settlements for each household-level factor (ie, AR) are provided in online supplemental table 5.

The estimates for HU combined within and outside the settlements (online supplemental table 6) revealed that predisposing and enabling household-level factors associated with HU across the settlements are highly context-specific. Notably, households with disabled members consistently reported lower HU within and outside settlements across all areas. Regarding marital status, married/cohabiting households were associated with higher HU within and outside settlements in Cockle Bay and Moyiba, but lower HU in Dwarzark. Longer residence in Cockle Bay was associated with HU, unlike other areas. Overall, the results for HU within and outside the settlements indicate varying location-specific social and economic dynamics influencing HU. The AR for HU within and outside the settlements is provided in online supplemental table 7.

## Discussion

In this study, we explored how household-level predisposing and enabling factors within Andersen Behavioural framework are associated with HU both within and outside informal settlements across three settlements (ie, Cockle Bay, Dwarzark and Moyiba) in Freetown, Sierra Leone. The analysis revealed context-specific associations between these factors and HU, both within and outside the informal settlements.

Predisposing factors such as male-headed households were more likely to seek care in Dwarzark (7% more within and 10% more outside the settlement), whereas in Moyiba, they were less likely to seek care both within (4% less) and outside (9% less), suggesting the existence of settlement-specific gendered dynamics of HU. Disability status in a household showed contrasting patterns: households with a disabled member were more likely to utilise HU within Dwarzark and Moyiba, possibly reflecting the increased health needs typically associated with disability, which often correlate with poor health outcomes and greater care requirements compared with households without disabled persons.^27^ However, these households were significantly less likely to seek healthcare outside their settlements, especially in Dwarzark (33% less), perhaps due to reliance on more accessible healthcare options within the settlement or limited access outside their settlements due to already strained resources.

The influence of food insecurity on HU also varied, with reduced HU within Dwarzark but a slight increase of 2% in Moyiba, which may reflect the dual role of food insecurity as both a driver of poor health and a proxy for lack of stable income; an important barrier to healthcare access.^28^ These findings should be interpreted with caution, as the analysis focused on only one of the nine common experiences of food insecurity.^15 29^ Similarly, the length of residence influenced HU in opposite directions: it was positively associated with HU outside Cockle Bay, supporting evidence that recent migrants often face barriers to access,^2^ and negatively outside Dwarzark, highlighting the dynamic nature of healthcare access in informal settlements.

Households with longer water-fetching times and those experiencing water shortages were more likely to use healthcare within settlements, especially in Cockle Bay and Dwarzark, possibly due to the increased burden of waterborne diseases.^30^ However, this association was less clear for HU outside the settlements. The use of clean water sources (ie, piped dwelling, bowser, surface, bottled) was associated with increased HU both within and outside settlements across study areas, potentially reflecting the greater financial capacity to afford healthcare.^30^ On the other hand, households relying on neighbour-supplied piped and well water, and rainwater had lower HU within settlements, possibly indicating challenges related to healthcare affordability.^30^ This is despite the potential for water contamination during fetching, transportation and storage, which could lead to water-related diseases and is expected to increase HU.^31 32^ A possible explanation is that households residing far from amenities, such as water sources, may live in more affordable housing due to financial constraints, which could negatively impact their ability to afford healthcare despite being at higher risk of illnesses.^33^

Sanitation and hygiene factors demonstrate consistent associations with HU both within and outside settlements across study areas. Shared toilets were positively associated with HU both within and outside settlements in Dwarzark and Moyiba, suggesting their role as a proxy for higher exposure to disease risks and communal health vulnerabilities. The use of flush toilets was similarly associated with increased HU outside Cockle Bay and Moyiba, indicating that improved sanitation may correspond with better healthcare seeking behaviour. This may also reflect greater financial capacity to afford better healthcare outside the settlement. In contrast, open defecation and bucket toilet use in Dwarzark were associated with reduced HU within the settlement, which may reflect affordability constraints that deter or delay timely care seeking.^34^ Notably, the presence of private toilets was associated with increased HU outside the settlement in Cockle Bay, a community generally characterised by limited formal healthcare services, reflecting the capacity to afford better healthcare outside the settlement.^15^ However, in Dwarzark, where local healthcare services are comparatively better and more accessible, private toilet use was associated with reduced HU outside the settlement, suggesting that better local service availability mitigates the need to seek care beyond the immediate area.

Waste disposal practices also influenced HU patterns with households that used solid waste collectors and alternative methods having a higher HU within settlements (eg, 30% and 41% in Cockle Bay), while community dumping was associated with lower HU outside settlement, especially in Moyiba (41%). This likely reflects households’ capacity to afford healthcare, as those using solid waste collectors are better equipped to pay for healthcare services compared to those relying on community dumping. Environmental disasters were associated with reduced HU within settlements in Dwarzark and Moyiba, both of which are hilly areas where disasters disrupt already fragile healthcare systems, impeding timely access to care.^35^ However, the association between environmental disasters and HU outside settlements was mixed. In Cockle Bay, disasters were associated with increased HU, possibly due to a rise in illnesses, especially cholera, as it is a low-lying area combined with the absence of sufficient local healthcare infrastructure, pushing households to seek care elsewhere. In contrast, in Dwarzark, external HU decreased, potentially reflecting adequate within-settlement response capacity during such events.

To summarise, household-level predisposing factors showed that patterns of associations with HU both within and outside settlements varied, and that households most exposed to illnesses are not necessarily those most likely to use healthcare. This highlights the need for settlement-specific interventions, such as improving service access and affordability, while accounting for these differences.

For enabling factors, landlord households in Dwarzark and Moyiba were associated with increased HU within settlements, while those in Cockle Bay showed increased HU outside the settlement. This is likely due to greater capacity to afford healthcare through access to financial resources, enabling landlords in Cockle Bay to seek care outside their settlement, where better facilities are available. This indicates that the capacity to afford healthcare and access to financial resources influence healthcare-seeking behaviour in informal settlements, with well-resourced households leveraging their means to seek care more frequently and of high quality.

Households engaged in economic activities had consistently higher HU within settlements, offering clear insight into the role that higher income, employment and asset ownership play in promoting access.^2^ Income-generating households in Dwarzark were less likely to seek care outside, perhaps due to reliance on better and more accessible healthcare options within their settlement. Similarly, households in Cockle Bay engaged in income-generating activities were less likely to seek healthcare outside. This was surprising, given that Cockle Bay lacks formal health facilities, and the expectation was that households engaged in income-generating activities would have a better financial position to seek higher quality healthcare outside the settlement; similar to Cockle Bay landlord households, which, with greater resources, are more able to access care outside.

Finally, households engaged in fishing, informal salaried work and bike riding generally had higher HU both within and outside settlements, especially in Dwarzark and Moyiba, while those involved in stone mining in Moyiba and daily wage labourers in Cockle Bay were significantly less likely to seek healthcare within settlements. Daily wage labourers in Dwarzark were also less likely to seek healthcare outside settlement. These results underscore that sources of income and their level of stability influence HU both within and outside settlements. In summary, the findings from household-level enabling factors revealed settlement-specific patterns in HU, both within and outside informal settlements, highlighting the need for interventions aimed at strengthening local service provision, addressing affordability barriers and targeting vulnerable income groups such as daily wage earners.

One of the main strengths of this paper is that it provides valuable insights into household-level predisposing and enabling determinants associated with HU, both within and outside informal settlements in Freetown. The results highlight settlement-specific contrasts and unexpected associations, underscoring the need for universal yet targeted interventions based on level of vulnerability. This analysis represents a step forward in understanding the extent to which household-level factors influence HU in informal settlements and paves the way for further research on how these determinants intersect to amplify inequalities and inform strategies for addressing barriers in HU.

However, our analysis has some limitations. First, we only considered HU at household level, as the data were collected at this level. Second, our data were limited to three informal settlements, which may affect the generalisability of our findings to more than 60 informal urban settlements, given their heterogeneity as demonstrated in this paper. Third, only households with at least one adult (aged 18 years or older) were interviewed, which may have excluded child-headed households (under 18 years). Fourth, while our analyses focused on RR, which explain associations between household-level predisposing and enabling factors and HU, the policy relevance of these findings could be enhanced by also considering AR. For example, being married, cohabiting or engaged showed similar relative associations with HU within settlements, but its higher prevalence in Moyiba (0.4) compared with Dwarzark (0.3) implies a greater absolute burden. Although this study primarily focused on associations, we have provided AR in online supplemental material file 1 for readers interested in understanding which household-level areas are of greatest importance in specific settlements.

In conclusion, we have identified household-level predisposing and enabling factors associated with HU, both within and outside three informal settlements in Freetown, Sierra Leone. These findings will be instrumental in informing settlement-specific policies aimed at improving HU by addressing barriers to HU in informal settlements.

## Supplementary material

10.1136/bmjopen-2025-108022 online supplemental file 1

## Data Availability

Data are available on reasonable request.
