# Supplementary material for: Household determinants of healthcare utilisation in three informal settlements in Freetown, Sierra Leone: a cross-sectional survey
Source: BMJ Open. 2026 Mar 3;16(3):e108022. doi: 10.1136/bmjopen-2025-108022 (PMC12958868; doi:10.1136/bmjopen-2025-108022)
Supplement: online supplemental file 1 [file bmjopen-16-3-s001.docx]

**Supplementary material: The household determinants of healthcare utilisation in three informal settlements in Freetown, Sierra Leone. A cross-sectional study.**

Table S1: Definition of variables

| Variable | Definition |
| --- | --- |
| **Predisposing factors** | |
| Head of household gender gender |  |
| Disability in household | Limitations to a person’s ability to function in one or more ways |
| Family type |  |
| Single | Unmarried or not involved in a stable relationship |
| Married/cohabiting/engaged | Legally and socially sanctioned union including cohabiting and engaged |
| Divorced/Separated/widowed | No longer married or marriage legally dissolved or having lost a husband or wife through death |
| Tenant | Households who pay house rent to the landlords |
| Landlord | People who own the house they live in |
| Free-living | They neither pay rent or own the house |
| Others | Caretaker, lease, temporal stay |
| Food security |  |
| Food secure | Any household member able to eat the kinds of food they preferred due to affordability of resources |
| Food insecure | Any household member is not able to eat the kinds of food the preferred due to a lack of resources |
| Length of Stay | How long household members have stayed within the community |
| Water source |  |
| Kiosk/bowsers | Large water containers in strategic areas, protected and managed by community members and sometimes paid for by users |
| bottled | Private water vendors in bottles for high income users |
| sachet | Private water vendors in plastic packages for low-income users |
| surface | Water that licks out anywhere from the ground below and most times not protected |
| other | Stream, borehole |
| Toilet type |  |
| Flush | A more improved/advanced toilet facility for proper sanitation |
| Latrine | A simple hole/trench in the ground |
| Bucket | Small portable buckets for faeces |
| Hanging | Form of toilets built over water body |
| Flying | Defecation in plastic bags and then thrown |
| Open defecation | Practice of defecating outside like bushes, canals streams |
| other | Public toilets, plastic bags, in the sea |
| Waste disposal |  |
| Around house | Just throw waste somewhere/anywhere around the house/compound |
| Dumping site | In a designated waste area |
| Solid collectors | Individuals or institutions paid for collecting waste in houses |
| other | burning |
| **Enabling Factors** | |
| Income activity engagement | Engaged in any form of income activity like business |
| Household tenure |  |
| **Sources of Household income** |  |
| Business | Households in any form of buying and selling as a source of income. |
| Government salaried | Households in any government paid jobs. |
| Private salaried | Households in any form of privately paid jobs. |
| Informal salaried | Households engaged in not formal paid jobs e.g. nanny, cleaner sex workers |
| Daily wage | Laborers earning on a daily |
| Bike riders | Drivers of motorbikes as transportation in hard-to-reach areas |
| Stone mine | Individuals who manually mine/crush stone and sell them |
| Unemployed | No jobs that provides income |
| Others | Babysitter, farmer, footballer, hairdresser, plumber, tailoring etc |

Table S2: Frequencies and percentages of health utilisation within informal settlement

| Variable name | | Category | **Cockle Bay** | | | **Dwazark** | | | **Moyiba** | | |
| --- | --- | --- | --- | --- | --- | --- | --- | --- | --- | --- | --- |
|  |  |  | Frequency (%) | | | Frequency (%) | | | Frequency (%) | | |
|  |  |  | Yes | No | Total | No | Yes | Total | No | Yes | Total |
| **Predisposing factors** | | | | | | | | | | | |
| Head of household gender | | Male | 215 (27.1%) | 578 (72.9%) | 793 (72.6%) | 409 (45.3%) | 493 (54.7%) | 902 (71.0%) | 349 (21.2%) | 1294 (78.8%) | 1643 (73.3%) |
|  |  | Female | 64 (21.4%) | 235 (78.6%) | 299 (27.4%) | 170 (46.2%) | 198 (53.8%) | 368 (29.0%) | 155 (26.0%) | 442 (74.0%) | 597 (26.7%) |
| Disability in household | | Yes | 17 (31.5%) | 37 (68.5%) | 54 (4.9%) | 55 (54.5%) | 46 (45.5%) | 101 (8.0%) | 38 (26.4%) | 106 (73.6%) | 144 (6.4%) |
|  |  | No | 262 (25.2%) | 776 (74.8%) | 1038 (95.1%) | 524 (44.8%) | 645 (55.2%) | 1169 (92.0%) | 466 (22.2%) | 1630 (77.8%) | 2096 (93.6%) |
| Family type | | Single | 79 (22.1%) | 278 (77.9%) | 357 (32.7%) | 126 (42.9%) | 168 (57.1%) | 294 (23.1%) | 99 (19.4%) | 410 (80.6%) | 509 (22.7%) |
|  |  | Married, cohabit, engaged | 183 (28.5%) | 458 (71.5%) | 641 (58.7%) | 355 (45.8%) | 420 (54.2%) | 775 (61.0%) | 351 (23.4%) | 1146 (76.6%) | 1497 (66.8%) |
|  |  | Divorced, separated, widowed | 17 (18.1%) | 77 (81.9%) | 94 (8.6%) | 98 (48.8%) | 103 (51.2%) | 201 (15.8%) | 54 (23.1%) | 180 (76.9%) | 234 (10.4%) |
| Income generating activity | | Yes | 229 (28.6%) | 571 (71.4%) | 800 (73.3%) | 433 (45.5%) | 518 (54.5%) | 951 (74.9%) | 406 (23.6%) | 1317 (76.4%) | 1723 (76.9%) |
|  |  | No | 50 (17.1%) | 242 (82.9%) | 292 (26.7%) | 146 (45.8%) | 173 (54.2%) | 319 (25.1%) | 98 (19.0%) | 419 (81.0%) | 517 (23.1%) |
| Food security | | Food secure | 125 (21.5%) | 456 (78.5%) | 581 (53.2%) | 189 (40.6%) | 277 (59.4%) | 466 (36.7%) | 293 (22.7%) | 1000 (77.3%) | 1293 (57.7%) |
|  |  | Food insecure | 154 (30.1%) | 357 (69.9%) | 511 (46.8%) | 390 (48.5%) | 414 (51.5%) | 804 (63.3%) | 211 (22.3%) | 736 (77.7%) | 947 (42.3%) |
| Length of residence | | 0-1 years | 20 (20.0%) | 80 (80.0%) | 100 (9.2%) | 39 (42.9%) | 52 (57.1%) | 91 (7.2%) | 28 (19.2%) | 118 (80.8%) | 146 (6.5%) |
|  |  | 1-5 years | 91 (23.8%) | 291 (76.2%) | 382 (35.0%) | 121 (45.7%) | 144 (54.3%) | 265 (20.9%) | 129 (17.8%) | 596 (82.2%) | 725 (32.4%) |
|  |  | 6-10 years | 58 (25.6%) | 169 (74.4%) | 227 (20.8%) | 92 (46.7%) | 105 (53.3%) | 197 (15.5%) | 101 (20.4%) | 394 (79.6%) | 495 (22.1%) |
|  |  | More than 10 years | 110 (28.7%) | 273 (71.3%) | 383 (35.1%) | 327 (45.6%) | 390 (54.4%) | 717 (56.5%) | 246 (28.1%) | 628 (71.9%) | 874 (39.0%) |
| Household tenure | | Tenant | 194 (27.25%) | 518 (72.75%) | 712 (65.20%) | 340 (46.90%) | 385 (53.10%) | 725 (57.09%) | 286 (23.77%) | 917 (76.23%) | 1203 (53.71%) |
|  |  | landlord | 63 (22.03%) | 223 (77.97%) | 286 (26.19%) | 159 (41.84%) | 221 (58.16%) | 380 (29.92%) | 155 (21.77%) | 557 (78.23%) | 712 (31.79%) |
|  |  | Free living | 13 (24.53%) | 40 (75.47%) | 53 (4.85%) | 62 (47.69%) | 68 (52.31%) | 130 (10.24%) | 54 (19.78%) | 219 (80.22%) | 273 (12.19%) |
|  |  | Caretaker, lease, temporary stay, others | 9 (21.95%) | 32 (78.05%) | 41 (3.75%) | 18 (51.43%) | 17 (48.57%) | 35 (2.76%) | 9 (17.31%) | 43 (82.69%) | 52 (2.32%) |
| Water Source | Piped Dwelling | No | 166 (19.7%) | 678 (80.3%) | 844 (77.3%) | 568 (45.9%) | 669 (54.1%) | 1237 (97.4%) | 493 (22.5%) | 1694 (77.5%) | 2187 (97.6%) |
|  |  | Yes | 113 (45.6%) | 135 (54.4%) | 248 (22.7%) | 11 (33.3%) | 22 (66.7%) | 33 (2.6%) | 11 (20.8%) | 42 (79.2%) | 53 (2.4%) |
|  | Piped neighbor | No | 166 (19.7%) | 678 (80.3%) | 844 (77.3%) | 570 (45.5%) | 682 (54.5%) | 1252 (98.6%) | 478 (22.0%) | 1692 (78.0%) | 2170 (96.9%) |
|  |  | Yes | 113 (45.6%) | 135 (54.4%) | 248 (22.7%) | 9 (50.0%) | 9 (50.0%) | 18 (1.4%) | 26 (37.1%) | 44 (62.9%) | 70 (3.1%) |
|  | piped compound | No | 252 (24.5%) | 775 (75.5%) | 1027 (94.0%) | 573 (45.9%) | 676 (54.1%) | 1249 (98.3%) | 489 (22.2%) | 1711 (77.8%) | 2200 (98.2%) |
|  |  | Yes | 27 (41.5%) | 38 (58.5%) | 65 (6.0%) | 6 (28.6%) | 15 (71.4%) | 21 (1.7%) | 15 (37.5%) | 25 (62.5%) | 40 (1.8%) |
|  | Public tap/standpipe | No | 252 (29.3%) | 609 (70.7%) | 861 (78.8%) | 504 (45.0%) | 617 (55.0%) | 1121 (88.3%) | 356 (22.6%) | 1217 (77.4%) | 1573 (70.2%) |
|  |  | Yes | 27 (11.7%) | 204 (88.3%) | 231 (21.2%) | 75 (50.3%) | 74 (49.7%) | 149 (11.7%) | 148 (22.2%) | 519 (77.8%) | 667 (29.8%) |
|  | Rainwater | No | 235 (30.4%) | 538 (69.6%) | 773 (70.8%) | 328 (49.1%) | 340 (50.9%) | 668 (52.6%) | 344 (22.2%) | 1205 (77.8%) | 1549 (69.2%) |
|  |  | Yes | 44 (13.8%) | 275 (86.2%) | 319 (29.2%) | 251 (41.7%) | 351 (58.3%) | 602 (47.4%) | 160 (23.2%) | 531 (76.8%) | 691 (30.8%) |
|  | Bowser water | No | 279 (25.6%) | 811 (74.4%) | 1090 (99.8%) | 560 (46.5%) | 644 (53.5%) | 1204 (94.8%) | 499 (22.4%) | 1729 (77.6%) | 2228 (99.5%) |
|  |  | Yes | 0 (0%) | 2 (100.0%) | 2 (0.2%) | 19 (28.8%) | 47 (71.2%) | 66 (5.2%) | 5 (41.7%) | 7 (58.3%) | 12 (0.5%) |
|  | Kiosk water | No | 271 (25.3%) | 800 (74.7%) | 1071 (98.1%) | 555 (45.5%) | 665 (54.5%) | 1220 (96.1%) | 477 (23.4%) | 1563 (76.6%) | 2040 (91.1%) |
|  |  | Yes | 8 (38.1%) | 13 (61.9%) | 21 (1.9%) | 24 (48.0%) | 26 (52.0%) | 50 (3.9%) | 27 (13.5%) | 173 (86.5%) | 200 (8.9%) |
|  | Bottled water | No | 276 (25.5%) | 805 (74.5%) | 1081 (99.0%) | 576 (46.2%) | 672 (53.8%) | 1248 (98.3%) | 500 (22.6%) | 1711 (77.4%) | 2211 (98.7%) |
|  |  | Yes | 3 (27.3%) | 8 (72.7%) | 11 (1.0%) | 3 (13.6%) | 19 (86.4%) | 22 (1.7%) | 4 (13.8%) | 25 (86.2%) | 29 (1.3%) |
|  | Sachet water | No | 61 (19.1%) | 259 (80.9%) | 320 (29.3%) | 337 (52.5%) | 305 (47.5%) | 642 (50.6%) | 262 (26.8%) | 714 (73.2%) | 976 (43.6%) |
|  |  | Yes | 218 (28.2%) | 554 (71.8%) | 772 (70.7%) | 242 (38.5%) | 386 (61.5%) | 628 (49.4%) | 242 (19.1%) | 1022 (80.9%) | 1264 (56.4%) |
|  | Surface water | No | 277 (25.6%) | 807 (74.4%) | 1084 (99.3%) | 530 (46.3%) | 614 (53.7%) | 1144 (90.1%) | 450 (24.0%) | 1422 (76.0%) | 1872 (83.6%) |
|  |  | Yes | 2 (25.0%) | 6 (75.0%) | 8 (0.7%) | 49 (38.9%) | 77 (61.1%) | 126 (9.9%) | 54 (14.7%) | 314 (85.3%) | 368 (16.4%) |
|  | Neighbor’s well | No | 204 (24.4%) | 633 (75.6%) | 837 (76.6%) | 462 (44.6%) | 573 (55.4%) | 1035 (81.5%) | 450 (22.4%) | 1562 (77.6%) | 2012 (89.8%) |
|  |  | Yes | 75 (29.4%) | 180 (70.6%) | 255 (23.4%) | 117 (49.8%) | 118 (50.2%) | 235 (18.5%) | 54 (23.7%) | 174 (76.3%) | 228 (10.2%) |
|  | Other water sources | No | 269 (25.9%) | 768 (74.1%) | 1037 (95.0%) | 571 (45.6%) | 681 (54.4%) | 1252 (98.6%) | 504 (22.5%) | 1735 (77.5%) | 2239 (100.0%) |
|  |  | Yes | 10 (18.2%) | 45 (81.8%) | 55 (5.0%) | 8 (44.4%) | 10 (55.6%) | 18 (1.4%) | 0 (0%) | 1 (100.0%) | 1 (0.04%) |
| Water distance | | Less 30 min (ref) | 214 (30.7%) | 484 (69.3%) | 698 (63.9%) | 149 (43.4%) | 194 (56.6%) | 343 (27.0%) | 140 (30.8%) | 314 (69.2%) | 454 (20.3%) |
|  |  | 30 min -1 hour | 51 (15.1%) | 287 (84.9%) | 338 (31.0%) | 169 (45.8%) | 200 (54.2%) | 369 (29.1%) | 191 (26.6%) | 528 (73.4%) | 719 (32.1%) |
|  |  | 1-2 hours | 11 (25.0%) | 33 (75.0%) | 44 (4.0%) | 92 (50.0%) | 92 (50.0%) | 184 (14.5%) | 70 (17.0%) | 341 (83.0%) | 411 (18.3%) |
|  |  | Over 2 hours | 3 (25.0%) | 9 (75.0%) | 12 (1.1%) | 169 (45.2%) | 205 (54.8%) | 374 (29.4%) | 103 (15.7%) | 553 (84.3%) | 656 (29.3%) |
| Water shortage | | Yes | 143 (29.4%) | 343 (70.6%) | 486 (44.5%) | 464 (47.4%) | 514 (52.6%) | 978 (77.0%) | 281 (21.0%) | 1054 (79.0%) | 1335 (59.6%) |
|  |  | No | 136 (22.4%) | 470 (77.6%) | 606 (55.5%) | 115 (39.4%) | 177 (60.6%) | 292 (23.0%) | 223 (24.6%) | 682 (75.4%) | 905 (40.4%) |
| Types of toilets | Flush | No | 105 (19.0%) | 447 (81.0%) | 552 (50.5%) | 479 (47.1%) | 539 (52.9%) | 1018 (80.2%) | 437 (22.4%) | 1518 (77.6%) | 1955 (87.3%) |
|  |  | Yes | 174 (32.2%) | 366 (67.8%) | 540 (49.5%) | 100 (39.7%) | 152 (60.3%) | 252 (19.8%) | 67 (23.5%) | 218 (76.5%) | 285 (12.7%) |
|  | Latrine | No | 249 (27.4%) | 661 (72.6%) | 910 (83.3%) | 97 (41.1%) | 139 (58.9%) | 236 (18.6%) | 78 (23.1%) | 260 (76.9%) | 338 (15.1%) |
|  |  | Yes | 30 (16.5%) | 152 (83.5%) | 182 (16.7%) | 482 (46.6%) | 552 (53.4%) | 1034 (81.4%) | 426 (22.4%) | 1476 (77.6%) | 1902 (84.9%) |
|  | Bucket | No | 233 (28.9%) | 572 (71.1%) | 805 (73.7%) | 491 (44.7%) | 607 (55.3%) | 1098 (86.5%) | 335 (26.8%) | 913 (73.2%) | 1248 (55.7%) |
|  |  | Yes | 46 (16.0%) | 241 (84.0%) | 287 (26.3%) | 88 (51.2%) | 84 (48.8%) | 172 (13.5%) | 169 (17.0%) | 823 (83.0%) | 992 (44.3%) |
|  | Open defecation | No | 223 (24.6%) | 683 (75.4%) | 906 (83.0%) | 574 (45.4%) | 689 (54.6%) | 1263 (99.4%) | 501 (22.5%) | 1729 (77.5%) | 2230 (99.6%) |
|  |  | Yes | 56 (30.1%) | 130 (69.9%) | 186 (17.0%) | 5 (71.4%) | 2 (28.6%) | 7 (0.6%) | 3 (30.0%) | 7 (70.0%) | 10 (0.4%) |
| Shared toilet | | Yes | 188 (28.4%) | 475 (71.6%) | 663 (60.7%) | 453 (47.9%) | 493 (52.1%) | 946 (74.5%) | 403 (23.8%) | 1291 (76.2%) | 1694 (75.6%) |
|  |  | No | 91 (21.2%) | 338 (78.8%) | 429 (39.3%) | 126 (38.9%) | 198 (61.1%) | 324 (25.5%) | 101 (18.5%) | 445 (81.5%) | 546 (24.4%) |
|  | Around house | No | 277 (25.5%) | 809 (74.5%) | 1086 (99.5%) | 242 (43.1%) | 319 (56.9%) | 561 (44.2%) | 139 (22.8%) | 471 (77.2%) | 610 (27.2%) |
|  |  | Yes | 2 (33.3%) | 4 (66.7%) | 6 (0.5%) | 337 (47.5%) | 372 (52.5%) | 709 (55.8%) | 365 (22.4%) | 1265 (77.6%) | 1630 (72.8%) |
|  | Dumping site | No | 277 (25.6%) | 804 (74.4%) | 1081 (99.0%) | 528 (45.4%) | 636 (54.6%) | 1164 (91.7%) | 455 (22.4%) | 1576 (77.6%) | 2031 (90.7%) |
|  |  | Yes | 2 (18.2%) | 9 (81.8%) | 11 (1.0%) | 51 (48.1%) | 55 (51.9%) | 106 (8.3%) | 49 (23.4%) | 160 (76.6%) | 209 (9.3%) |
|  | Drainage | No | 227 (24.0%) | 720 (76.0%) | 947 (86.7%) | 431 (43.7%) | 556 (56.3%) | 987 (77.7%) | 395 (24.1%) | 1246 (75.9%) | 1641 (73.3%) |
|  |  | Yes | 52 (35.9%) | 93 (64.1%) | 145 (13.3%) | 148 (52.3%) | 135 (47.7%) | 283 (22.3%) | 109 (18.2%) | 490 (81.8%) | 599 (26.7%) |
|  | Solid waste collectors | No | 278 (25.6%) | 806 (74.4%) | 1084 (99.3%) | 456 (47.8%) | 497 (52.2%) | 953 (75.0%) | 412 (23.8%) | 1321 (76.2%) | 1733 (77.4%) |
|  |  | Yes | 1 (12.5%) | 7 (87.5%) | 8 (0.7%) | 123 (38.8%) | 194 (61.2%) | 317 (25.0%) | 92 (18.1%) | 415 (81.9%) | 507 (22.6%) |
|  | Waste others | No | 279 (25.7%) | 805 (74.3%) | 1084 (99.3%) | 503 (44.4%) | 629 (55.6%) | 1132 (89.1%) | 495 (22.3%) | 1721 (77.7%) | 2216 (98.9%) |
|  |  | Yes | 0 (0%) | 8 (100.0%) | 8 (0.7%) | 76 (55.1%) | 62 (44.9%) | 138 (10.9%) | 9 (37.5%) | 15 (62.5%) | 24 (1.1%) |
| Waste payment | | No | 271 (25.3%) | 801 (74.7%) | 1072 (98.2%) | 434 (47.5%) | 480 (52.5%) | 914 (72.0%) | 400 (23.8%) | 1278 (76.2%) | 1678 (74.9%) |
|  |  | Yes | 8 (40.0%) | 12 (60.0%) | 20 (1.8%) | 145 (40.7%) | 211 (59.3%) | 356 (28.0%) | 104 (18.5%) | 458 (81.5%) | 562 (25.1%) |
| Environmental disaster | | Yes | 461 (42.8%) | 617 (57.2%) | 1078 (98.7%) | 803 (73.6%) | 288 (26.4%) | 1091 (85.9%) | 1613 (77.1%) | 480 (22.9%) | 2093 (93.4%) |
|  |  | No | 3 (21.4%) | 11 (78.6%) | 14 (1.3%) | 134 (74.9%) | 45 (25.1%) | 179 (14.1%) | 91 (61.9%) | 56 (38.1%) | 147 (6.6%) |
| **Enabling factors** | | | | | | | | |  |  |  |
| Income generating activity | Yes | 194 (27.25%) | 518 (72.75%) | 712 (65.20%) | 340 (46.90%) | 385 (53.10%) | 725 (57.09%) | 286 (23.77%) | 917 (76.23%) | 1203 (53.71%) | 194 (27.25%) |
|  | No | 63 (22.03%) | 223 (77.97%) | 286 (26.19%) | 159 (41.84%) | 221 (58.16%) | 380 (29.92%) | 155 (21.77%) | 557 (78.23%) | 712 (31.79%) | 63 (22.03%) |
| Household tenure | Tenant | 13 (24.53%) | 40 (75.47%) | 53 (4.85%) | 62 (47.69%) | 68 (52.31%) | 130 (10.24%) | 54 (19.78%) | 219 (80.22%) | 273 (12.19%) | 13 (24.53%) |
|  | landlord | 9 (21.95%) | 32 (78.05%) | 41 (3.75%) | 18 (51.43%) | 17 (48.57%) | 35 (2.76%) | 9 (17.31%) | 43 (82.69%) | 52 (2.32%) | 9 (21.95%) |
|  | Free living | 194 (27.25%) | 518 (72.75%) | 712 (65.20%) | 340 (46.90%) | 385 (53.10%) | 725 (57.09%) | 286 (23.77%) | 917 (76.23%) | 1203 (53.71%) | 194 (27.25%) |
|  | Caretaker, lease, temporary stay, others | 63 (22.03%) | 223 (77.97%) | 286 (26.19%) | 159 (41.84%) | 221 (58.16%) | 380 (29.92%) | 155 (21.77%) | 557 (78.23%) | 712 (31.79%) | 63 (22.03%) |
| Sources of income | fishing | No | 275 (25.6%) | 800 (74.4%) | 1075 (98.4%) | 578 (45.7%) | 688 (54.3%) | 1266 (99.7%) | 500 (22.5%) | 1722 (77.5%) | 2222 (99.2%) |
|  |  | Yes | 4 (23.5%) | 13 (76.5%) | 17 (1.6%) | 1 (25.0%) | 3 (75.0%) | 4 (0.3%) | 4 (22.2%) | 14 (77.8%) | 18 (0.8%) |
|  | Private salaried | No | 233 (25.1%) | 696 (74.9%) | 929 (85.1%) | 476 (45.9%) | 562 (54.1%) | 1038 (81.7%) | 447 (21.7%) | 1615 (78.3%) | 2062 (92.1%) |
|  |  | Yes | 46 (28.2%) | 117 (71.8%) | 163 (14.9%) | 103 (44.4%) | 129 (55.6%) | 232 (18.3%) | 57 (32.0%) | 121 (68.0%) | 178 (7.9%) |
|  | Informal salaried | No | 279 (25.6%) | 811 (74.4%) | 1090 (99.8%) | 577 (45.8%) | 684 (54.2%) | 1261 (99.3%) | 500 (22.7%) | 1699 (77.3%) | 2199 (98.2%) |
|  |  | Yes | 0 (0%) | 2 (100.0%) | 2 (0.2%) | 2 (22.2%) | 7 (77.8%) | 9 (0.7%) | 4 (9.8%) | 37 (90.2%) | 41 (1.8%) |
|  | Daily wage | No | 259 (24.5%) | 797 (75.5%) | 1056 (96.7%) | 533 (45.9%) | 628 (54.1%) | 1161 (91.4%) | 469 (23.2%) | 1555 (76.8%) | 2024 (90.4%) |
|  |  | Yes | 20 (55.6%) | 16 (44.4%) | 36 (3.3%) | 46 (42.2%) | 63 (57.8%) | 109 (8.6%) | 35 (16.2%) | 181 (83.8%) | 216 (9.6%) |
|  | Bike ride | No | 267 (25.3%) | 789 (74.7%) | 1056 (96.7%) | 560 (45.6%) | 667 (54.4%) | 1227 (96.6%) | 441 (22.1%) | 1552 (77.9%) | 1993 (89.0%) |
|  |  | Yes | 12 (33.3%) | 24 (66.7%) | 36 (3.3%) | 19 (44.2%) | 24 (55.8%) | 43 (3.4%) | 63 (25.5%) | 184 (74.5%) | 247 (11.0%) |
|  | Others | No | 251 (25.4%) | 738 (74.6%) | 989 (90.6%) | 522 (44.7%) | 647 (55.3%) | 1169 (92.0%) | 486 (22.8%) | 1648 (77.2%) | 2134 (95.3%) |
|  |  | Yes | 28 (27.2%) | 75 (72.8%) | 103 (9.4%) | 57 (56.4%) | 44 (43.6%) | 101 (8.0%) | 18 (17.0%) | 88 (83.0%) | 106 (4.7%) |
| Total | |  | 279 (25.5%) | 813 (74.5%) | 1092 (100.0%) | 579 (45.6%) | 691 (54.4%) | 1270 (100.0%) | 504 (22.5%) | 1736 (77.5%) | 2240 (100.0%) |

Table S3: Frequencies and percentages of health utilisation outside informal settlement

| Variable name | | Category | **Cockle Bay** | | | **Dwazark** | | | **Moyiba** | | |
| --- | --- | --- | --- | --- | --- | --- | --- | --- | --- | --- | --- |
|  |  |  | Frequency (%) | | | Frequency (%) | | | Frequency (%) | | |
|  |  |  | Yes | No | Total | No | Yes | Total | No | Yes | Total |
| **Predisposing factors** | | | | | | | | | | | |
| Head of household gender | | Male | 334 (42.1%) | 460 (57.9%) | 794 (72.5%) | 655 (72.5%) | 248 (27.5%) | 903 (71.0%) | 1242 (75.1%) | 411 (24.9%) | 1653 (73.5%) |
|  |  | Female | 133 (44.2%) | 168 (55.8%) | 301 (27.5%) | 282 (76.6%) | 86 (23.4%) | 368 (29.0%) | 470 (78.7%) | 127 (21.3%) | 597 (26.5%) |
| Disability in household | | Yes | 14 (25.9%) | 40 (74.1%) | 54 (4.9%) | 59 (57.8%) | 43 (42.2%) | 102 (8.0%) | 95 (66.0%) | 49 (34.0%) | 144 (6.4%) |
|  |  | No | 453 (43.5%) | 588 (56.5%) | 1041 (95.1%) | 878 (75.1%) | 291 (24.9%) | 1169 (92.0%) | 1617 (76.8%) | 489 (23.2%) | 2106 (93.6%) |
| Family type | | Single | 195 (54.3%) | 164 (45.7%) | 359 (32.8%) | 221 (75.2%) | 73 (24.8%) | 294 (23.1%) | 394 (76.5%) | 121 (23.5%) | 515 (22.9%) |
|  |  | Married, cohabit, engaged | 243 (37.9%) | 399 (62.1%) | 642 (58.6%) | 554 (71.4%) | 222 (28.6%) | 776 (61.1%) | 1151 (76.7%) | 350 (23.3%) | 1501 (66.7%) |
|  |  | Divorced, separated, widowed | 29 (30.9%) | 65 (69.1%) | 94 (8.6%) | 162 (80.6%) | 39 (19.4%) | 201 (15.8%) | 167 (71.4%) | 67 (28.6%) | 234 (10.4%) |
| Income generating activity | | Yes | 306 (38.2%) | 495 (61.8%) | 801 (73.2%) | 691 (72.7%) | 260 (27.3%) | 951 (74.8%) | 1320 (76.3%) | 410 (23.7%) | 1730 (76.9%) |
|  |  | No | 161 (54.8%) | 133 (45.2%) | 294 (26.8%) | 246 (76.9%) | 74 (23.1%) | 320 (25.2%) | 392 (75.4%) | 128 (24.6%) | 520 (23.1%) |
| Food security | | Food secure | 267 (45.8%) | 316 (54.2%) | 583 (53.2%) | 361 (77.5%) | 105 (22.5%) | 466 (36.7%) | 1134 (87.3%) | 165 (12.7%) | 1299 (57.7%) |
|  |  | Food insecure | 200 (39.1%) | 312 (60.9%) | 512 (46.8%) | 576 (71.6%) | 229 (28.4%) | 805 (63.3%) | 578 (60.8%) | 373 (39.2%) | 951 (42.3%) |
| Length of residence | | 0-1 years | 53 (53.0%) | 47 (47.0%) | 100 (9.1%) | 65 (71.4%) | 26 (28.6%) | 91 (7.2%) | 113 (76.9%) | 34 (23.1%) | 147 (6.5%) |
|  |  | 1-5 years | 165 (43.1%) | 218 (56.9%) | 383 (35.0%) | 199 (74.8%) | 67 (25.2%) | 266 (20.9%) | 561 (76.8%) | 169 (23.2%) | 730 (32.4%) |
|  |  | 6-10 years | 99 (43.6%) | 128 (56.4%) | 227 (20.7%) | 144 (73.1%) | 53 (26.9%) | 197 (15.5%) | 384 (77.3%) | 113 (22.7%) | 497 (22.1%) |
|  |  | More than 10 years | 150 (39.0%) | 235 (61.0%) | 385 (35.2%) | 529 (73.8%) | 188 (26.2%) | 717 (56.4%) | 654 (74.7%) | 222 (25.3%) | 876 (38.9%) |
| Household tenure | | Tenant | 310 (43.36%) | 405 (56.64%) | 715 (65.30%) | 544 (74.93%) | 182 (25.07%) | 726 (57.12%) | 929 (76.90%) | 279 (23.10%) | 1208 (53.69%) |
|  |  | landlord | 121 (42.31%) | 165 (57.69%) | 286 (26.12%) | 264 (69.47%) | 116 (30.53%) | 380 (29.90%) | 537 (75.00%) | 179 (25.00%) | 716 (31.82%) |
|  |  | Free living | 18 (33.96%) | 35 (66.04%) | 53 (4.84%) | 102 (78.46%) | 28 (21.54%) | 130 (10.23%) | 207 (75.55%) | 67 (24.45%) | 274 (12.18%) |
|  |  | Caretaker, lease, temporary stay, others | 18 (43.90%) | 23 (56.10%) | 41 (3.74%) | 27 (77.14%) | 8 (22.86%) | 35 (2.75%) | 39 (75.00%) | 13 (25.00%) | 52 (2.31%) |
| Water Source | Piped Dwelling | No | 439 (42.8%) | 586 (57.2%) | 1025 (93.6%) | 907 (73.3%) | 331 (26.7%) | 1238 (97.4%) | 1666 (75.9%) | 529 (24.1%) | 2195 (97.6%) |
|  |  | Yes | 28 (40.0%) | 42 (60.0%) | 70 (6.4%) | 30 (90.9%) | 3 (9.1%) | 33 (2.6%) | 46 (83.6%) | 9 (16.4%) | 55 (2.4%) |
|  | Piped neighbor | No | 380 (44.9%) | 467 (55.1%) | 847 (77.4%) | 925 (73.8%) | 328 (26.2%) | 1253 (98.6%) | 1659 (76.2%) | 518 (23.8%) | 2177 (96.8%) |
|  |  | Yes | 87 (35.1%) | 161 (64.9%) | 248 (22.6%) | 12 (66.7%) | 6 (33.3%) | 18 (1.4%) | 53 (72.6%) | 20 (27.4%) | 73 (3.2%) |
|  | piped compound | No | 443 (43.0%) | 587 (57.0%) | 1030 (94.1%) | 920 (73.6%) | 330 (26.4%) | 1250 (98.3%) | 1681 (76.1%) | 528 (23.9%) | 2209 (98.2%) |
|  |  | Yes | 24 (36.9%) | 41 (63.1%) | 65 (5.9%) | 17 (81.0%) | 4 (19.0%) | 21 (1.7%) | 31 (75.6%) | 10 (24.4%) | 41 (1.8%) |
|  | Public tap/standpipe | No | 374 (43.3%) | 490 (56.7%) | 864 (78.9%) | 830 (74.0%) | 292 (26.0%) | 1122 (88.3%) | 1197 (75.8%) | 383 (24.2%) | 1580 (70.2%) |
|  |  | Yes | 93 (40.3%) | 138 (59.7%) | 231 (21.1%) | 107 (71.8%) | 42 (28.2%) | 149 (11.7%) | 515 (76.9%) | 155 (23.1%) | 670 (29.8%) |
|  | Rainwater | No | 291 (37.6%) | 482 (62.4%) | 773 (70.6%) | 478 (71.6%) | 190 (28.4%) | 668 (52.6%) | 1130 (72.5%) | 428 (27.5%) | 1558 (69.2%) |
|  |  | Yes | 176 (54.7%) | 146 (45.3%) | 322 (29.4%) | 459 (76.1%) | 144 (23.9%) | 603 (47.4%) | 582 (84.1%) | 110 (15.9%) | 692 (30.8%) |
|  | Bowser water | No | 388 (45.4%) | 467 (54.6%) | 855 (78.1%) | 882 (73.2%) | 323 (26.8%) | 1205 (94.8%) | 1702 (76.1%) | 536 (23.9%) | 2238 (99.5%) |
|  |  | Yes | 79 (32.9%) | 161 (67.1%) | 240 (21.9%) | 55 (83.3%) | 11 (16.7%) | 66 (5.2%) | 10 (83.3%) | 2 (16.7%) | 12 (0.5%) |
|  | Kiosk water | No | 459 (42.7%) | 615 (57.3%) | 1074 (98.1%) | 894 (73.2%) | 327 (26.8%) | 1221 (96.1%) | 1589 (77.6%) | 459 (22.4%) | 2048 (91.0%) |
|  |  | Yes | 8 (38.1%) | 13 (61.9%) | 21 (1.9%) | 43 (86.0%) | 7 (14.0%) | 50 (3.9%) | 123 (60.9%) | 79 (39.1%) | 202 (9.0%) |
|  | Bottled water | No | 464 (42.8%) | 620 (57.2%) | 1084 (99.0%) | 923 (73.9%) | 326 (26.1%) | 1249 (98.3%) | 1695 (76.3%) | 526 (23.7%) | 2221 (98.7%) |
|  |  | Yes | 3 (27.3%) | 8 (72.7%) | 11 (1.0%) | 14 (63.6%) | 8 (36.4%) | 22 (1.7%) | 17 (58.6%) | 12 (41.4%) | 29 (1.3%) |
|  | Sachet water | No | 193 (59.9%) | 129 (40.1%) | 322 (29.4%) | 445 (69.3%) | 197 (30.7%) | 642 (50.5%) | 787 (80.2%) | 194 (19.8%) | 981 (43.6%) |
|  |  | Yes | 274 (35.4%) | 499 (64.6%) | 773 (70.6%) | 492 (78.2%) | 137 (21.8%) | 629 (49.5%) | 925 (72.9%) | 344 (27.1%) | 1269 (56.4%) |
|  | Surface water | No | 463 (42.6%) | 624 (57.4%) | 1087 (99.3%) | 837 (73.1%) | 308 (26.9%) | 1145 (90.1%) | 1396 (74.4%) | 481 (25.6%) | 1877 (83.4%) |
|  |  | Yes | 4 (50.0%) | 4 (50.0%) | 8 (0.7%) | 100 (79.4%) | 26 (20.6%) | 126 (9.9%) | 316 (84.7%) | 57 (15.3%) | 373 (16.6%) |
|  | Neighbor’s well | No | 398 (47.4%) | 442 (52.6%) | 840 (76.7%) | 757 (73.1%) | 279 (26.9%) | 1036 (81.5%) | 1548 (76.6%) | 473 (23.4%) | 2021 (89.8%) |
|  |  | Yes | 69 (27.1%) | 186 (72.9%) | 255 (23.3%) | 180 (76.6%) | 55 (23.4%) | 235 (18.5%) | 164 (71.6%) | 65 (28.4%) | 229 (10.2%) |
|  | Other water sources | No | 193 (59.9%) | 129 (40.1%) | 322 (29.4%) | 930 (74.2%) | 323 (25.8%) | 1253 (98.6%) | 1711 (76.1%) | 538 (23.9%) | 2249 (100.0%) |
|  |  | Yes | 274 (35.4%) | 499 (64.6%) | 773 (70.6%) | 7 (38.9%) | 11 (61.1%) | 18 (1.4%) | 1 (100.0%) | 0 (0%) | 1 (0.04%) |
|  | Compound well | NO | 445 (42.7%) | 597 (57.3%) | 1042 (95.2%) | 595 (75.2%) | 196 (24.8%) | 791 (62.2%) | 1592 (76.4%) | 491 (23.6%) | 2083 (92.6%) |
|  |  | Yes | 22 (41.5%) | 31 (58.5%) | 53 (4.8%) | 342 (71.2%) | 138 (28.7%) | 480 (37.8%) | 120 (71.9%) | 47 (28.1%) | 167 (7.4%) |
|  | Spring water | No | 1222 (77.3%) | 359 (22.7%) | 1581 (70.3%) | 883 (73.5%) | 318 (26.5%) | 1201 (94.5%) | 1222 (77.3%) | 359 (22.7%) | 1581 (70.3%) |
|  |  | Yes | 490 (73.2%) | 179 (26.8%) | 669 (29.7%) | 54 (77.1%) | 16 (22.9%) | 70 (5.5%) | 490 (73.2%) | 179 (26.8%) | 669 (29.7%) |
| Water distance | | Less 30 min (ref) | 286 (40.9%) | 414 (59.1%) | 700 (63.9%) | 263 (76.7%) | 80 (23.3%) | 343 (27.0%) | 358 (78.3%) | 99 (21.7%) | 457 (20.3%) |
|  |  | 30 min -1 hour | 169 (49.9%) | 170 (50.1%) | 339 (31.0%) | 272 (73.7%) | 97 (26.3%) | 369 (29.0%) | 587 (81.3%) | 135 (18.7%) | 722 (32.1%) |
|  |  | 1-2 hours | 10 (22.7%) | 34 (77.3%) | 44 (4.0%) | 141 (76.6%) | 43 (23.4%) | 184 (14.5%) | 290 (70.2%) | 123 (29.8%) | 413 (18.4%) |
|  |  | Over 2 hours | 2 (16.7%) | 10 (83.3%) | 12 (1.1%) | 261 (69.6%) | 114 (30.4%) | 375 (29.5%) | 477 (72.5%) | 181 (27.5%) | 658 (29.2%) |
| Water shortage | | Yes | 192 (39.4%) | 295 (60.6%) | 487 (44.5%) | 708 (72.3%) | 271 (27.7%) | 979 (77.0%) | 1015 (75.9%) | 322 (24.1%) | 1337 (59.4%) |
|  |  | No | 275 (45.2%) | 333 (54.8%) | 608 (55.5%) | 229 (78.4%) | 63 (21.6%) | 292 (23.0%) | 697 (76.3%) | 216 (23.7%) | 913 (40.6%) |
| Types of toilets | Flush | No | 259 (46.7%) | 296 (53.3%) | 555 (50.7%) | 759 (74.5%) | 260 (25.5%) | 1019 (80.2%) | 1519 (77.4%) | 444 (22.6%) | 1963 (87.2%) |
|  |  | Yes | 208 (38.5%) | 332 (61.5%) | 540 (49.3%) | 178 (70.6%) | 74 (29.4%) | 252 (19.8%) | 193 (67.2%) | 94 (32.8%) | 287 (12.8%) |
|  | Latrine | No | 382 (41.9%) | 530 (58.1%) | 912 (83.3%) | 160 (67.8%) | 76 (32.2%) | 236 (18.6%) | 253 (74.6%) | 86 (25.4%) | 339 (15.1%) |
|  |  | Yes | 85 (46.4%) | 98 (53.6%) | 183 (16.7%) | 777 (75.1%) | 258 (24.9%) | 1035 (81.4%) | 1459 (76.3%) | 452 (23.7%) | 1911 (84.9%) |
|  | Bucket | No | 338 (41.9%) | 468 (58.1%) | 806 (73.6%) | 801 (72.9%) | 298 (27.1%) | 1099 (86.5%) | 922 (73.7%) | 329 (26.3%) | 1251 (55.6%) |
|  |  | Yes | 129 (44.6%) | 160 (55.4%) | 289 (26.4%) | 136 (79.1%) | 36 (20.9%) | 172 (13.5%) | 790 (79.1%) | 209 (20.9%) | 999 (44.4%) |
|  | Open defecation | No | 398 (43.8%) | 511 (56.2%) | 909 (83.0%) | 932 (73.7%) | 332 (26.3%) | 1264 (99.4%) |  |  |  |
|  |  | Yes | 69 (37.1%) | 117 (62.9%) | 186 (17.0%) | 5 (71.4%) | 2 (28.6%) | 7 (0.6%) |  |  |  |
|  | Flying toilet | No | 463 (42.6%) | 624 (57.4%) | 1087 (99.3%) | 935 (73.9%) | 331 (26.1%) | 1266 (99.6%) | 1706 (76.1%) | 536 (23.9%) | 2242 (99.6%) |
|  |  | Yes | 4 (50.0%) | 4 (50.0%) | 8 (0.7%) | 2 (40.0%) | 3 (60.0%) | 5 (0.4%) | 6 (75.0%) | 2 (25.0%) | 8 (0.4%) |
|  | Open defecation | No | 451 (44.3%) | 566 (55.7%) | 1017 (92.9%) | 932 (73.7%) | 332 (26.3%) | 1264 (99.4%) | 1705 (76.2%) | 534 (23.8%) | 2239 (99.5%) |
|  |  | Yes | 16 (20.5%) | 62 (79.5%) | 78 (7.1%) | 5 (71.4%) | 2 (28.6%) | 7 (0.6%) | 7 (63.6%) | 4 (36.4%) | 11 (0.5%) |
|  | Other toilets | No | 456 (42.3%) | 622 (57.7%) | 1078 (98.4%) | 936 (73.7%) | 334 (26.3%) | 1270 (99.9%) | 1707 (76.1%) | 536 (23.9%) | 2243 (99.7%) |
|  |  | Yes | 11 (64.7%) | 6 (35.3%) | 17 (1.6%) | 1 (100.0%) | 0 (0%) | 1 (0.1%) | 5 (71.4%) | 2 (28.6%) | 7 (0.3%) |
|  | Toilet hanging | No | 398 (43.8%) | 511 (56.2%) | 909 (83.0%) | 928 (73.7%) | 332 (26.3%) | 1260 (99.1%) | 1647 (76.0%) | 519 (24.0%) | 2166 (96.3%) |
|  |  | Yes | 69 (37.1%) | 117 (62.9%) | 186 (17.0%) | 9 (81.8%) | 2 (18.2%) | 11 (0.9%) | 65 (77.4%) | 19 (22.6%) | 84 (3.7%) |
| Shared toilet | | Yes | 278 (41.8%) | 387 (58.2%) | 665 (60.7%) | 711 (75.1%) | 236 (24.9%) | 947 (74.5%) | 1304 (76.5%) | 400 (23.5%) | 1704 (75.7%) |
|  |  | No | 189 (44.0%) | 241 (56.0%) | 430 (39.3%) | 226 (69.8%) | 98 (30.2%) | 324 (25.5%) | 408 (74.7%) | 138 (25.3%) | 546 (24.3%) |
| Toilet access | | Yes | 424 (44.2%) | 535 (55.8%) | 959 (87.6%) | 761 (72.0%) | 296 (28.0%) | 1057 (83.2%) | 1473 (77.2%) | 435 (22.8%) | 1908 (84.8%) |
|  |  | No | 43 (31.6%) | 93 (68.4%) | 136 (12.4%) | 176 (82.2%) | 38 (17.8%) | 214 (16.8%) | 239 (69.9%) | 103 (30.1%) | 342 (15.2%) |
| Waste disposal | Around community | Yes | 6 (26.1%) | 17 (73.9%) | 23 (2.1%) | 48 (82.8%) | 10 (17.2%) | 58 (4.6%) | 463 (75.8%) | 148 (24.2%) | 611 (27.2%) |
|  |  | No | 461 (43.0%) | 611 (57.0%) | 1072 (97.9%) | 889 (73.3%) | 324 (26.7%) | 1213 (95.4%) | 1249 (76.2%) | 390 (23.8%) | 1639 (72.8%) |
|  | Around house | No | 467 (42.9%) | 622 (57.1%) | 1089 (99.5%) | 412 (73.4%) | 149 (26.6%) | 561 (44.1%) | 463 (75.8%) | 148 (24.2%) | 611 (27.2%) |
|  |  | Yes | 0 (0%) | 6 (100.0%) | 6 (0.5%) | 525 (73.9%) | 185 (26.1%) | 710 (55.9%) | 1249 (76.2%) | 390 (23.8%) | 1639 (72.8%) |
|  | Dumping site | No | 463 (42.7%) | 621 (57.3%) | 1084 (99.0%) | 850 (73.0%) | 315 (27.0%) | 1165 (91.7%) | 1570 (77.0%) | 470 (23.0%) | 2040 (90.7%) |
|  |  | Yes | 4 (36.4%) | 7 (63.6%) | 11 (1.0%) | 87 (82.1%) | 19 (17.9%) | 106 (8.3%) | 142 (67.6%) | 68 (32.4%) | 210 (9.3%) |
|  | Drainage | No | 398 (41.9%) | 551 (58.1%) | 949 (86.7%) | 727 (73.6%) | 261 (26.4%) | 988 (77.7%) | 1249 (75.8%) | 399 (24.2%) | 1648 (73.2%) |
|  |  | Yes | 69 (47.3%) | 77 (52.7%) | 146 (13.3%) | 210 (74.2%) | 73 (25.8%) | 283 (22.3%) | 463 (76.9%) | 139 (23.1%) | 602 (26.8%) |
|  | Solid waste collectors | No | 464 (42.7%) | 623 (57.3%) | 1087 (99.3%) | 699 (73.3%) | 254 (26.7%) | 953 (75.0%) | 1326 (76.2%) | 415 (23.8%) | 1741 (77.4%) |
|  |  | Yes | 3 (37.5%) | 5 (62.5%) | 8 (0.7%) | 238 (74.8%) | 80 (25.2%) | 318 (25.0%) | 386 (75.8%) | 123 (24.2%) | 509 (22.6%) |
|  | Waste sea | No | 14 (35.0%) | 26 (65.0%) | 40 (3.7%) | 924 (73.6%) | 332 (26.4%) | 1256 (98.8%) | 1694 (76.1%) | 531 (23.9%) | 2225 (98.9%) |
|  |  | Yes | 453 (42.9%) | 602 (57.1%) | 1055 (96.3%) | 13 (86.7%) | 2 (13.3%) | 15 (1.2%) | 18 (72.0%) | 7 (28.0%) | 25 (1.1%) |
|  | Waste others | No | 465 (42.8%) | 622 (57.2%) | 1087 (99.3%) | 839 (74.1%) | 294 (25.9%) | 1133 (89.1%) | 1703 (76.5%) | 523 (23.5%) | 2226 (98.9%) |
|  |  | Yes | 2 (25.0%) | 6 (75.0%) | 8 (0.7%) | 98 (71.0%) | 40 (29.0%) | 138 (10.9%) | 9 (37.5%) | 15 (62.5%) | 24 (1.1%) |
| Waste payment | | No | 461 (42.9%) | 614 (57.1%) | 1075 (98.2%) | 673 (73.6%) | 241 (26.4%) | 914 (71.9%) | 1298 (76.9%) | 389 (23.1%) | 1687 (75.0%) |
|  |  | Yes | 6 (30.0%) | 14 (70.0%) | 20 (1.8%) | 264 (73.9%) | 93 (26.1%) | 357 (28.1%) | 414 (73.5%) | 149 (26.5%) | 563 (25.0%) |
| Environmental disaster | | Yes | 464 (42.9%) | 617 (57.1%) | 1081 (98.7%) | 803 (73.5%) | 289 (26.5%) | 1092 (85.9%) | 1619 (77.1%) | 482 (22.9%) | 2101 (93.4%) |
|  |  | No | 3 (21.4%) | 11 (78.6%) | 14 (1.3%) | 134 (74.9%) | 45 (25.1%) | 179 (14.1%) | 93 (62.4%) | 56 (37.6%) | 149 (6.6%) |
| **Enabling factors** | | | | | | | | |  |  |  |
| Income generating activity | | Yes | 306 (38.2%) | 495 (61.8%) | 801 (73.2%) | 691 (72.7%) | 260 (27.3%) | 951 (74.8%) | 1320 (76.3%) | 410 (23.7%) | 1730 (76.9%) |
|  |  | No | 161 (54.8%) | 133 (45.2%) | 294 (26.8%) | 246 (76.9%) | 74 (23.1%) | 320 (25.2%) | 392 (75.4%) | 128 (24.6%) | 520 (23.1%) |
| Household tenure | | Tenant | 310 (43.36%) | 405 (56.64%) | 715 (65.30%) | 544 (74.93%) | 182 (25.07%) | 726 (57.12%) | 929 (76.90%) | 279 (23.10%) | 1208 (53.69%) |
|  |  | landlord | 121 (42.31%) | 165 (57.69%) | 286 (26.12%) | 264 (69.47%) | 116 (30.53%) | 380 (29.90%) | 537 (75.00%) | 179 (25.00%) | 716 (31.82%) |
|  |  | Free living | 18 (33.96%) | 35 (66.04%) | 53 (4.84%) | 102 (78.46%) | 28 (21.54%) | 130 (10.23%) | 207 (75.55%) | 67 (24.45%) | 274 (12.18%) |
|  |  | Caretaker, lease, temporary stay, others | 18 (43.90%) | 23 (56.10%) | 41 (3.74%) | 27 (77.14%) | 8 (22.86%) | 35 (2.75%) | 39 (75.00%) | 13 (25.00%) | 52 (2.31%) |
| Source of income | Business | No | 375 (45.0%) | 459 (55.0%) | 834 (76.2%) | 646 (73.8%) | 229 (26.2%) | 875 (68.8%) | 947 (77.3%) | 278 (22.7%) | 1225 (54.4%) |
|  |  | Yes | 92 (35.2%) | 169 (64.8%) | 261 (23.8%) | 291 (73.5%) | 105 (26.5%) | 396 (31.2%) | 765 (74.6%) | 260 (25.4%) | 1025 (45.6%) |
|  | fishing | No | 463 (42.9%) | 615 (57.1%) | 1078 (98.4%) | 934 (73.7%) | 333 (26.3%) | 1267 (99.7%) | 1700 (76.2%) | 532 (23.8%) | 2232 (99.2%) |
|  |  | Yes | 4 (23.5%) | 13 (76.5%) | 17 (1.6%) | 3 (75.0%) | 1 (25.0%) | 4 (0.3%) | 12 (66.7%) | 6 (33.3%) | 18 (0.8%) |
|  | Government salaried | No | 453 (42.9%) | 603 (57.1%) | 1056 (96.4%) | 828 (73.7%) | 295 (26.3%) | 1123 (88.4%) | 1633 (76.4%) | 504 (23.6%) | 2137 (95.0%) |
|  |  | Yes | 14 (35.9%) | 25 (64.1%) | 39 (3.6%) | 109 (73.6%) | 39 (26.4%) | 148 (11.6%) | 79 (69.9%) | 34 (30.1%) | 113 (5.0%) |
|  | Private salaried | No | 399 (42.8%) | 533 (57.2%) | 932 (85.1%) | 777 (74.8%) | 262 (25.2%) | 1039 (81.7%) | 1574 (76.1%) | 493 (23.9%) | 2067 (91.9%) |
|  |  | Yes | 68 (41.7%) | 95 (58.3%) | 163 (14.9%) | 160 (69.0%) | 72 (31.0%) | 232 (18.3%) | 138 (75.4%) | 45 (24.6%) | 183 (8.1%) |
|  | Informal salaried | No | 466 (42.6%) | 627 (57.4%) | 1093 (99.8%) | 929 (73.6%) | 333 (26.4%) | 1262 (99.3%) | 1691 (76.6%) | 518 (23.4%) | 2209 (98.2%) |
|  |  | Yes | 1 (50.0%) | 1 (50.0%) | 2 (0.2%) | 8 (88.9%) | 1 (11.1%) | 9 (0.7%) | 21 (51.2%) | 20 (48.8%) | 41 (1.8%) |
|  | Daily wage | No | 452 (42.7%) | 607 (57.3%) | 1059 (96.7%) | 849 (73.1%) | 313 (26.9%) | 1162 (91.4%) | 1546 (76.0%) | 487 (24.0%) | 2033 (90.4%) |
|  |  | Yes | 15 (41.7%) | 21 (58.3%) | 36 (3.3%) | 88 (80.7%) | 21 (19.3%) | 109 (8.6%) | 166 (76.5%) | 51 (23.5%) | 217 (9.6%) |
|  | Bike ride | No | 455 (43.0%) | 604 (57.0%) | 1059 (96.7%) | 907 (73.9%) | 321 (26.1%) | 1228 (96.6%) | 1519 (75.9%) | 483 (24.1%) | 2002 (89.0%) |
|  |  | Yes | 12 (33.3%) | 24 (66.7%) | 36 (3.3%) | 30 (69.8%) | 13 (30.2%) | 43 (3.4%) | 193 (77.8%) | 55 (22.2%) | 248 (11.0%) |
|  | Stone mining | No | 466 (42.6%) | 627 (57.4%) | 1093 (99.8%) | 937 (73.7%) | 334 (26.3%) | 1271 (100.0%) | 1593 (75.6%) | 515 (24.4%) | 2108 (93.7%) |
|  |  | Yes | 1 (50.0%) | 1 (50.0%) | 2 (0.2%) | 0 (0%) | 0 (0%) | 0 (0%) | 119 (83.8%) | 23 (16.2%) | 142 (6.3%) |
|  | Unemployed | No | 466 (42.6%) | 628 (57.4%) | 1094 (99.9%) | 935 (73.8%) | 332 (26.2%) | 1267 (99.7%) | 1709 (76.1%) | 538 (23.9%) | 2247 (99.9%) |
|  |  | Yes | 1 (100.0%) | 0 (0%) | 1 (0.1%) | 2 (50.0%) | 2 (50.0%) | 4 (0.3%) | 3 (100.0%) | 0 (0%) | 3 (0.1%) |
|  | Others | No | 439 (44.3%) | 553 (55.7%) | 992 (90.6%) | 866 (74.1%) | 303 (25.9%) | 1169 (92.0%) | 1637 (76.4%) | 506 (23.6%) | 2143 (95.2%) |
|  |  | Yes | 28 (27.2%) | 75 (72.8%) | 103 (9.4%) | 71 (69.6%) | 31 (30.4%) | 102 (8.0%) | 75 (70.1%) | 32 (29.9%) | 107 (4.8%) |
| Total | |  | 467 (42.6%) | 628 (57.4%) | 1095 (100.0%) | 937 (73.7%) | 334 (26.3%) | 1271 (100.0%) | 1712 (76.1%) | 538 (23.9%) | 2250 (100.0%) |

Table S4: Estimates of Absolute Risk (AR) and 95% Confidence Intervals for healthcare utilisation (HU) within settlement in Cockle Bay, Dwazark and Moyiba.

| Variable | | Categories | Cockle Bay | Dwazark | Moyiba |
| --- | --- | --- | --- | --- | --- |
|  |  |  | AR(95%CI) | AR(95%CI) | AR(95%CI) |
| **Predisposing factors** | | | | |  |
| Head of household gender gender | | Female | 0.40(0.37,0.42) | 0.34(0.31,0.36) | 0.42(0.41,0.44) |
|  |  | Male | 0.41(0.38,0.43) | 0.34(0.31,0.36) | 0.41(0.40,0.43) |
| Disability in household | | No | 0.37(0.35,0.40) | 0.31(0.28,0.34) | 0.40(0.38,0.42) |
|  |  | Yes | 0.40(0.38,0.42) | 0.34(0.31,0.36) | 0.42(0.41,0.44) |
| Family type | | Single | 0.40(0.38,0.42) | 0.35(0.33,0.38) | 0.42(0.41,0.44) |
|  |  | Married/cohabit/engaged | 0.39(0.37,0.42) | 0.34(0.31,0.36) | 0.42(0.41,0.43) |
|  |  | Divorced/separated/widowed | 0.41(0.39,0.44) | 0.31(0.28,0.34) | 0.42(0.41,0.44) |
| Food security | | Food secure | 0.41(0.39,0.43) | 0.36(0.33,0.38) | 0.42(0.41,0.43) |
|  |  | Food insecure | 0.39(0.37,0.41) | 0.32(0.30,0.35) | 0.42(0.41,0.44) |
| Length of residence | | 0-1 years | 0.40(0.38,0.42) | 0.33(0.31,0.36) | 0.43(0.41,0.44) |
|  |  | 1-5 years | 0.40(0.38,0.42) | 0.33(0.31,0.36) | 0.43(0.42,0.44) |
|  |  | 6-10 years | 0.40(0.38,0.42) | 0.33(0.31,0.36) | 0.43(0.41,0.44) |
|  |  | > 10 years | 0.40(0.37,0.42) | 0.34(0.31,0.36) | 0.41(0.39,0.42) |
| Water sources | Piped dwelling | No | 0.40(0.37,0.42) | 0.33(0.31,0.36) | 0.42(0.41,0.43) |
|  |  | Yes | 0.42(0.40,0.44) | 0.39(0.36,0.42) | 0.43(0.41,0.44) |
|  | Public tap/standpipe | No | 0.39(0.37,0.41) | 0.34(0.31,0.37) | 0.42(0.41,0.43) |
|  |  | Yes | 0.44(0.42,0.46) | 0.32(0.29,0.34) | 0.42(0.41,0.44) |
|  | Rainwater | No | 0.39(0.37,0.41) | 0.32(0.29,0.34) | 0.42(0.41,0.44) |
|  |  | Yes | 0.42(0.40,0.44) | 0.36(0.33,0.38) | 0.42(0.40,0.43) |
|  | Bowser water | No | 0.40(0.38,0.42) | 0.33(0.31,0.36) | 0.42(0.41,0.43) |
|  |  | Yes | 0.47(0.40,0.54) | 0.38(0.35,0.41) | 0.40(0.37,0.43) |
|  | Sachet water | No | 0.42(0.40,0.44) | 0.31(0.28,0.33) | 0.41(0.40,0.43) |
|  |  | Yes | 0.39(0.37,0.41) | 0.37(0.34,0.39) | 0.43(0.41,0.44) |
|  | Water surface | No | 0.40(0.38,0.42) | 0.33(0.31,0.36) | 0.42(0.40,0.43) |
|  |  | Yes | 0.40(0.35,0.44) | 0.38(0.35,0.40) | 0.45(0.43,0.46) |
|  | Neighbours’ well | No | 0.40(0.38,0.42) | 0.34(0.32,0.37) | 0.42(0.41,0.44) |
|  |  | Yes | 0.39(0.36,0.41) | 0.32(0.29,0.34) | 0.41(0.39,0.42) |
|  | Other water sources | No | 0.40(0.38,0.42) | 0.34(0.31,0.36) | 0.42(0.41,0.43) |
|  |  | Yes | 0.43(0.40,0.45) | 0.39(0.35,0.43) | 0.49(0.46,0.52) |
| Water distance | | Less 30 min | 0.38(0.36,0.41) | 0.34(0.32,0.37) | 0.39(0.38,0.41) |
|  |  | 30 min -1 hour | 0.43(0.41,0.45) | 0.34(0.31,0.36) | 0.41(0.40,0.43) |
|  |  | 1-2 hours | 0.40(0.37,0.43) | 0.32(0.29,0.35) | 0.44(0.43,0.46) |
|  |  | Over 2 hours | 0.40(0.37,0.43) | 0.34(0.31,0.37) | 0.44(0.42,0.45) |
| Water shortage | | No | 0.39(0.37,0.42) | 0.33(0.30,0.36) | 0.43(0.41,0.44) |
|  |  | Yes | 0.40(0.38,0.43) | 0.36(0.33,0.39) | 0.41(0.40,0.43) |
| Toilet types | Flush | No | 0.41(0.39,0.44) | 0.33(0.30,0.36) | 0.42(0.41,0.43) |
|  |  | Yes | 0.38(0.36,0.41) | 0.36(0.34,0.39) | 0.42(0.41,0.44) |
|  | Bucket | No | 0.39(0.37,0.41) | 0.34(0.31,0.36) | 0.41(0.39,0.42) |
|  |  | Yes | 0.42(0.40,0.44) | 0.32(0.29,0.35) | 0.44(0.42,0.45) |
|  | Open defecation | No | 0.40(0.38,0.42) | 0.34(0.31,0.36) | 0.42(0.41,0.43) |
|  |  | Yes | 0.36(0.33,0.39) | 0.22(0.14,0.32) | 0.40(0.38,0.42) |
| Waste disposal | Community | No | 0.38(0.35,0.42) | 0.36(0.33,0.39) | 0.43(0.41,0.44) |
|  |  | Yes | 0.40(0.38,0.42) | 0.34(0.31,0.36) | 0.42(0.41,0.43) |
|  | Around house | No | 0.40(0.38,0.42) | 0.35(0.32,0.38) | 0.42(0.40,0.43) |
|  |  | Yes | 0.40(0.36,0.45) | 0.33(0.30,0.35) | 0.42(0.41,0.44) |
|  | Dumping site | No | 0.40(0.38,0.42) | 0.34(0.31,0.36) | 0.42(0.41,0.43) |
|  |  | Yes | 0.42(0.38,0.46) | 0.32(0.29,0.35) | 0.42(0.41,0.44) |
|  | Drainage | No | 0.38(0.35,0.42) | 0.34(0.32,0.37) | 0.42(0.40,0.43) |
|  |  | Yes | 0.40(0.38,0.42) | 0.31(0.28,0.33) | 0.43(0.42,0.45) |
|  | Solid waste collectors | No | 0.40(0.38,0.42) | 0.33(0.30,0.35) | 0.42(0.41,0.43) |
|  |  | Yes | 0.43(0.41,0.46) | 0.36(0.33,0.39) | 0.43(0.41,0.44) |
|  | Waste sea | No | 0.40(0.36,0.42) | 0.34(0.31,0.36) | 0.42(0.41,0.43) |
|  |  | Yes | 0.40(0.38,0.42) | 0.42(0.39,0.44) | 0.41(0.39,0.43) |
|  | Waste others | No | 0.40(0.38,0.42) | 0.34(0.31,0.37) | 0.42(0.41,0.44) |
|  |  | Yes | 0.45(0.42,0.47) | 0.31(0.28,0.34) | 0.34(0.30,0.38) |
| Waste payment | | No | 0.40(0.38,0.42) | 0.33(0.30,0.35) | 0.42(0.41,0.43) |
|  |  | Yes | 0.36(0.33,0.40) | 0.36(0.33,0.39) | 0.43(0.41,0.44) |
| Environmental disaster | | No | 0.40(0.38,0.42) | 0.34(0.32,0.37) | 0.42(0.41,0.44) |
|  |  | Yes | 0.38(0.33,0.42) | 0.30(0.27,0.33) | 0.36(0.34,0.38) |
| **Enabling factors** | | | | |  |
| Income activity engagement | | No | 0.42(0.40,0.44) | 0.33(0.31,0.36) | 0.42(0.40,0.43) |
|  |  | Yes | 0.39(0.37,0.41) | 0.35(0.32,0.37) | 0.43(0.41,0.44) |
| Household tenure | | Tenant | 0.39(0.37,0.42) | 0.33(0.31,0.36) | 0.42(0.40,0.43) |
|  |  | landlord | 0.41(0.39,0.43) | 0.35(0.33,0.38) | 0.43(0.41,0.44) |
|  |  | Free living | 0.39(0.37,0.42) | 0.33(0.31,0.36) | 0.42(0.41,0.44) |
|  |  | Caretaker/lease/temporary stay/others | 0.40(0.38,0.43) | 0.29(0.25,0.33) | 0.42(0.40,0.44) |
| Sources of income | Business | No | 0.40(0.38,0.42) | 0.33(0.31,0.36) | 0.42(0.40,0.43) |
|  |  | Yes | 0.40(0.38,0.42) | 0.35(0.32,0.37) | 0.42(0.41,0.44) |
|  | fishing | No | 0.40(0.38,0.42) | 0.34(0.31,0.36) | 0.42(0.41,0.43) |
|  |  | Yes | 0.43(0.40,0.46) | 0.42(0.40,0.44) | 0.42(0.40,0.44) |
|  | Private salaried | No | 0.40(0.38,0.42) | 0.33(0.31,0.36) | 0.42(0.41,0.44) |
|  |  | Yes | 0.40(0.38,0.42) | 0.35(0.32,0.37) | 0.39(0.37,0.41) |
|  | Daily wage | No | 0.40(0.38,0.42) | 0.34(0.31,0.36) | 0.42(0.40,0.43) |
|  |  | Yes | 0.26(0.22,0.31) | 0.33(0.30,0.36) | 0.44(0.42,0.45) |
|  | Bike ride | No | 0.40(0.38,0.42) | 0.34(0.31,0.36) | 0.42(0.41,0.44) |
|  |  | Yes | 0.38(0.35,0.42) | 0.32(0.29,0.35) | 0.41(0.40,0.43) |
|  | Stone mining | No | 0.40(0.38,0.42) | 0.34(0.31,0.36) | 0.42(0.41,0.43) |
|  |  | Yes | 0.38(0.35,0.41) | 0.34(0.32,0.37) | 0.42(0.41,0.44) |
|  | Others | No | 0.40(0.38,0.42) | 0.29(0.26,0.32) | 0.42(0.41,0.43) |
|  |  | Yes | 0.39(0.37,0.42) | 0.33(0.31,0.36) | 0.43(0.42,0.45) |

AR (95%CI) = Absolute Risk (95% Confidence Interval); min = Minutes

Table S5: Estimates of Absolute Risk (AR) and 95% Confidence Intervals for healthcare utilisation (HU) outside settlement in Cockle Bay, Dwazark and Moyiba.

|  | | Categories (reference) | Cockle Bay | Dwazark | Moyiba |
| --- | --- | --- | --- | --- | --- |
|  |  |  | AR(95%CI) | AR(95%CI) | AR(95%CI) |
| **Predisposing factors** | | | | |  |
| Head of household gender gender | | Female | 0.36(0.33,0.39) | 0.21(0.18,0.25) | 0.18(0.16,0.21) |
|  |  | Male | 0.36(0.33,0.38) | 0.18(0.15,0.21) | 0.17(0.15,0.19) |
| Disability in household | | No | 0.41(0.38,0.43) | 0.27(0.22,0.31) | 0.23(0.20,0.26) |
|  |  | Yes | 0.36(0.33,0.38) | 0.20(0.16,0.23) | 0.18(0.15,0.20) |
| Family type | | Single | 0.33(0.30,0.35) | 0.21(0.18,0.25) | 0.19(0.17,0.22) |
|  |  | Married/cohabit/engaged | 0.37(0.35,0.40) | 0.21(0.18,0.25) | 0.17(0.15,0.19) |
|  |  | Divorced/separated/widowed | 0.39(0.37,0.42) | 0.15(0.12,0.18) | 0.21(0.19,0.24) |
| Income activity engagement | | No | 0.37(0.35,0.40) | 0.21(0.17,0.24) | 0.18(0.15,0.20) |
|  |  | Yes | 0.32(0.29,0.34) | 0.19(0.16,0.23) | 0.19(0.17,0.22) |
| Food security | | Food secure | 0.36(0.33,0.38) | 0.19(0.16,0.23) | 0.11(0.10,0.13) |
|  |  | Food insecure | 0.36(0.34,0.39) | 0.21(0.17,0.25) | 0.27(0.24,0.30) |
| Length of residence | | 0-1 years | 0.31(0.28,0.35) | 0.23(0.19,0.28) | 0.17(0.14,0.19) |
|  |  | 1-5 years | 0.36(0.33,0.38) | 0.18(0.15,0.22) | 0.17(0.15,0.19) |
|  |  | 6-10 years | 0.36(0.33,0.38) | 0.20(0.17,0.24) | 0.19(0.16,0.21) |
|  |  | > 10 years | 0.37(0.35,0.40) | 0.21(0.17,0.24) | 0.18(0.16,0.21) |
| Water sources | Piped dwelling | No | 0.36(0.33,0.38) | 0.20(0.17,0.24) | 0.18(0.16,0.20) |
|  |  | Yes | 0.37(0.34,0.39) | 0.19(0.15,0.23) | 0.14(0.12,0.16) |
|  | Public tap/standpipe | No (ref) | 0.36(0.33,0.38) | 0.20(0.17,0.24) | 0.18(0.16,0.21) |
|  |  | Yes | 0.36(0.34,0.39) | 0.23(0.19,0.27) | 0.17(0.15,0.20) |
|  | Rainwater | No | 0.37(0.35,0.40) | 0.22(0.18,0.26) | 0.20(0.18,0.23) |
|  |  | Yes | 0.32(0.30,0.34) | 0.19(0.16,0.22) | 0.12(0.11,0.14) |
|  | Bowser water | No | 0.36(0.33,0.38) | 0.20(0.17,0.24) | 0.18(0.16,0.20) |
|  |  | Yes | 0.39(0.27,0.55) | 0.15(0.11,0.19) | 0.15(0.11,0.20) |
|  | Sachet water | No | 0.30(0.28,0.33) | 0.23(0.19,0.27) | 0.16(0.14,0.18) |
|  |  | Yes | 0.38(0.36,0.41) | 0.18(0.15,0.21) | 0.20(0.17,0.22) |
|  | Water surface | No | 0.36(0.33,0.38) | 0.21(0.17,0.24) | 0.19(0.16,0.21) |
|  |  | Yes | 0.34(0.26,0.42) | 0.17(0.14,0.21) | 0.14(0.12,0.16) |
|  | Neighbours’ well | No | 0.35(0.32,0.37) | 0.20(0.17,0.24) | 0.18(0.15,0.20) |
|  |  | Yes | 0.40(0.38,0.43) | 0.19(0.16,0.23) | 0.20(0.18,0.23) |
|  | Other water sources | No | 0.36(0.34,0.39) | 0.20(0.17,0.24) | 0.18(0.16,0.20) |
|  |  | Yes | 0.28(0.25,0.31) | 0.40(0.35,0.44) | 0.00(0.00,1.00) |
| Water distance | | Less 30 min | 0.37(0.34,0.39) | 0.19(0.16,0.22) | 0.16(0.14,0.19) |
|  |  | 30 min -1 hour | 0.33(0.30,0.36) | 0.20(0.17,0.24) | 0.14(0.12,0.16) |
|  |  | 1-2 hours | 0.41(0.39,0.44) | 0.20(0.17,0.25) | 0.22(0.20,0.25) |
|  |  | Over 2 hours | 0.38(0.35,0.41) | 0.21(0.18,0.25) | 0.20(0.18,0.23) |
| Water shortage | | No | 0.37(0.34,0.39) | 0.21(0.18,0.25) | 0.18(0.16,0.20) |
|  |  | Yes | 0.35(0.33,0.38) | 0.17(0.14,0.20) | 0.18(0.15,0.20) |
| Toilet types | Flush | No | 0.34(0.32,0.37) | 0.20(0.17,0.24) | 0.17(0.15,0.20) |
|  |  | Yes | 0.38(0.35,0.40) | 0.22(0.18,0.26) | 0.22(0.19,0.25) |
|  | Bucket | No | 0.36(0.34,0.39) | 0.20(0.17,0.24) | 0.18(0.16,0.20) |
|  |  | Yes | 0.35(0.32,0.37) | 0.19(0.16,0.23) | 0.18(0.15,0.20) |
|  | Open defecation | No | 0.36(0.33,0.38) | 0.20(0.17,0.24) | 0.18(0.16,0.20) |
|  |  | Yes | 0.40(0.38,0.43) | 0.25(0.21,0.30) | 0.24(0.19,0.31) |
| Waste disposal | Community | No | 0.41(0.38,0.45) | 0.16(0.12,0.20) | 0.23(0.20,0.27) |
|  |  | Yes | 0.36(0.33,0.38) | 0.20(0.17,0.24) | 0.18(0.16,0.20) |
|  | Around house | No | 0.36(0.33,0.38) | 0.20(0.16,0.24) | 0.19(0.16,0.21) |
|  |  | Yes | 0.40(0.37,0.43) | 0.21(0.17,0.25) | 0.18(0.15,0.20) |
|  | Dumping site | No | 0.36(0.33,0.38) | 0.21(0.17,0.24) | 0.17(0.15,0.20) |
|  |  | Yes | 0.40(0.36,0.44) | 0.16(0.13,0.20) | 0.22(0.20,0.26) |
|  | Drainage | No | 0.36(0.33,0.38) | 0.20(0.17,0.24) | 0.18(0.16,0.21) |
|  |  | Yes | 0.35(0.33,0.38) | 0.20(0.17,0.24) | 0.17(0.15,0.20) |
|  | Solid waste collectors | No | 0.36(0.33,0.38) | 0.20(0.17,0.24) | 0.18(0.15,0.20) |
|  |  | Yes | 0.41(0.39,0.43) | 0.19(0.16,0.23) | 0.19(0.17,0.21) |
|  | Waste sea | No | 0.41(0.37,0.45) | 0.20(0.17,0.24) | 0.18(0.16,0.20) |
|  |  | Yes | 0.36(0.33,0.38) | 0.13(0.08,0.20) | 0.21(0.16,0.26) |
|  | Waste others | No | 0.36(0.33,0.38) | 0.20(0.17,0.24) | 0.18(0.16,0.20) |
|  |  | Yes | 0.45(0.41,0.48) | 0.22(0.19,0.26) | 0.33(0.29,0.38) |
| Waste payment | | No | 0.36(0.33,0.38) | 0.21(0.17,0.24) | 0.17(0.15,0.20) |
|  |  | Yes | 0.39(0.37,0.42) | 0.19(0.16,0.23) | 0.19(0.17,0.22) |
| Environmental disaster | | No | 0.36(0.33,0.38) | 0.20(0.17,0.24) | 0.17(0.15,0.20) |
|  |  | Yes | 0.42(0.39,0.45) | 0.20(0.16,0.24) | 0.24(0.21,0.27) |
| **Enabling factors** | | | | |  |
| Income activity engagement | | No | 0.37(0.35,0.40) | 0.21(0.17,0.24) | 0.18(0.15,0.20) |
|  |  | Yes | 0.32(0.29,0.34) | 0.19(0.16,0.23) | 0.19(0.17,0.22) |
| Household tenure | | Tenant | 0.35(0.33,0.38) | 0.19(0.16,0.23) | 0.17(0.15,0.19) |
|  |  | landlord | 0.37(0.35,0.39) | 0.22(0.19,0.26) | 0.18(0.16,0.21) |
|  |  | Free living | 0.39(0.36,0.42) | 0.19(0.15,0.23) | 0.19(0.16,0.21) |
|  |  | Caretaker/lease/temporary stay/others | 0.38(0.35,0.41) | 0.20(0.16,0.25) | 0.23(0.20,0.26) |
| Sources of income | Business | No | 0.35(0.33,0.38) | 0.20(0.17,0.24) | 0.18(0.15,0.20) |
|  |  | Yes | 0.39(0.36,0.41) | 0.20(0.17,0.24) | 0.18(0.16,0.21) |
|  | fishing | No | 0.36(0.33,0.38) | 0.20(0.17,0.24) | 0.18(0.16,0.20) |
|  |  | Yes | 0.41(0.39,0.44) | 0.23(0.13,0.36) | 0.28(0.24,0.33) |
|  | Private salaried | No | 0.36(0.34,0.39) | 0.20(0.17,0.24) | 0.18(0.16,0.20) |
|  |  | Yes | 0.35(0.32,0.37) | 0.21(0.18,0.25) | 0.17(0.15,0.20) |
|  | Daily wage | No | 0.36(0.33,0.38) | 0.20(0.17,0.24) | 0.18(0.16,0.20) |
|  |  | Yes | 0.37(0.34,0.39) | 0.18(0.15,0.22) | 0.18(0.16,0.20) |
|  | Bike ride | No | 0.36(0.33,0.38) | 0.20(0.17,0.24) | 0.18(0.16,0.20) |
|  |  | Yes | 0.39(0.36,0.42) | 0.24(0.19,0.29) | 0.17(0.15,0.20) |
|  | Stone mining | No | 0.36(0.33,0.38) | 0.20(0.17,0.24) | 0.18(0.16,0.21) |
|  |  | Yes | 0.41(0.34,0.48) | 0.20(0.17,0.24) | 0.14(0.12,0.16) |
|  | Others | No | 0.35(0.33,0.38) | 0.25(0.21,0.29) | 0.18(0.16,0.20) |
|  |  | Yes | 0.40(0.37,0.42) | 0.20(0.17,0.24) | 0.21(0.18,0.24) |

AR (95%CI) = Absolute Risk (95% Confidence Interval); min = Minutes Table S6: Estimates of Relative Risk (RR) and 95% Confidence Intervals for healthcare utilisation within and outside informal settlement in Cockle Bay, Moyiba, and Dwazark

| Variable | | Categories (reference) | Cockle Bay | Dwazark | Moyiba |
| --- | --- | --- | --- | --- | --- |
|  |  |  | RR(95%CI) | RR(95%CI) | RR(95%CI) |
| **Predisposing factors** | | | | |  |
| Intercept | | | 0.60(0.53,0.69) | 0.64(0.55,0.74) | 0.60(0.52,0.68) |
| Head of household gender | | Female (ref) | 1.00 | 1.00 | 1.00 |
|  |  | Male | 1.10(1.07,1.13) | 0.99(0.94,1.04) | 1.06(1.01,1.1) |
| Disability in household | | No (ref) | 1.00 | 1.00 | 1.00 |
|  |  | Yes | 0.91(0.88,0.94) | 0.92(0.87,0.96) | 0.79(0.76,0.82) |
| Family type | | Single (ref) | 1.00 | 1.00 | 1.00 |
|  |  | Married/cohabit/engaged | 1.07(1.03,1.10) | 0.94(0.89,0.98) | 1.05(1.01,1.1) |
|  |  | Divorced/separated/widowed | 1.02(0.97,1.07) | 0.95(0.9,1.01) | 1.19(1.12,1.27) |
| Income activity engagement | | No (ref) | 1.00 | 1.00 | 1.00 |
|  |  | Yes | 0.89(0.85,0.93) | 0.95(0.91,1) | 0.93(0.89,0.97) |
| Food security | | Food secure (ref) | 1.00 | 1.00 | 1.00 |
|  |  | Food insecure | 0.99(0.97,1.02) | 1.08(1.04,1.13) | 1.42(1.36,1.48) |
| Length of residence | | 0-1 years (ref) | 1.00 | 1.00 | 1.00 |
|  |  | 1-5 years | 1.09(1.06,1.13) | 0.94(0.87,1.01) | 0.88(0.8,0.98) |
|  |  | 6-10 years | 1.16(1.11,1.22) | 0.92(0.85,1) | 0.96(0.87,1.06) |
|  |  | > 10 years | 1.23(1.19,1.28) | 0.96(0.9,1.03) | 1.02(0.93,1.12) |
| Water sources | Piped dwelling | No (ref) | 1.00 | 1.00 | 1.00 |
|  |  | Yes | 0.99(0.93,1.06) | 0.69(0.58,0.83) | 0.99(0.86,1.14) |
|  | Public tap/standpipe | No (ref) | 1.00 | 1.00 | 1.00 |
|  |  | Yes | 0.96(0.94,0.99) | 1.04(0.99,1.1) | 1.02(0.97,1.07) |
|  | Rainwater | No (ref) | 1.00 | 1.00 | 1.00 |
|  |  | Yes | 0.82(0.78,0.87) | 0.91(0.88,0.95) | 1(0.96,1.05) |
|  | Bowser water | No (ref) | 1.00 | 1.00 | 1.00 |
|  |  | Yes | 0.85(0.59,1.22) | 0.8(0.71,0.89) | 1.12(0.94,1.35) |
|  | Sachet water | No (ref) | 1.00 | 1.00 | 1.00 |
|  |  | Yes | 1.19(1.14,1.25) | 0.86(0.83,0.89) | 0.99(0.95,1.03) |
|  | Water surface | No (ref) | 1.00 | 1.00 | 1.00 |
|  |  | Yes | 0.84(0.62,1.14) | 0.8(0.74,0.86) | 0.70(0.64,0.75) |
|  | Neighbours’ well | No (ref) | 1.00 | 1.00 | 1.00 |
|  |  | Yes | 1.15(1.13,1.18) | 0.98(0.93,1.02) | 1.13(1.07,1.19) |
|  | Other water sources | No (ref) | 1.00 | 1.00 | * |
|  |  | Yes | 0.93(0.85,1.02) | 1.04(0.95,1.13) | * |
| Water distance | | Less 30 min (ref) | 1.00 | 1.00 | 1.00 |
|  |  | 30 min -1 hour | 0.86(0.82,0.89) | 1.09(1.04,1.15) | 0.91(0.87,0.96) |
|  |  | 1-2 hours | 0.98(0.93,1.02) | 1.1(1.04,1.17) | 0.88(0.83,0.93) |
|  |  | Over 2 hours | 1.05(1.00,1.11) | 1.07(1.01,1.12) | 0.85(0.81,0.9) |
| Water shortage | | No (ref) | 1.00 | 1.00 | 1.00 |
|  |  | Yes | 0.90(0.88,0.92) | 0.89(0.85,0.94) | 0.90(0.87,0.94) |
| Toilet types | Flush | No (ref) | 1.00 | 1.00 | 1.00 |
|  |  | Yes | 1.12(1.08,1.15) | 1.01(0.96,1.06) | 0.99(0.95,1.04) |
|  | Bucket | No (ref) | 1.00 | 1.00 | 1.00 |
|  |  | Yes | 0.97(0.93,1.00) | 1.07(1.01,1.13) | 0.76(0.73,0.79) |
|  | Open defecation | No (ref) | 1.00 | 1.00 | 1.00 |
|  |  | Yes | 1.15(1.11,1.19) | 1.08(0.88,1.33) | 1.28(1.1,1.48) |
| Waste disposal | Community | No (ref) | 1.00 | 1.00 | 1.00 |
|  |  | Yes | 0.94(0.87,1.01) | 1.17(1.05,1.3) | 0.81(0.74,0.88) |
|  | Around house | No (ref) | 1.00 | 1.00 | 1.00 |
|  |  | Yes | 1.14(1.05,1.24) | 0.98(0.95,1.02) | 1.13(1.07,1.2) |
|  | Dumping site | No (ref) | 1.00 | 1.00 | 1.00 |
|  |  | Yes | 1.11(1.01,1.21) | 0.96(0.89,1.04) | 1.14(1.07,1.21) |
|  | Drainage | No (ref) | 1.00 | 1.00 | 1.00 |
|  |  | Yes | 0.90(0.80,1.01) | 0.91(0.84,0.98) | 0.81(0.75,0.89) |
|  | Solid waste collectors | No (ref) | 1.00 | 1.00 | 1.00 |
|  |  | Yes | 0.95(0.88,1.03) | 0.63(0.47,0.84) | 1.35(1.23,1.49) |
|  | Waste others | No (ref) | 1.00 | 1.00 | 1.00 |
|  |  | Yes | 1.08(0.97,1.21) | 1.26(1.21,1.32) | 1.26(1.21,1.32) |
| Waste payment | | No (ref) | 1.00 | 1.00 | 1.00 |
|  |  | Yes | 1.10(1.08,1.13) | 1.08(1.01,1.15) | 1.19(1.10,1.28) |
| Environmental disaster | | No (ref) | 1.00 | 1.00 | 1.00 |
|  |  | Yes | 1.27(1.17,1.37) | 1.03(0.98,1.08) | 1.23(1.16,1.29) |
| **Enabling factors** | | | | |  |
| Income activity engagement | | No (ref) | 1.00 | 1.00 | 1.00 |
|  |  | Yes | 0.89(0.85,0.93) | 0.95(0.91,1) | 0.93(0.89,0.97) |
| Household tenure | | Tenant | 1.00 | 1.00 | 1.00 |
|  |  | landlord | 1.09(1.07,1.12) | 1.05(1.01,1.09) | 0.97(0.94,1.02) |
|  |  | Free living | 1.00(0.98,1.02) | 0.92(0.86,0.99) | 1(0.95,1.06) |
|  |  | Caretaker/lease/temporary stay/others | 1.17(1.11,1.23) | 0.92(0.82,1.04) | 1.1(0.97,1.25) |
| Sources of income | Business | No(ref) | 1.00 | 1.00 | 1.00 |
|  |  | Yes | 1.08(1.05,1.11) | 1.03(0.99,1.07) | 1.10(1.06,1.14) |
|  | fishing | No(ref) | 1.00 | 1.00 | 1.00 |
|  |  | Yes | 1.11(1.00,1.24) | 1.08(0.77,1.5) | 1.39(1.2,1.61) |
|  | Private salaried | No (ref) | 1.00 | 1.00 | 1.00 |
|  |  | Yes | 1.05(1.03,1.08) | 1(0.95,1.05) | 1.26(1.19,1.33) |
|  | Daily wage | No (ref) | 1.00 | 1.00 | 1.00 |
|  |  | Yes | 1.18(1.14,1.22) | 0.97(0.9,1.05) | 0.84(0.78,0.91) |
|  | Bike ride | No (ref) | 1.00 | 1.00 | 1.00 |
|  |  | Yes | 1.13(1.09,1.18) | 0.99(0.89,1.11) | 1.14(1.08,1.20) |
|  | Stone mining | No (ref) | 1.00 | * | 1.00 |
|  |  | Yes | 1.33(0.85,2.07) | * | 0.91(0.85,0.99) |
|  | Others | No (ref) | 1.00 | 1.00 | 1.00 |
|  |  | Yes | 1.12(1.09,1.16) | 1.13(1.07,1.19) | 0.99(0.91,1.09) |

RR (95%CI) = Relative Risk (95% Confidence Interval); * excluded due to small sample size in a category; min = Minutes

Table S7: Estimates of Absolute Risk (AR) and 95% Confidence Intervals for healthcare utilisation (HU) within and outside settlement in Cockle Bay, Dwazark and Moyiba.

|  | | Categories (reference) | Cockle Bay | Dwazark | Moyiba |
| --- | --- | --- | --- | --- | --- |
|  |  |  | AR(95%CI) | AR(95%CI) | AR(95%CI) |
| **Predisposing factors** | | | | |  |
| Head of household gender gender | | Female | 0.39(0.38,0.41) | 0.37(0.35,0.40) | 0.28(0.27,0.31) |
|  |  | Male | 0.39(0.37,0.41) | 0.37(0.34,0.40) | 0.30(0.28,0.33) |
| Disability in household | | No | 0.43(0.41,0.45) | 0.40(0.37,0.42) | 0.35(0.33,0.37) |
|  |  | Yes | 0.39(0.37,0.41) | 0.37(0.34,0.39) | 0.29(0.27,0.31) |
| Family type | | Single | 0.37(0.35,0.39) | 0.37(0.34,0.40) | 0.28(0.26,0.30) |
|  |  | Married/cohabit/engaged | 0.40(0.38,0.42) | 0.37(0.34,0.39) | 0.29(0.27,0.31) |
|  |  | Divorced/separated/widowed | 0.41(0.39,0.42) | 0.38(0.35,0.40) | 0.33(0.31,0.35) |
| Income activity engagement | | No | 0.40(0.39,0.42) | 0.37(0.35,0.40) | 0.29(0.27,0.32) |
|  |  | Yes | 0.36(0.34,0.37) | 0.36(0.34,0.39) | 0.28(0.26,0.30) |
| Food security | | Food secure | 0.38(0.36,0.40) | 0.35(0.33,0.38) | 0.26(0.24,0.28) |
|  |  | Food insecure | 0.40(0.39,0.42) | 0.38(0.36,0.41) | 0.34(0.32,0.36) |
| Length of residence | | 0-1 years | 0.36(0.34,0.38) | 0.38(0.35,0.40) | 0.28(0.26,0.31) |
|  |  | 1-5 years | 0.38(0.37,0.40) | 0.37(0.34,0.39) | 0.27(0.25,0.28) |
|  |  | 6-10 years | 0.39(0.38,0.41) | 0.36(0.34,0.39) | 0.29(0.27,0.31) |
|  |  | > 10 years | 0.41(0.39,0.42) | 0.37(0.35,0.40) | 0.31(0.29,0.34) |
| Water sources | Piped dwelling | No | 0.39(0.37,0.41) | 0.37(0.35,0.40) | 0.29(0.27,0.31) |
|  |  | Yes | 0.40(0.38,0.42) | 0.28(0.24,0.32) | 0.25(0.22,0.28) |
|  | Public tap/standpipe | No (ref) | 0.39(0.38,0.41) | 0.37(0.34,0.39) | 0.29(0.27,0.31) |
|  |  | Yes | 0.39(0.37,0.40) | 0.39(0.36,0.41) | 0.30(0.28,0.32) |
|  | Rainwater | No | 0.41(0.40,0.43) | 0.38(0.35,0.40) | 0.30(0.28,0.33) |
|  |  | Yes | 0.34(0.33,0.36) | 0.36(0.34,0.39) | 0.26(0.24,0.28) |
|  | Bowser water | No | 0.39(0.37,0.41) | 0.37(0.35,0.40) | 0.29(0.27,0.31) |
|  |  | Yes | 0.42(0.33,0.51) | 0.32(0.29,0.35) | 0.35(0.30,0.39) |
|  | Sachet water | No | 0.34(0.32,0.36) | 0.39(0.37,0.42) | 0.29(0.27,0.31) |
|  |  | Yes | 0.41(0.40,0.43) | 0.35(0.32,0.37) | 0.29(0.27,0.31) |
|  | Water surface | No | 0.39(0.38,0.41) | 0.37(0.35,0.40) | 0.31(0.29,0.33) |
|  |  | Yes | 0.35(0.29,0.42) | 0.33(0.30,0.36) | 0.21(0.19,0.23) |
|  | Neighbours’ well | No | 0.38(0.36,0.40) | 0.37(0.34,0.40) | 0.29(0.27,0.31) |
|  |  | Yes | 0.43(0.42,0.45) | 0.37(0.35,0.40) | 0.32(0.30,0.35) |
|  | Other water sources | No | 0.39(0.38,0.41) | 0.37(0.34,0.40) | 0.29(0.27,0.31) |
|  |  | Yes | 0.35(0.33,0.38) | 0.41(0.39,0.44) | 0.00(0.00,1.00) |
| Water distance | | Less 30 min | 0.40(0.39,0.42) | 0.36(0.33,0.38) | 0.31(0.29,0.33) |
|  |  | 30 min -1 hour | 0.37(0.35,0.38) | 0.37(0.35,0.40) | 0.28(0.26,0.30) |
|  |  | 1-2 hours | 0.43(0.41,0.45) | 0.38(0.35,0.41) | 0.29(0.27,0.32) |
|  |  | Over 2 hours | 0.41(0.39,0.42) | 0.37(0.35,0.40) | 0.28(0.26,0.30) |
| Water shortage | | No | 0.40(0.38,0.42) | 0.38(0.35,0.40) | 0.30(0.28,0.32) |
|  |  | Yes | 0.38(0.37,0.40) | 0.35(0.33,0.38) | 0.28(0.26,0.30) |
| Toilet types | Flush | No | 0.37(0.35,0.39) | 0.37(0.35,0.40) | 0.29(0.27,0.31) |
|  |  | Yes | 0.41(0.40,0.43) | 0.35(0.33,0.38) | 0.30(0.28,0.33) |
|  | Bucket | No | 0.40(0.38,0.42) | 0.37(0.34,0.39) | 0.32(0.30,0.34) |
|  |  | Yes | 0.37(0.36,0.39) | 0.37(0.35,0.40) | 0.26(0.24,0.28) |
|  | Open defecation | No | 0.39(0.37,0.41) | 0.37(0.34,0.40) | 0.29(0.27,0.31) |
|  |  | Yes | 0.43(0.41,0.44) | 0.42(0.36,0.48) | 0.38(0.34,0.42) |
| Waste disposal | Community | No | 0.46(0.43,0.48) | 0.33(0.29,0.36) | 0.31(0.28,0.34) |
|  |  | Yes | 0.39(0.37,0.41) | 0.37(0.35,0.40) | 0.29(0.27,0.31) |
|  | Around house | No | 0.39(0.37,0.41) | 0.37(0.34,0.39) | 0.29(0.27,0.32) |
|  |  | Yes | 0.46(0.44,0.47) | 0.37(0.35,0.40) | 0.29(0.27,0.31) |
|  | Dumping site | No | 0.39(0.37,0.41) | 0.37(0.35,0.40) | 0.29(0.27,0.31) |
|  |  | Yes | 0.43(0.41,0.45) | 0.35(0.32,0.38) | 0.31(0.29,0.34) |
|  | Drainage | No | 0.39(0.38,0.41) | 0.37(0.34,0.39) | 0.30(0.28,0.32) |
|  |  | Yes | 0.38(0.36,0.40) | 0.37(0.35,0.40) | 0.27(0.25,0.29) |
|  | Solid waste collectors | No | 0.39(0.37,0.41) | 0.38(0.35,0.40) | 0.29(0.27,0.31) |
|  |  | Yes | 0.44(0.41,0.46) | 0.35(0.33,0.38) | 0.29(0.27,0.31) |
|  | Waste sea | No | 0.43(0.40,0.45) | 0.37(0.35,0.40) | 0.29(0.27,0.31) |
|  |  | Yes | 0.39(0.37,0.41) | 0.26(0.21,0.32) | 0.34(0.32,0.37) |
|  | Waste others | No | 0.39(0.37,0.41) | 0.36(0.34,0.39) | 0.29(0.27,0.31) |
|  |  | Yes | 0.45(0.42,0.48) | 0.42(0.39,0.44) | 0.40(0.38,0.43) |
| Waste payment | | No | 0.39(0.37,0.41) | 0.37(0.35,0.40) | 0.29(0.27,0.31) |
|  |  | Yes | 0.45(0.43,0.47) | 0.36(0.33,0.39) | 0.30(0.28,0.32) |
| Environmental disaster | | No | 0.39(0.37,0.41) | 0.37(0.34,0.39) | 0.28(0.26,0.31) |
|  |  | Yes | 0.43(0.41,0.46) | 0.38(0.36,0.41) | 0.37(0.35,0.40) |
| **Enabling factors** | | | | |  |
| Income activity engagement | | No | 0.40(0.39,0.42) | 0.37(0.35,0.40) | 0.33(0.31,0.35) |
|  |  | Yes | 0.36(0.34,0.37) | 0.36(0.34,0.39) | 0.29(0.27,0.32) |
| Household tenure | | Tenant | 0.39(0.37,0.41) | 0.37(0.34,0.39) | 0.29(0.27,0.31) |
|  |  | landlord | 0.39(0.37,0.41) | 0.38(0.35,0.40) | 0.30(0.28,0.32) |
|  |  | Free living | 0.43(0.41,0.45) | 0.36(0.33,0.38) | 0.28(0.26,0.30) |
|  |  | Caretaker/lease/temporary stay/others | 0.40(0.37,0.42) | 0.36(0.33,0.40) | 0.28(0.26,0.31) |
| Sources of income | Business | No | 0.39(0.37,0.40) | 0.37(0.35,0.40) | 0.29(0.27,0.31) |
|  |  | Yes | 0.41(0.39,0.43) | 0.37(0.34,0.39) | 0.29(0.27,0.31) |
|  | fishing | No | 0.39(0.37,0.41) | 0.37(0.35,0.40) | 0.29(0.27,0.31) |
|  |  | Yes | 0.41(0.40,0.43) | 0.38(0.30,0.46) | 0.35(0.31,0.39) |
|  | Private salaried | No | 0.39(0.37,0.41) | 0.37(0.35,0.40) | 0.29(0.27,0.31) |
|  |  | Yes | 0.39(0.38,0.41) | 0.36(0.34,0.39) | 0.32(0.30,0.35) |
|  | Daily wage | No | 0.39(0.37,0.41) | 0.37(0.35,0.40) | 0.29(0.27,0.32) |
|  |  | Yes | 0.44(0.43,0.46) | 0.35(0.33,0.38) | 0.25(0.23,0.28) |
|  | Bike ride | No | 0.39(0.37,0.41) | 0.37(0.35,0.40) | 0.29(0.27,0.31) |
|  |  | Yes | 0.39(0.37,0.41) | 0.36(0.33,0.40) | 0.30(0.28,0.32) |
|  | Stone mining | No | 0.39(0.37,0.41) | 0.37(0.34,0.40) | 0.29(0.27,0.31) |
|  |  | Yes | 0.43(0.34,0.53) | 0.37(0.34,0.39) | 0.28(0.26,0.30) |
|  | Others | No | 0.39(0.37,0.41) | 0.41(0.38,0.43) | 0.29(0.27,0.31) |
|  |  | Yes | 0.42(0.41,0.44) | 0.37(0.35,0.40) | 0.27(0.25,0.30) |

AR (95%CI) = Absolute Risk (95% Confidence Interval); min = Minutes
