# Supplementary material for: Household determinants of healthcare utilisation in three informal settlements in Freetown, Sierra Leone: a cross-sectional survey
Source: BMJ Open. 2026 Mar 3;16(3):e108022. doi: 10.1136/bmjopen-2025-108022 (PMC12958868; doi:10.1136/bmjopen-2025-108022)
Supplement: Supplementary file 2 [file bmjopen-2025-108022.reviewer_comments.pdf]

PEER REVIEW HISTORY

BMJ Open publishes all reviews undertaken for accepted manuscripts. Reviewers are asked to complete a checklist review form and are provided with free text boxes to elaborate on their assessment. These free text comments are reproduced below.

This paper was submitted to another journal from BMJ but declined for publication following peer review. The authors addressed the reviewers' comments and submitted the revised paper to BMJ Open. The paper was subsequently accepted for publication at BMJ Open.

ARTICLE DETAILS

Title (Provisional)

The household determinants of healthcare utilisation in three informal settlements in Freetown, Sierra Leone: A cross-sectional survey.

Authors

Sesay, Samira; Sesay, Ibrahim Juldeh; Tengbe, Sia Morenike; Wurie, Haja; Fullah , Sullaiman; Vangahun, Dora; Gandi, Ibrahim; Teixeira de Siqueira Filha, Noemia; Lakshman, Rajith W. D.; Conteh, Abu; Saidu, Samuel; Koroma, Braima; Mansaray , Bintu; Elsey, Helen; Whittaker, Lana; Dean, Laura; Wiltgen Georgi, Neele; Nganda, Motto; Refell , Francis; MacCarthy, Joseph; Leyland, Alastair H.; Tolhurst, Rachel; Kibuchi, Eliud

---

VERSION 1 - REVIEW

**Reviewer**	**1**
**Name**	**Reviewer 1**
**Affiliation**	
**Date**	**25-Nov-2024**
**COI**	

Comments not published pending reviewer's consent.

**Reviewer**	**2**
**Name**	**Andres, Ellie**
**Affiliation**	**Duke-NUS Medical School**
**Date**	**25-Nov-2024**
**COI**	**None**

Thank you for the opportunity to review this paper. I appreciate the opportunity to learn about this setting and the focus on promoting healthcare access. Please find my comments/questions below.

Abstract

Perhaps reflect on results with Andersen framework as it is a bit hard to follow some of the associations without variable descriptions.

Introduction

- First sentence-- informal urban residents—needs to be clarified and think helpful to give reader more information here about the population group you are focusing on and perhaps their representation within the larger populations
- Line 42, omit 'In contrast'
- Third paragraph—make more clear how your research fits in within the existing body of research by Park et al (e.g. what else is know about HU among residents of informal settlements in Africa? How your study might differ from the existing research, etc to make the case for your study.)

o I would move up final sentence of 3rd paragraph so reader gets this info before you say there is not much research in Sierra Leone.

Methods

- When describing the settlements a map or table differentiating the three might be helpful if room allows
- The description of outcome variables could be streamlined while the reader could use additional information about what some of the explanatory variables mean (e.g. business versus private salaried, bike ride?) I realize they are provided in supplementary material but think it would be helpful to explain some that might be less obvious for general readers
- Why list all the explanatory variables if only using the ones indicated in the analysis?

Results

- I don't think we need to see everything in Table 1—maybe test for differences and indicate those instead to make your point

Discussion

First paragraph:

- This discussion of okadas should come earlier—guessing most readers will not be familiar

2nd paragraph:

- I'm not sure this warrants full paragraph—point taken in first sentence perhaps with ideas about why healthcare system less developed there. I would omit the final sentence

3rd paragraph:

- Definition of food security in the study should be described earlier—not discussion

The findings should emphasize novelty and be presented based on the Andersen framework which would help ground the discussion more and offer understanding for points of intervention. Focusing on bigger picture findings with wider applicability would make the manuscript more compelling. Likewise, reflection on variation in HU within and outside settlements and why this might be the case could provide additional understanding for the reader since this is the primary stratification for the analysis. Also, the authors mention that most participants are affected by environmental disaster and this probably warrants further consideration in the discussion.

VERSION 1 - AUTHOR RESPONSE

Reviewer: 2

Comments to the Author
Thank you for the opportunity to review this paper. I appreciate the opportunity to learn about this setting and the focus on promoting healthcare access. Please find my comments/questions below.

Abstract
Perhaps reflect on results with Andersen framework as it is a bit hard to follow some of the associations without variable descriptions.

R2.1 Introduction

- First sentence-- informal urban residents—needs to be clarified and think helpful to give reader more information here about the population group you are focusing on and perhaps their representation within the larger populations

In our analysis, health utilisation is at household level, and we are considering all members in the household. For clarification purposes, we have included the following sentence in the last paragraph of the Background section : "*Unlike previous studies that primarily focused on individual level factors, this article examines whether household levels determinants differ when healthcare is sought within or outside these communities. Accordingly, HU is assed for all members of each household".*

- Line 42, omit 'In contrast'

We have removed.

- Third paragraph—make more clear how your research fits in within the existing body of research by Park et al (e.g. what else is know about HU among residents of informal settlements in Africa? How your study might differ from the existing research, etc to make the case for your study.)

We have included the following paragraph "*Although studies in Southeast Asia and parts of Eastern and Southern Africa have explored individual level factors influencing HU in informal settlements, a critical research gap remains regarding the role of household-level factors (2). Household characteristics such as family income, water sources, and family size, and gender of head of household, affect all members and may influence healthcare seeking behavior (16). Moreover, understanding whether these factors differ when healthcare is sought within or outside communities would support in designing targeted interventions for improved health outcomes. This distinction is crucial, as healthcare seeking decisions within or outside a given area are influenced by access, cost, availability of services and quality of care (17). This issue is particularly important in Freetown, Sierra Leone, a city in a low-income West African country, where no previous research has examined the association of household-level factors and HU in informal settlements.*"

- I would move up final sentence of 3rd paragraph so reader gets this info before you say there is not much research in Sierra Leone.

Done.

R2.2 Methods

- When describing the settlements a map or table differentiating the three might be helpful if room allows.

We have included Figure 1

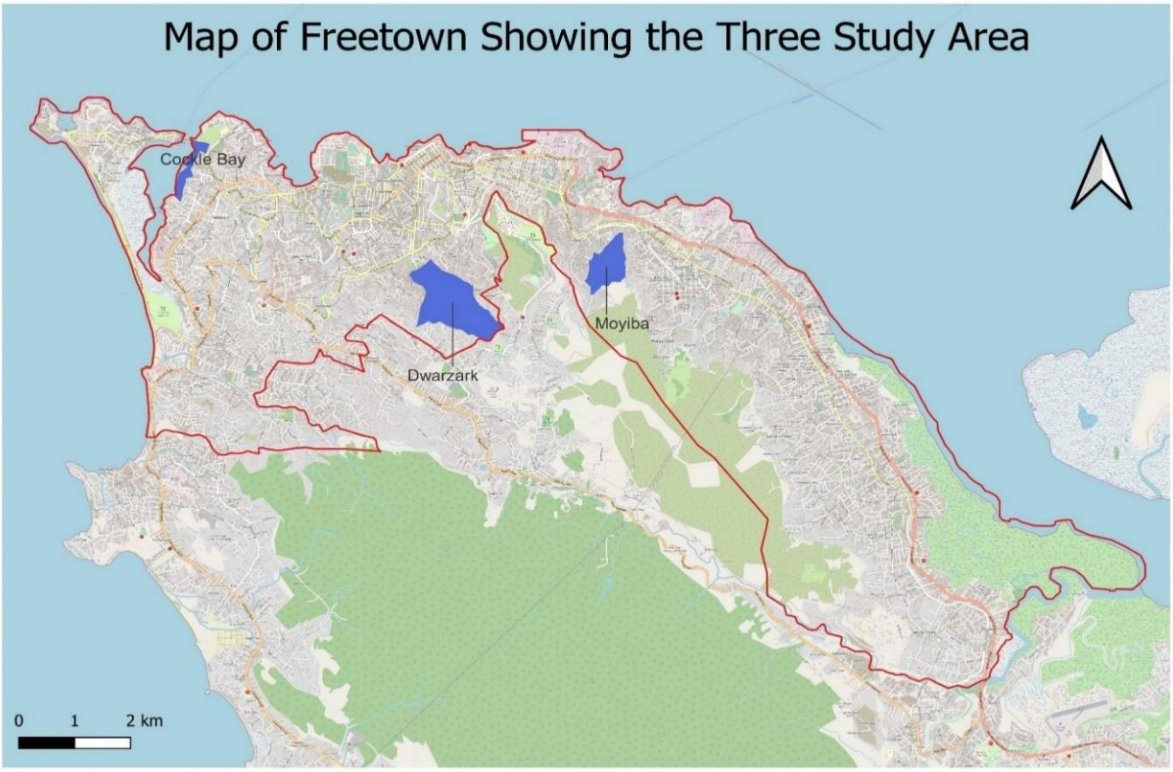

Figure 1: A map of three study areas (in blue)

- The description of outcome variables could be streamlined while the reader could use additional information about what some of the explanatory variables mean (e.g. business versus private salaried, bike ride?) I realize they are provided in supplementary material but think it would be helpful to explain some that might be less obvious for general readers

We have updated accordingly and included additional information as suggested .

• *Predisposing factors (household characteristics influencing HU) include the gender of the household head, family type, income-generating activity, presence of disability in the household (including whether the head of household is disabled), food security status (measured on whether a household could eat the kinds of food they preferred), length of residence in the settlement, household tenure (ownership or rental status), and environmental conditions (e.g. exposure of disasters). We also accounted for water access (e.g., piped water in dwellings, wells, public taps, rainwater, or sachet water) and sanitation facilities (e.g., flush toilets, pit latrines, bucket toilets, open defecation). Waste disposal methods (e.g., community waste collection, dumping sites, dumping of waste in drainages, or disposal into the sea for those living in coastal shacks) and whether households pay for waste collection are also considered.*

• *Enabling factors (socioeconomic conditions that facilitate or hinder HU) include sources of household income. Specifically, we distinguished between private salaried work (formal employment with fixed wages, such as company jobs), business (self-employment or entrepreneurship), and informal salaried work (casual or irregular wage labour). Other sources that were considered include fishing, daily wages, stone mining, and bike riding (commercial motorcycle transport, commonly known as 'okada').*

- Why list all the explanatory variables if only using the ones indicated in the analysis?

Revised and we now consider only variables used in the analysis.

R2.3 Results

- I don't think we need to see everything in Table 1—maybe test for differences and indicate those instead to make your point

We have updated analyse , and Table 1 now presents Chi-Square statistics and corresponding p-values for variables of interest across each informal settlements, separately for HU within and outside the settlement. The corresponding frequencies and percentages are provided in the Supplementary material Tables S2 and S3.

R2.4 Discussion

- First paragraph:
  - This discussion of okadas should come earlier—guessing most readers will not be familiar

  Due to updated analysis for each informal settlement, we have updated discussion section to reflect the new findings.

- 2nd paragraph:
  - I'm not sure this warrants full paragraph—point taken in first sentence perhaps with ideas about why healthcare system less developed there. I would omit the final sentence

  We have updated this section based on updated results.
- 3rd paragraph:
  Definition of food security in the study should be described earlier—not discussion
  Updated and food security definition is now included in Data subsection.

- The findings should emphasize novelty and be presented based on the Andersen framework which would help ground the discussion more and offer understanding for points of intervention. Focusing on bigger picture findings with wider applicability would make the manuscript more compelling. Likewise, reflection on variation in HU within and outside settlements and why this might be the case could provide additional understanding for the reader since this is the primary stratification for the analysis. Also, the authors mention that most participants are affected by environmental disaster and this probably warrants further consideration in the discussion.

We have updated discussion section to highlight how health utilisation patterns differ across each informal settlement when comparing HU within and outside the settlement and explored the underlying reasons for these differences . For instance, in relation to environmental disaster, we discuss the following:

"Environmental disaster were associated with reduced HU within settlement in Dwazark and Moyiba, both of which are hilly areas where disasters disrupt already fragile healthcare systems, impeding timely access to care (40). However, the association of environmental disasters on HU outside settlements was mixed. In Cockle Bay, disasters

were associated with increased HU, possibly due to the absence of sufficient local infrastructure, pushing households to seek care elsewhere. In contrast, in Dwazark, external HU decreased, potentially reflecting adequate within settlement response capacity during such events or barriers to mobility."

VERSION 2 - REVIEW

**Reviewer**	**2**
**Name**	**Andres, Ellie**
**Affiliation**	**Duke-NUS Medical School**
**Date**	**31-Jul-2025**
**COI**	

Thank you for the opportunity to review this paper again. Please find my comments below. I believe more closely integrating the Andersen framework and focusing on big-picture takeaway points applicable for a more general audience would improve the paper.

Introduction

- First paragraph: Rewrite for clarity.

- 3rd para: are the key individual factors associated with HU considered predisposing or enabling? If going to talk about Andersen's framework its easier to follow with that classification

o There is an extra 't' in third line

- 4th para: how do household factors studied here fit into Andersen's framework?

- Final para: how is this study guided by Andersen's framework specifically?

o Need to rewrite from second sentence on

o Why is it important to consider household factors in addition to individual—make explicit

o There is still not really a clear hypothesis as Reviewer 1 requested

Methods

- Explanatory variables: what is the difference between income-generating activity (predisposing) and sources of household income (enabling)

- It seems like it would be helpful for the reader to be more explicit about predisposing versus enabling to understand how all fit in to the model. It seems you are treating all factors the same in the modeling

- What is the rationale for analyzing the three settlements independently? Were they not sampled to be analyzed together? Perhaps the stratified results could be helpful at a local level but is difficult for the general reader to make sense of

Results

- Table 1 again seems like more info than is needed—what is the main takeaway?

- It would be helpful to use the Andersen framework to guide discussion of the results to understand how they fit in

- Head of household is not described as an explanatory variable in the methods

- The results seem like they might be helpful for community planning but not appropriate to be presented in this level of detail stratified by locality for general readers. What are the takeaways for general readers? What are the implication HU outside versus inside settlements?

Discussion

- Again, the findings should emphasize novelty and be presented based on the Andersen framework which would help ground the discussion more and offer understanding for points of intervention. Focusing on bigger picture findings with wider applicability would make the manuscript more compelling. Given the heterogenous results it is currently difficult to understand key takeaways.

**Reviewer**	**3**
**Name**	**Lee, Gwenyth**
**Affiliation**	**Rutgers School of Public Health, Epidemiology**
**Date**	**28-Oct-2025**
**COI**	**None**

I commend the authors on an interesting study related to an important topic. Understanding how household factors may impact health utilization behaviors across formal and informal settlements is quite interesting, and left me with something to consider about how built environments drive behaviors. I have some comments, summarized below:

Abstract results - Food insecurity decreases health utilisation within Dwazark (12%) but slightly increases (by 2%) in Moyiba. – is this 2% increase statistically significantly greater than zero?

Abstract Conclusion How this study might affect research, practice or policy – rather than stating that the "findings provide valuable insights", specify what these insights are. Rather than saying that the results, "offer a foundation for developing policies aimed at improving

healthcare utilization", specify what foundation is offered. Based on the evidence, what kind of support might benefit this population?

What this study adds – this statement is not grammatically correct

At various points the authors discuss how healthcare utilization may vary 'within versus outside' of communities. This idea of within/outside is a little unclear and needs to be explained further in the introduction. Is the authors' interest in understanding whether risk factors for lower HU vary between communities (i.e., some drivers may be more relevant in some communities and less in others)? Or is it in understanding how risk factors for lower HU may between households that are living without a certain boundary, versus those that are more 'suburban' (living at the edge of the community)? In the results it appears that the comparison is between those living in informal settlements, and those living in formal settlements..?

Related to the above point, I would encourage the authors to reflect further on absolute versus relative risk. If something like food insecurity is associated with poorer HU with a similar RR across communities, it may still be more important to communities where the prevalence of food insecurity is higher. While the analysis is ok relative risk, the research objectives actually seem more focused on identifying those risk factors that 'matter' more to some communities versus others, which is a question that is more related to absolute risk.

Please provide rationale for not pooling data from the 3 settlements and including settlement as a fixed/random effect (why are all analyses stratified?). Perhaps also justify why comparisons between within/outside informal settlements are made through stratified analyses, versus through testing of interactions terms.

The number of comparisons made in Tables 2 & 3 is really a lot. Please justify why no corrections for multiple testing are required. It seems as though the main interest seems to be in understanding whether risk factors for HU vary by location. But most of the risk factors are probably proxies for socio-economic status, time poverty, mobility, and other factors more directly related to HU. The use of Anderson's behavioral model is useful, and I would suggest even further synthesis (maybe adding a figure?) so that the discussion can further focus on what these different proxies are likely to 'mean' vis-à-vis HU.

I also think that evaluating absolute risk versus relative might help bring structure to the larger number of risk factors evaluated – by assessing which risk factors are associated with greatest (absolute) effects on HU in different groups, the relative importance of these factors on a per-community basis will become clearer.

Sample Size – this is a little confusing. As written it sounds initially like the current paper describes a secondary analysis of the Freetown Informal settlements survey, but then from the sample size onward it appears that the survey was in fact designed with HU as the primary, a priority outcome of interest. Please clarify whether the current paper reports the

results for the primary research question on which the data collection was conducted, or whether this is a secondary analysis.

VERSION 2 - AUTHOR RESPONSE

Reviewer: 2

Dr. Ellie Andres, Duke-NUS Medical School, Hong Kong University

Comments to the Author:

Thank you for the opportunity to review this paper again. Please find my comments below. I believe more closely integrating the Andersen framework and focusing on big-picture takeaway points applicable for a more general audience would improve the paper.

We are very pleased that Reviewer 1 is supportive of the paper and appreciative of the constructive feedback on how to approve the manuscript.

We have responded to reviewer comments below with responses highlighted in green.

Introduction

- First paragraph: Rewrite for clarity.

We have rewritten the first paragraph and this now reads: *"The need to provide timely and adequate access to healthcare for people living in urban informal settlements in low- and-middle income countries (LMICs) is of paramount importance, as they tend to have poorer health outcomes compared to their counterparts in rural and other urban areas (1-3). These poorer health outcomes are largely due to exposure to inadequate living conditions including limited basic facilities, low-quality housing, overcrowding, unsafe residential conditions, and pollution (1, 3). Despite being at high risk of adverse health outcomes, which in turn necessitates a greater need for healthcare utilisation (HU), no studies have examined the association of household-level factors and HU in these settings, even though they strongly shape access through shared vulnerability, constraints and decision making beyond what individual factors can capture. (2, 4)."*

- 3rd para: are the key individual factors associated with HU considered predisposing or enabling? If going to talk about Andersen's framework its easier to follow with that classification:

We have rewritten and it now reads: *"Studies have used Andersen's Behavioural Behavioural Model to guide the choice of factors associated with HU in informal settlements at the individual level (5-7). A systematic review by Park et al. (2) confirmed its applicability in this context, highlighting the influence of individual-level factors such as personal and biological, socioeconomic, cultural, religious, health systems, legal, and cognitive factors on HU (2). From this review, it is clear that many studies from LMICs have examined how predisposing, enabling, and need factors at the individual-level influence HU in informal settlements to a great extent (2). However, a critical research gap remains*

regarding the role of household-level factors in HU within these settings (2). Household-level factors can be incorporated into Andersen's framework, particularly in context of informal settlements, because shared living conditions within small and crowded spaces lacking basic services often influence resource pooling and healthcare-seeking decisions (2)".

- There is an extra 't' in third line

Deleted

- 4th para: how do household factors studied here fit into Andersen's framework?

We have added a paragraph which reads: *"For example, household characteristics such as water sources, type of toilet used, family size, and the gender of household head can be classified as predisposing factors, as they may affect the likelihood of seeking healthcare (16). Enabling factors, such as household engagement in income generating activities and the types of income sources, may determine whether a household member can afford healthcare when needed. Finally, existing conditions such as chronic illnesses or disability within the household can increase the likelihood of HU but also strain household resources, potentially limiting access for other members. In summary, understanding how household-level factors are associated with HU in informal settlements provides a more complete picture of utilisation patterns and can facilitate the design of effective, targeted interventions".*

- Final para: how is this study guided by Andersen's framework specifically?

We have revised and this now reads as follows: *"Guided by Andersen's Behavioural Behavioural Model, this study examines how household-level predisposing, enabling, and need factors influence HU inequalities. It explores associations between household-level factors and HU both within and outside three informal settlements (Cockle Bay, Moyiba, and Dwazark) in Freetown, Sierra Leone. HU is assessed for all members of each household, and findings will provide context-specific insights to inform policies that improve healthcare access and reduce inequalities in Freetown".*

- Need to rewrite from second sentence on Why is it important to consider household factors in addition to individual—make explicit

We have included this in the third paragraph: "Household-level factors can be incorporated into Andersen's framework, particularly in context of informal settlements, because shared living conditions within small and crowded spaces lacking basic services often influence resource pooling and healthcare-seeking decisions (2).

- There is still not really a clear hypothesis as Reviewer 1 requested

We have updated this in last paragraph: "Guided by Andersen's Behavioural Model, this study examines how household-level predisposing, enabling, and need factors influence HU inequalities. It explores associations between household-level factors and HU both within and outside three informal settlements (Cockle Bay, Moyiba, and Dwazark) in Freetown, Sierra Leone."

From the statement above, the null hypothesis would be: “Household-level predisposing, enabling, and need factors are not statistically associated with healthcare utilisation within or outside informal settlements in Freetown, Sierra Leone.” However, we recognise that current literature does not support judging the importance of a variable solely on statistical significance (8, 9). Therefore, we are focusing not only on significant variables but on the overall patterns of association across all variables considered. For this reason, we are not keen on including formal null and alternative hypotheses.

Methods

- Explanatory variables: what is the difference between income-generating activity (predisposing) and sources of household income (enabling)

We have revised and now consider income generating activities as an enabling factor because it represents the household’s financial capability, which determines whether healthcare can be afforded.

- It seems like it would be helpful for the reader to be more explicit about predisposing versus enabling to understand how all fit into the model. It seems you are treating all factors the same in the modelling

We have updated the Methods and Result sections to include income-generating activities and housing tenure as an enabling factor rather than predisposing one. Based on updates in the paper, we now believe that the distinction between predisposing factors (household characteristics influencing HU) and enabling (socioeconomic conditions that facilitate or hinder HU) factors is now clear.

- What is the rationale for analyzing the three settlements independently? Were they not sampled to be analyzed together? Perhaps the stratified results could be helpful at a local level but is difficult for the general reader to make sense of.

Initially, we had undertaken an analysis where informal settlement was included as an explanatory variable. However, during the first review, the reviewer noted that the three informal settlements are distinct and therefore likely to face different challenges in healthcare utilisation. We fully agreed with this comment, as HU patterns across each informal settlement may differ due to ease of access, affordability, service availability, and quality of care . Therefore, stratified analyses for each informal settlement were necessary to examine associations between household-level factors and HU. A clear understanding of association patterns at local level is crucial, since interventions can be more effectively tailored to each settlement based on the specific household-level factors that drive HU in that settlement.

Results

- Table 1 again seems like more info than is needed—what is the main takeaway?

Initially, Table 1 included only frequencies and percentages. However, during our first review, Reviewer 2 requested that we test differences in frequencies across variables, which is why we have included chi-square tests and the corresponding p-values. The corresponding frequencies and percentages are provided in the Supplementary Tables S2 and S3.

We have updated paragraph 1 in the Results section to read as follows: *"Table 1 presents chi-square test statistics and p-values for various predisposing and enabling factors associated with HU within and outside informal settlements, and highlights which household-level characteristics have statistically significant associations with HU."*

We have added the following statement at the end of the results interpretation for Table 1 as the main takeaway: *"In summary, HU in Freetown's informal settlements is shaped by household-level predisposing and enabling factors, but the associations vary by settlement and by whether care is sought within or outside the settlement."*

- It would be helpful to use the Andersen framework to guide discussion of the results to understand how they fit in.

We have updated the discussion section, and our finding are now discussed within Andersen's Behavioural framework, focusing on how household factors influence affordability, access, and the availability of local services.

- Head of household is not described as an explanatory variable in the methods

It is supposed to read "head of household gender" and not "head of household" which we have corrected.

- The results seem like they might be helpful for community planning but not appropriate to be presented in this level of detail stratified by locality for general readers. What are the takeaways for general readers? What is the implication HU outside versus inside settlements?

We have added the following statement at the end of the results explanation for Table 2: *"In summary, HU within informal settlements is influenced by household-level predisposing and enabling factors, but the strength and direction of these associations vary across settlements".*

For HU outside settlement, we have included the following sentence: *"In summary, findings in Table 4 show that household-level determinants that drive HU outside informal settlements are highly context-specific. Some factors such as disability and water sources show consistent associations across settlements, while others, such as income type, vary sharply by settlement. "*

Discussion

- Again, the findings should emphasize novelty and be presented based on the Andersen framework which would help ground the discussion more and offer understanding for points of intervention. Focusing on bigger picture findings with wider applicability would make the manuscript more compelling. Given the heterogenous results it is currently difficult to understand key takeaways.

We have updated the entire Discussion section and included a summary paragraph for both predisposing and enabling factors in the context of policy relevance.

Reviewer: 3

Dr. Gwenyth Lee, Rutgers School of Public Health

Comments to the Author:
I commend the authors on an interesting study related to an important topic. Understanding how household factors may impact health utilization behaviours across formal and informal settlements is quite interesting and left me with something to consider about how built environments drive behaviours. I have some comments, summarized below:

We are also very pleased that Reviewer 2 is supportive of the paper and appreciative of the constructive feedback on how to approve the manuscript.

We have responded to reviewer comments below with responses highlighted in green.

Abstract

- results Food insecurity decreases health utilisation within Dwazark (12%) but slightly increases (by 2%) in Moyiba. – is this 2% increase statistically significantly greater than zero?

It is not, since the credible interval contains 1.00 (i.e., 1.02(1.00-1.05)). However, it increases HU outside the settlement significantly. We have revised the text to read: "Food insecurity decreases HU within Dwazark (9%) and increases HU outside by 174% in Moyiba"

- Conclusion How this study might affect research, practice or policy – rather than stating that the "findings provide valuable insights", specify what these insights are. Rather than saying that the results, "offer a foundation for developing policies aimed at improving healthcare utilization", specify what foundation is offered. Based on the evidence, what kind of support might benefit this population?

We have revised this to read as follows: *"We identified strong settlement-specific patterns of Household-level factors that influence HU both within and outside Freetown's informal settlements. These findings provide a foundation for developing targeted policies such as strengthening local services, addressing affordability barriers, and supporting vulnerable occupation groups."*

- What this study adds – this statement is not grammatically correct

We have revised the entire section and aligned it with BMJ Open format by including the "Strengths and limitations" of the study. *"*

  - *Our findings provide valuable insights into the household-level determinants of healthcare utilisation (HU) across households in informal settlements in Freetown, Sierra Leone, drawing on the first large cross-sectional survey conducted in these settings.*
  - *The study identified settlement-specific associations between predisposing and enabling household-level factors and HU both within and outside Freetown's informal settlements.*
  - *The analysis was limited to household-level factors that are associated with HU and did not include individual-level factors due to the nature of the data.*
  - *As the study focused on three purposively selected informal settlements, the findings are not generalisable to all of Freetown's more than 60 informal settlements. "*
- At various points the authors discuss how healthcare utilization may vary 'within versus outside' of communities. This idea of within/outside is a little unclear and needs to be explained further in the introduction. Is the authors' interest in understanding whether risk

factors for lower HU vary between communities (i.e., some drivers may be more relevant in some communities and less in others)? Or is it in understanding how risk factors for lower HU may between households that are living without a certain boundary, versus those that are more 'suburban' (living at the edge of the community)? In the results it appears that the comparison is between those living in informal settlements, and those living in formal settlements?

We have revised the paragraph to make it clear that we meant. *"Moreover, understanding whether these predisposing and enabling household-level factors differ when healthcare is sought within an informal settlement (i.e., inside the settlement boundaries) or outside (i.e., beyond the boundaries) would support the design of targeted interventions to improve HU. This distinction is crucial, as it enables examination of how household-level factors influence healthcare seeking decisions based on ease of access, affordability, service availability, and quality of care (17). This issue is particularly important in Freetown, Sierra Leone, a low-income West African city, where no previous research has examined the association between household-level factors and HU in informal settlements."*

- Related to the above point, I would encourage the authors to reflect further on absolute versus relative risk. If something like food insecurity is associated with poorer HU with a similar RR across communities, it may still be more important to communities where the prevalence of food insecurity is higher. While the analysis is ok relative risk, the research objectives actually seem more focused on identifying those risk factors that 'matter' more to some communities versus others, which is a question that is more related to absolute risk.

We agree that absolute risk (AR) can help in understanding which household-level factors are of greatest importance in specific settlements. Since our analysis focused on assessing the strength of association between household-level factors and HU, both within and outside settlements, we decided to use relative risks (RR). We have included a clarification in the discussion (study limitations) that our analysis only focused on RR, and that policy relevance for these findings can be enhanced by also considering AR. Informed by this, we have provided AR for HU within settlements (Supplementary Table S4), outside settlements (Table S5), and both within and outside settlements (Table S7). However, we did not interpret or discuss these results because they fall outside the scope of our analyses, which aimed to examine associations.

We have included the following sentence in the Data analysis section "*We also computed absolute risks (AR), which are predicted probabilities of HU for each group, and presented them in the Supplementary Material."* and

"Fourth, while our analyses focused on RR, which explained associations between household-level factors and HU, the policy relevance of these findings can be enhanced by also considering absolute risks (AR). For example, being married, cohabiting, or engaged showed similar relative associations with HU within settlements, but its higher prevalence in Moyiba (0.4) compared to Dwazark (0.3) implies a greater absolute burden. Although this study primarily focused on RR we have provided AR in the Supplementary Material." in the Discussion section

- Please provide rationale for not pooling data from the 3 settlements and including settlement as a fixed/random effect (why are all analyses stratified?). Perhaps also justify

why comparisons between within/outside informal settlements are made through stratified analyses, versus through testing of interactions terms.

Reviewer one has also raised this point, and we have provided the following justification. Initially, we had undertaken an analysis where informal settlement was included as an explanatory variable. However, during the first review, the reviewer noted that the three informal settlements are distinct and therefore likely to face different challenges in healthcare utilisation. We fully agreed with this comment, as HU patterns across each informal settlement may differ due to different typologies and the availability or lack of health centres. Therefore, stratified analyses for each informal settlement were necessary to examine associations between household-level factors and HU. A clear understanding of association patterns at local level is crucial, since interventions can be more effectively tailored to each settlement base on the specific household-level factors that drive HU.

- The number of comparisons made in Tables 2 & 3 is really a lot. Please justify why no corrections for multiple testing are required. It seems as though the main interest seems to be in understanding whether risk factors for HU vary by location. But most of the risk factors are probably proxies for socio-economic status, time poverty, mobility, and other factors more directly related to HU. The use of Anderson's behavioural model is useful, and I would suggest even further synthesis (maybe adding a figure?) so that the discussion can further focus on what these different proxies are likely to 'mean' vis-à-vis HU.

Our analysis is theory-driven, guided by Andersen's Behavioural model framework, with the primary goal being interpretation of effect sizes rather than variable selection. That is, each variable considered is of interest on its own and therefore it is appropriate to test association with HU at the alpha value of 0.05 with no adjustment for multiple testing. This approach provides unbiased estimates that are important for understanding the magnitude and direction of associations. This makes application of multiple testing correction approaches less appropriate, as our interest lies in how different proxies of socio-economic status, time poverty, mobility, and other factors on their own are associated with HU across settlements. We acknowledge that estimates may be unstable when variables are highly correlated.

However, in the exploratory settings, where variable inclusion is not theory-driven, penalised regression approaches (such as ridge) would be appropriate, as they handle multiple comparisons by shrinking coefficients for stability. However, such approaches can also decrease associations especially weaker ones, which may obscure important relationships.

We have included Figure 2, which shows association between the variables considered and HU in the "Explanatory variables" section.

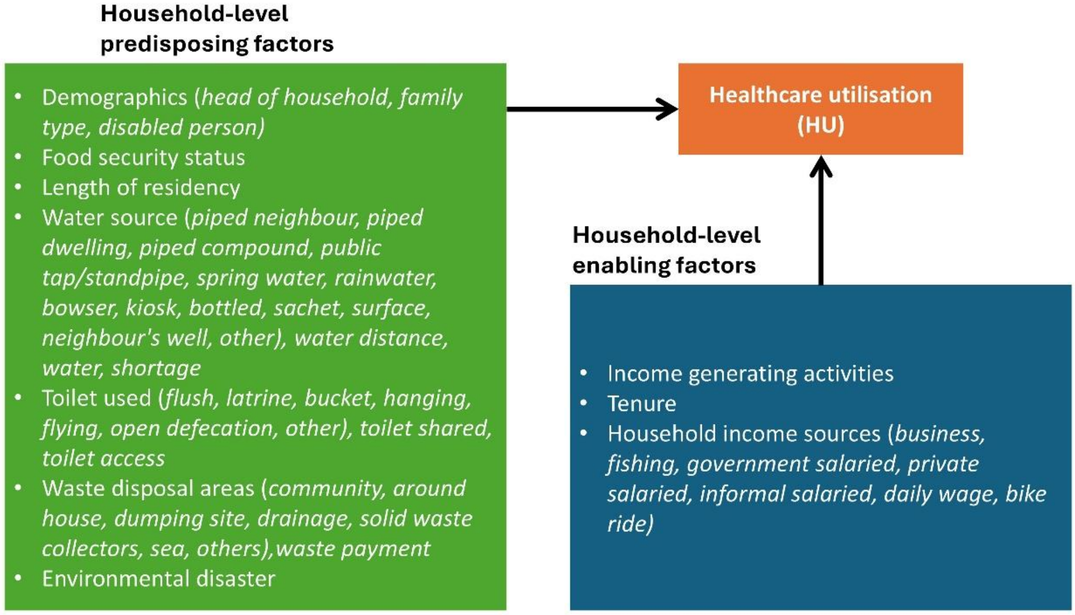

Figure 2: Application of Andersen's Behavioural Model to healthcare utilisation (HU) in informal settlements, showing household-level predisposing and enabling factors. (Note: the arrow indicates an associative, not causal, relationship.)

- I also think that evaluating absolute risk versus relative might help bring structure to the larger number of risk factors evaluated – by assessing which risk factors are associated with greatest (absolute) effects on HU in different groups, the relative importance of these factors on a per-community basis will become clearer.

We agree with this point and have provided sensitivity analysis for absolute risks as noted in the previous comment. While both RR and AR are valuable, presenting both in detail would be overwhelming within a single paper, particularly since our primary focus is on associations. Our analysis therefore concentrates on relative measures, while the sensitivity analysis offers additional insight into absolute risks.

- Sample Size – this is a little confusing. As written it sounds initially like the current paper describes a secondary analysis of the Freetown Informal settlements survey, but then from the sample size onward it appears that the survey was in fact designed with HU as the primary, a priority outcome of interest. Please clarify whether the current paper reports the results for the primary research question on which the data collection was conducted, or whether this is a secondary analysis.

The survey was designed with barriers of HU as a primary outcome as noted in Data *section "The sample size was determined based on the estimated proportion of households facing barriers to HU, which was 0.47 from a pilot survey. We used a margin of error of 0.03 and a design effect of 10 to account for the hybrid sampling technique (combining probability and non-probability sampling due to lack of an effective sampling frame), a critical value of 0.05 (95% confidence interval), and a non-response rate of 10%. The final sample size was 4,883 households, and further details about the sample size computation are provided in (15)."*

We have updated participants subsection of the abstract to read “Primary data from 4,871 households were collected during the Health and Wellbeing survey conducted between April and May 2023, targeting households with adults aged 18 years and older.”

We have updated data section to read as follows “Primary data collection for the 2023 Freetown Informal Settlements Health and Wellbeing Survey was conducted in Cockle Bay, Dwarzark, and Moyiba from April to May 2023, targeting households occupied by adults 18 years and older (20)”

We have also updated strengths and limitations to read “Our findings provide valuable insights into the household-level determinants of healthcare utilisation (HU) across households in informal settlements in Freetown, Sierra Leone, drawing on the primary data from the first large cross-sectional survey conducted in these settings.”

References

1. Corburn J, Karanja I. Informal settlements and a relational view of health in Nairobi, Kenya: sanitation, gender and dignity. Health Promot Int. 2016;31(2):258-69.
2. Park J-E, Kibe P, Yeboah G, Oyebode O, Harris B, Ajisola MM, et al. Factors associated with accessing and utilisation of healthcare and provision of health services for residents of slums in low and middle-income countries: a scoping review of recent literature. BMJ open. 2022;12(5):e055415.
3. UN-HABITAT. The challenge of slums: global report on human settlements, 2003: UN-HABITAT -United Nations Human Settlements Programme Staff; 2003.
4. Lilford RJ, Oyebode O, Satterthwaite D, Melendez-Torres G, Chen Y-F, Mberu B, et al. Improving the health and welfare of people who live in slums. The lancet. 2017;389(10068):559-70.
5. Pengid S, Peltzer K, de Moura Villela EF, Fodjo JNS, Siau CS, Chen WS, et al. Using Andersen’s model of health care utilization to assess factors associated with COVID-19 testing among adults in nine low-and middle-income countries: an online survey. BMC Health Services Research. 2022;22(1):265.
6. Adane M, Mengistie B, Mulat W, Kloos H, Medhin G. Utilization of health facilities and predictors of health-seeking behavior for under-five children with acute diarrhea in slums of Addis Ababa, Ethiopia: a community-based cross-sectional study. Journal of Health, Population and Nutrition. 2017;36:1-12.
7. Fayehun O, Ajisola M, Uthman O, Oyebode O, Oladejo A, Owoaje E, et al. A contextual exploration of healthcare service use in urban slums in Nigeria. Plos one. 2022;17(2):e0264725.
8. Amrhein V, Greenland S, McShane B. Scientists rise up against statistical significance. Nature. 2019;567(7748):305-7.
9. Trafimow D, Haley U, Boje D. Best way not to misuse p values is not to draw definitive conclusions about hypotheses. BMJ Evidence-Based Medicine. 2022.

VERSION 3 - REVIEW

**Reviewer**	**3**
**Name**	**Lee, Gwenyth**
**Affiliation**	**Rutgers School of Public Health, Epidemiology**
**Date**	**11-Dec-2025**

COI

The authors responses to my queries are sufficient.
